# Supporting caregivers of children living with disability in a humanitarian context: realist-informed evaluation of the ‘Mighty Children’ programme in Afghanistan

**DOI:** 10.1136/bmjgh-2023-012989

**Published:** 2024-09-10

**Authors:** Natalie Evans, Noorwhiza Ahmadi, Alice Morgan, Sadia Zalmai, Kate M Milner, Mohamed Faiz Atif, Hamish R Graham

**Affiliations:** 1 Melbourne Children's Global Health, Murdoch Children's Research Institute, Melbourne, Victoria, Australia; 2 Albury Wodonga Health, Albury, New South Wales, Australia; 3 Relief Humanitarian Development Organization (RHDO), Kabul, Afghanistan

**Keywords:** Child health, Paediatrics

## Abstract

**Introduction:**

To ensure that humanitarian action is disability-inclusive, evidence is needed to understand how different strategies to support children living with disabilities and their families can work in these settings. Evidence from other contexts suggests support groups can improve caregiver quality of life (QOL). This study reports an evaluation of the ‘*Mighty Children’* programme a participatory educational support group programme for caregivers of children living with disability in Kabul province, Afghanistan.

**Methods:**

We conducted a mixed-methods realist-informed before-and-after study to measure change in caregiver-reported QOL and explore how and for whom the programme worked, and in what contexts. Female caregivers of children with any disability were recruited through clinics in urban Kabul (n=3) and rural Paghman district (n=3). We collected quantitative data on QOL pre/post programme using the Paediatric Quality of Life Inventory Family Impact Module (PedsQL-FIM). Qualitative data were collected through facilitator and participant focus groups postprogramme.

**Results:**

118 caregivers participated in two cohorts (November 2020, February 2021). Caregivers expressed a significant increase in QOL from baseline to programme completion (t(125)=−10.7, p≤0.0001). Participation in cohort 2 was associated with the greatest PedsQL-FIM change.

Qualitative data revealed positive changes postprogramme in five key areas: caregiver mindset, parenting practices, disability-inclusive behaviours, psychological well-being and child functioning. These changes were seen as both outcomes and mechanisms influencing the primary outcome of QOL. Mechanisms that mediated these changes included increased knowledge of disability and the core acceptance and commitment therapy components of mindfulness and acceptance.

**Conclusion:**

The *Mighty Children* caregiver support programme for children living with disability in Afghanistan was associated with improved caregiver QOL. Further studies are warranted to explore pathways to scale, sustainability and potential application in other settings.

**WHAT IS ALREADY KNOWN ON THIS TOPIC**

- Caregivers of children living with disability experience a high burden of care and social isolation, particularly in humanitarian settings. Caregiver support groups have been shown to improve child and caregiver well-being in other contexts, yet there is little known about what works in humanitarian settings.

**WHAT THIS STUDY ADDS**

- Caregivers of children living with disability in Afghanistan reported improved quality of life, social inclusion and child functioning after participation in the *Mighty Children* programme.

**HOW THIS STUDY MIGHT AFFECT RESEARCH, PRACTICE OR POLICY**

- This study demonstrates that participatory educational support group interventions can be effectively implemented in humanitarian contexts to support caregivers and children living with disability.

## Introduction

### Disability in humanitarian settings

 Nearly 1 in 10 children worldwide lives with a disability,^1^ the vast majority of whom live in low-income and middle-income countries (LMICs).^2^ In humanitarian emergencies, children living with disabilities are among those most vulnerable to loss of food, shelter, healthcare, education and psychosocial support services, as well as risk of abuse.^3 4^ Conflicts and crises can both cause and compound disabilities due to the collapse of essential services and humanitarian assistance that is inappropriate, inaccessible or unsafe.^3 4^ In Afghanistan, a country in central Asia that has endured four decades of armed conflict, an estimated 3.5% of children live with severe disability.^5^ Stigma and discrimination are widespread and children with disabilities are often kept at home to protect them from abuse in the community.^6 7^ Access to basic health services is not equitable and has worsened over time^8^,^10^ with the approach towards disability continuing to be one of charity rather than encouraging participation and inclusion.^11 12^

In Afghanistan, childcare responsibilities are collective.^13^ Primary caregivers include not only mothers and fathers but also a wide range of other relatives that could change over time.^13^ However, caregiver roles are strongly gendered, with men typically fulfilling the role of the ‘breadwinner’ and women providing most of the home-based child care.^14^ Caregivers of children living with disability experience poorer mental and physical health compared with caregivers of typically developing peers.^15 16^ In LMICs, caregivers are often required to perform multiple roles due to a lack of accessible services and support and experience higher levels of anxiety, depression, physical exhaustion, stigma and isolation.^17^,^19^ While data in humanitarian settings are scarce, caregivers in one study described social isolation and a high burden of care.^20^ Compounding this, after decades of living with conflict and uncertainty, people in Afghanistan experience a substantial burden of mental ill health, with women and people living with disability at particularly high risk.^21^,^24^

Poor caregiver mental health has an impact on the cognitive development^25^ and mental health of the child.^13 26^ Caregiver mental ill health may also reduce the ability of caregivers to provide nurturing care.^1 25 26^ Nurturing care is essential for children in all settings to reach their full developmental potential.^25 27^ It is care that is responsive, developmentally stimulating and emotionally supportive while providing protection from threats and understanding children’s health and nutritional needs.^25 27^ In conflict-affected settings, the provision of consistent nurturing care can serve as a protective factor for child health and well-being.^27 28^ This highlights the need to support caregivers’ mental health and parenting skills as important pathways to influencing risk and resilience, particularly in conflict and postconflict settings.^13 29 30^

### Supporting caregivers

For the purposes of this paper, we define support groups as a forum for caregivers to learn about caring for their child, with the goal of improving caregiver interactions, behaviours, knowledge, beliefs, attitudes and/or parenting. Support group interventions have been shown to improve psychological well-being in caregivers of children with disability.^31^,^33^ Benefits may extend beyond psychological health, with improvements in parental self-efficacy,^34 35^ knowledge,^36 37^ parent–child interaction,^36^ child behaviour^35 38^ and child development.^39 40^ While parenting programmes are now common in high-income countries, there are few programmes developed and piloted for caregivers of children with disability in LMICs.^32 34 37^ Caregiver support groups in low-resource settings can be a cost-effective, sustainable way to access support when individual specialist care is scarce and expensive.^32 37^ Limited data from LMICs echo outcomes from higher-income settings including improved caregiver emotional well-being,^41^,^43^ knowledge^41 42 44^ and quality of life (QOL).^44 45^ Caregiver narratives post support group attendance describe moving from exclusion to togetherness, from emotional exhaustion to hope, and through gaining knowledge about their child’s condition understanding and acceptance.^18 43 46^

### ‘*Mighty Children’* caregiver support programme

We developed a context-appropriate evidence-informed support group programme, ‘*Mighty Children’* for caregivers of children living with disability in Afghanistan. We modified the content of an existing programme *Getting to Know Cerebral Palsy* (now known as Ubuntu)^47^ which has been evaluated in a range of country contexts for children with cerebral palsy and congenital Zika syndrome and has demonstrated improved caregiver well-being and confidence.^1842^,^44 48^ Ubuntu was first developed in 2011–2012 using action research to develop a participatory caregiver programme appropriate to the local context.^49^ The produced materials were seen as a ‘living document’ to be adapted to different contexts with the aim of empowering caregivers and increasing QOL.^49^

Adaptation of Ubuntu programme materials was an iterative process involving^1^ review of existing programmes and models of care,^2^ situational analysis (online supplemental annex 1),^3^ programme development meetings and workshops (online supplemental annex 2) and^4^ rapid cycle feedback and programme improvement during implementation.

We modified the content to be more generally applicable for children with physical disability beyond cerebral palsy, simpler for facilitation by non-health experts and added greater emphasis on psychosocial well-being and mindfulness strategies using culturally adapted methods from acceptance and commitment therapy (ACT).

ACT is a third-wave cognitive behavioural therapy that seeks to transform people’s relationships with difficult thoughts and emotions through three core strategies: mindfulness (being present in the moment), experiential acceptance (willing experience of emotions, cognitions and memories without attempts to change them) and valued action (engagement in meaningful, values-driven activities).^50^,^52^ Use of ACT has been associated with improved well-being in caregivers of children living with disability^53 54^ and has been successfully used in diverse cultural contexts.^55 56^

Over nine sessions, *Mighty Children* aimed to improve caregiver, child and family well-being through three core elements: (1) learning about caring for a child living with disability, (2) psychological resilience skills and (3) peer support (session description in online supplemental material 1). Through participatory methods, caregivers learn the practical elements of caring for children living with disability, like positioning, feeding, bathing and dressing, in addition to discussions about how to promote development (nurturing care), child rights and parenting strategies. Each module contains ACT components to develop psychological resilience and opportunities for sharing experiences among peers.

### Aims

This study aimed to (1) evaluate the *Mighty Children* support programme by measuring change in caregiver-reported QOL and (2) explore how and for whom the programme worked, and in what contexts. As this was a new programme piloted in a context with little pre-existing programme theory evidence, we approached our evaluation as a ‘theory-building’ exercise.^57^

In realist terms,^58^,^60^ our preliminary (middle-range) programme theory posited that:

In conflict-affected areas where health and social services are weakened (context) and existing social networks are disrupted (context), caregiver support groups may improve caregiver’s capacity for caring for children living with disability (mechanism) and enhance social connection with other children and families living with disability (mechanism), resulting in improved QOL for child and caregiver (outcome).

## Methods

### Research design

This mixed-methods evaluation involved before-and-after measurement of quantitative QOL outcomes triangulated with qualitative data^61^ to generate explanatory theories using a realist-informed approach.^58^,^60^ We used a realist-informed approach to generate theories about how the programme worked, recognising that any observed effects of our programme emerge from the complex interplay between context, programme activities, and the resources and responses that are put into action (mechanisms).^62^

Established checklists and guidelines were followed to ensure the quality of the realist,^59^ qualitative^63^ and mixed methods^64^ components of our study.

### Setting

We conducted this study in Kabul province, Afghanistan, during 2020 and 2021 (prior to the Taliban takeover) at a time of increasing insecurity. In partnership with the Ministry of Public Health in Afghanistan, we selected six basic health clinics to participate: three in urban Kabul and three in the rural district of Paghman (online supplemental figure 1).

The COVID-19 pandemic affected the training of facilitators, necessitating remote rather than in-person technical support. There were no pandemic impacts on the method of data collection or analysis.

### Intervention implementation

Our Kabul-based implementation team consisted of two coordinators (NA and SZ, female medical doctors) and two female facilitators (one psychologist and one nurse) who contributed to programme development, implementation, data collection and analysis. We recruited additional female healthcare workers (nurse or midwife) from each participating clinic as cofacilitators such that each group had one local clinic facilitator and one from our implementation team. Clinic facilitators were paid US$50 per month (roughly equivalent to one-third of the median healthcare worker salary^65^) and incorporated running the *Mighty Children* programme alongside their usual duties.

Facilitators attended 3 day-long workshops at a central office in Kabul, led by study coordinators (NA and SZ) with remote support from experts in Australia (NE, AM and HRG). Training covered programme content, group facilitation skills and data collection procedures. None of the facilitators had prior disability training.

One *Mighty Children* support group was established for each participating clinic (n=6). Beginning in November 2020, groups met weekly for 9 weeks, with sessions lasting 2–3 hours depending on group engagement and understanding. After the first programme, a second round was run in the same clinics (beginning February 2021) with minor programme modifications made by facilitators based on their experiences with the first groups. Facilitators used a structured manual in English/Pashto/Dari to guide each session and ensure programme fidelity.

### Caregiver participant recruitment

Participants were recruited in two rounds. For the first round, programme leads (MFA, NA and SZ) met with clinic and community leaders and held community information sessions. We took details of interested families at community meetings and added others who subsequently approached the clinics (convenience sampling). We accepted up to 10 caregivers for each group, capped to ensure effective participatory learning.^43^ The second round of groups included participants on the waitlist from round one, and additional registrants who had been sensitised by first-round participants (snowball sampling).

Group participants were female caregivers of children 2–10 years old living with a disability in the catchment area of participating clinics. Children were encouraged to attend and participate as able. We included only female caregivers, as it is mostly women who provide the bulk of childcare, and it would be culturally inappropriate to have mixed-gender support groups. Families self-identified as caring for a child with disability. No formal diagnosis of disability was necessary. There were no exclusions based on disability type or number of group sessions attended.

Participants were provided with a plain language statement (online supplemental annex 4) which was read to them by a facilitator. Caregivers and facilitators gave verbal informed consent (due to low literacy) for group participation and inclusion of their data in evaluation. We provided refreshments in group sessions but no other material incentives or travel support for participation.

### Patient and public involvement

Families were not involved in designing the intervention or research question. However, they were involved in the iterative improvement of the programme through endline feedback mechanisms.

### Data collection

#### Quantitative data

**Figure 1:**
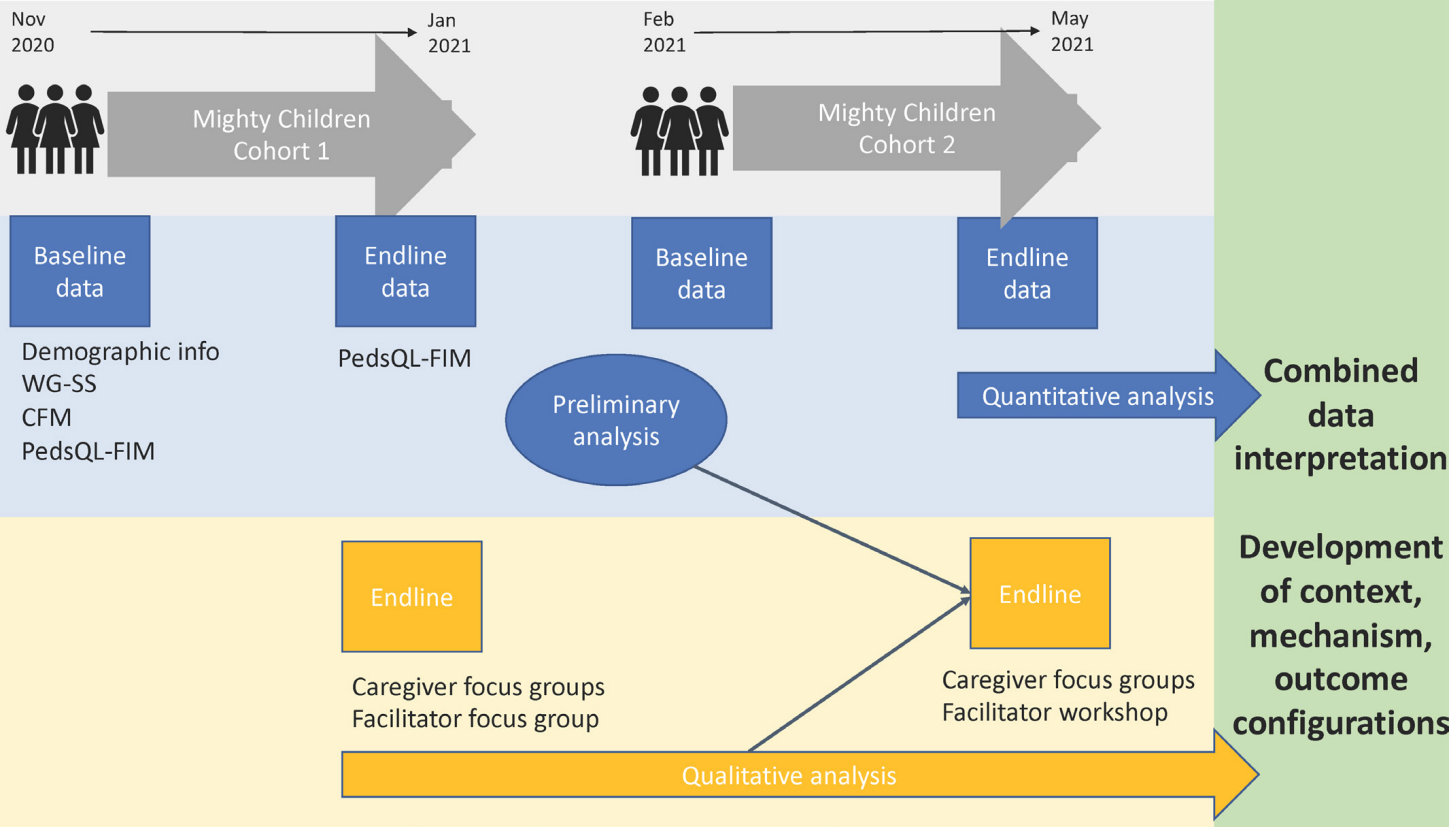
Data collection tools, procedures and analysis. Note: Demographic info, household demographic data; WG-SS, Washington Group Short Set (parents functional status); CFM, Washington Group/UNICEF Child Functioning Module; PedsQL-FIM, Paediatric Quality of Life, family impact module.

We obtained quantitative data from 118 caregivers at baseline and 116 at endline using surveys collecting sociodemographic information and validated measures for assessing functional impairment and QOL (see figure 1). Caregivers completed surveys in clinics assisted by facilitators (due to low literacy). Baseline data were collected after two sessions as the implementation team recommended accurate information would be more forthcoming once rapport had been built. Endline data were collected during the final session (session 9). All instruments were translated into Dari and Pashto by members of the implementation team, independently checked by an Australian-based academic fluent in English, Dari and Pashto and piloted prior to programme implementation (online supplemental annex 5 and 6).

Our primary outcome measure was the mean caregiver QOL score, measured using the Paediatric Quality of Life Inventory Family Impact module (PedsQL-FIM).^66^ PedsQL-FIM is a validated tool that has been used in other settings to measure caregiver health-related QOL and family functioning.^45^ The tool comprises 36 self-report items assessing caregiver physical, emotional, social and cognitive functioning, communication, worry, daily activities and family relationships, scored on a 0–4 Likert scale and linearly transformed (scale of 0–100) with higher scores indicating a higher QOL.^67^

Secondary outcomes included the number of caregivers who attended and percentage of participants who attended ≥6 sessions.

#### Qualitative data

Focus groups were conducted with caregivers and facilitators to explore what worked, for whom and how. We obtained qualitative data from 108 caregivers who attended 1 of 12 caregiver focus groups. Caregiver focus groups were conducted during the final session, led by their usual group facilitator in addition to at least one member of our implementation team (NA, SZ, WS and PA) using a semistructured guide (online supplemental annex 7). All focus group facilitators were novice interviewers who received basic training prior to the first round of focus groups and targeted retraining before the second round. We conducted debriefing after each focus group to collect facilitators’ impressions for our field notes.

Facilitators and coordinators participated in two group discussions. The first was a facilitator focus group, held after the first round of support groups to enable iterative improvement of the programme for round 2. Eight facilitators and one coordinator participated, and a programme coordinator (NA) led the discussion using a semistructured guide (online supplemental annex 8). The second group discussion was an endline workshop held with all available facilitators^8^ to discuss improvements to the programme after round two. Facilitators addressed the context of each module, with the discussion otherwise unstructured and moderated by a programme coordinator (SZ). Caregiver and facilitator focus groups ran for 20–90 min in Pashto or Dari, were audio recorded, then transcribed and translated into English by a professional translator.

### Data analysis

#### Quantitative

Quantitative surveys were completed on paper, entered into an online database (REDCap)^68 69^ by the study team in Afghanistan, then cleaned and cross-checked by a researcher in Australia (NE). Data were exported to Stata V.16 (StataCorp) for analysis.

We summarised demographic characteristics and functional status of participants by group location (rural/urban) and group cohort (one/two). Differences in PedsQL-FIM scores preintervention and postintervention were examined using paired sample t-tests. Primary effectiveness analysis compared differences in PedsQL-FIM scores preintervention and postintervention (PedsQL-FIM - PedsQL-FIM baseline) using paired sample t-tests. Secondary analyses compared mean differences in PedsQL-FIM score across key variables to explore potential demographic and implementation factors that influenced how the programme worked.

#### Qualitative

Translated focus group transcripts were coded and analysed using Nvivo V.12 (QSR International, Victoria, Australia), which has an embedded audit trail. Thematic analysis was used with a contextualist epistemology^70^,^72^ using a combination of inductive and deductive coding, to interrogate the data following our specific research questions. Six initial deductive categories (‘first reactions’, ‘lessons learnt’, ‘motivation’, ‘change: child, caregiver family’) allowed inductive creation of >20 codes as the data were explored. The number of codes expanded and contracted as the data were revisited repeatedly. A researcher (NE) with no prior experience with the study context performed initial coding. Coding strategy was cross-checked by two members of the authorship group (HRG and AM). Themes were proposed after examination of the data. Developing themes were reviewed by a researcher with mixed-methods and realist evaluation experience and familiarity with the Kabul context (HRG). Saturation was reached when themes were determined to be rich, and data were replicated with any negative cases explored.^73^ While direct member checking was not feasible, we enhanced trustworthiness by checking themes with group facilitators who had prolonged engagement with participants, and by presenting preliminary themes for discussion at professional fora.^74^

### Triangulation of quantitative and qualitative data

We used a realist-informed concurrent triangulation strategy^61^ (figure 1) to generate explanatory theories from our data.^58^,^60^ Our approach was iterative; qualitative and quantitative data were cross-checked for consistency and explored for possible mechanisms, allowing us to repeatedly review and refine our programme theory. For example, preliminary analysis of round one data raised questions that were explored through new lines of questioning in round two focus groups.

Qualitative themes were incorporated into context-mechanism-outcome configurations (CMOCs) with QOL as the primary quantitative outcome, however, also incorporating qualitative outcomes as they were drawn from the data. CMOCs were proposed and discussed with the broader research team (NE, HRG, AM and KMM) to ensure trustworthiness and refine our programme theory (see online supplemental annex 9 for preliminary CMOCs). CMOCs were reinterrogated alongside qualitative and quantitative data until agreement were reached.

#### Results

##### Participant characteristics

64 caregivers were recruited in cohort 1. 54 (84%) attended >6 sessions. Four (6.2%) caregivers withdrew prior to baseline data collection and two (3.1%) caregivers did not complete endline data. In cohort 2, 58 caregivers enrolled, none withdrew and 52 (90%) attended >6 sessions. Attendance numbers showed no attenuation over time.

Nine (7.6%) women had multiple children living with disability in their care, including one who provided care to three children living with disability.

Cohorts were similar across most demographic domains (table 1, additional detail in online supplemental table 1). However, rural participants tended to have higher home ownership, paternal education and likelihood of Pashtun ethnicity compared with urban participants.

**Table 1:** Household, caregiver and child characteristics

	Total	Urban	Rural	Cohort 1	Cohort 2
Household characteristics	**N=118 caregivers**	N=62	N=56	N=62	N=56
Median number of adults in house (IQ range)	3 (2–5)	3 (2–5)	3.5 (2–6)	3 (2–4)	3 (2–6)
Median number of children in house (IQ range)	3 (2–5)	3 (2–5)	3.5 (2–5)	3 (2–5)	3 (2–5)
Home ownership (%)	67 (56.8)	22 (35.5)	45 (80.4)*	31 (51.7)	36 (62.1)
Caregiver(s)†					
Mother (%)	106 (89.8)	52 (83.9)	54 (96.4)	55 (91.7)	51 (87.9)
Father (%)	45 (38.1)	31 (50.0)	14 (25.0)	22 (36.7)	23 (39.7)
Sister (%)	49 (41.5)	20 (32.2)	29 (51.8)	21 (35.0)	28 (48.3)
Brother (%)	8 (6.8)	0	8 (14.3)	4 (6.7)	4 (6.9)
Other family member—uncle, aunt, grandparent (%)	24 (20.3)	12 (19.4)	12 (21.4)	17 (28.3)	13 (22.4)
Parental characteristics					
Father age, mean years (SD)	40.5 (8.0)	41.7 (9.5)	39.2 (8.2)	41.6 (9.2)	39.4 (8.6)
Mother age, mean years (SD)	34.5 (7.0)	34.6 (7.1)	34.4 (6.9)	35.9 (7.3)	33.1 (6.4)
Father with disability (%)	23/111 (20.7)	15/58 (25.9)	8/53 (15.1)	12/57 (21.1)	11/54 (20.4)
Mother with disability (%)	27/116 (23.3)	18/60 (30.0)	9/54 (16.7)	17/60 (28.3)	10/56 (17.9)
Father ethnicity					
Pashtun (%)	58 (49.2)	15 (24.2)	43 (76.8)	30 (30.0)	28 (48.3)
Tajik (%)	26 (22.0)	19 (30.7)	7 (12.5)	12 (20.0)	14 (24.1)
Hazara (%)	26 (22.0)	20 (32.3)	6 (10.7)	14 (23.3)	12 (20.7)
Uzbek (%)	8 (6.8)	8 (12.9)	0	4 (6.7)	4 (6.9)
Mother ethnicity					
Pashtun (%)	55 (46.6)	15 (24.2)	40 (71.4)	26 (43.3)	29 (50.0)
Tajik (%)	30 (25.4)	20 (32.3)	10 (17.9)	16 (26.7)	14 (24.1)
Hazara (%)	26 (22.0)	20 (32.3)	6 (10.7)	14 (23.3)	12 (20.7)
Uzbek (%)	7 (5.9)	7 (11.3)	0	4 (6.7)	3 (5.2)
Father education	**n=117**	n=61	n=56	n=60	n=57
None/Madrassa (%)	54 (46.2)	34 (55.7)	20 (35.7)	24 (40.0)	30 (52.6)
Primary school/higher (%)	63 (53.8)	27 (44.3)	36 (64.3)	36 (60.0)	27 (47.4)
Mother education	**n=118**	n=62	n=56	n=60	n=58
None/Madrassa (%)	99 (83.9)	52 (83.9)	47 (83.9)	48 (80.0)	51 (87.9)
Primary school/higher (%)	19 (16.1)	10 (16.1)	9 (16.1)	12 (20.0)	7 (12.1)
Child characteristics	n=128	n=63	n=65	n=65	n=63
Mean age in years (SD)	7 (2.7)	6.8 (2.7)	7.1 (2.6)	7 (2.1)	7 (2.7)
2–4 years	29 (22.7)	16 (25.4)	13 (20.0)	13 (20.0)	16 (25.4)
5–7 years	34 (26.6)	17 (27.0)	17 (26.2)	20 (30.8)	14 (22.2)
8 years and above	65 (50.8)	30 (47.6)	35 (53.9)	32 (49.2)	33 (52.4)
Female (%)	53 (41.4)	29 (46.0)	24 (37.0)	30 (46.2)	23 (36.5)
Immunised (%)	120 (93.8)	59 (93.7)	61 (93.9)	63 (96.9)	57 (90.5)
Children with functional difficulty (%)‡	112 (95.3)	64 (98.5)	58 (92.1)	59 (93.7)	63 (96.9)
Child difficulties‡					
Seeing (%)	7 (5.5)	3 (4.8)	4 (6.2)	4 (6.2)	3 (4.8)
Hearing (%)	8 (6.3)	3 (4.8)	5 (7.7)	4 (6.2)	4 (6.4)
Walking (%)	86 (67.0)	47 (77.0)	39 (60.0)	44 (70.0)	42 (66.7)
Communication (%)	69 (53.9)	32 (50.8)	37 (56.9)	36 (55.4)	33 (52.4)
Learning (%)	70 (54.7)	29 (46.0)	41 (63.1)	33 (50.8)	37 (58.7)
Controlling behaviour (%)	56 (43.8)	25 (39.7)	31 (47.7)	29 (44.6)	27 (42.9)
Mental health§	66 (66.7)	27 (57.5)	39 (75.0)	41 (78.9)	25 (53.2)

Denominators are shown for variables with incomplete data.

Urban/rural and cohort comparison.

* One rural family lived in a tent.

† People responsible for caring for index child. Multiple caregivers could be selected.

‡ Measured using the Washington Group/UNICEF Child Functioning Module.

§ Anxiety and/or depression in children >5 years (N=99).

Characteristics of the children were largely comparable between cohorts and locations (online supplemental tables 1–4). For children under 5, difficulties with walking (N=19, 65%), communication (N=15, 52%) and learning (N=15, 52%) were the most reported (online supplemental table 2). While for children over 5, difficulty with walking (N=67, 69%) was followed by symptoms of anxiety (N=56, 57%) and depression (N=57, 57%) (online supplemental table 3).

### Primary outcome: caregiver QOL

Caregivers reported a significant improvement in their QOL and family functioning after the programme. This improvement was significant across all domains of the PedsQL-FIM (table 2). There were no significant differences in PedsQL-FIM scores between rural or urban groups at baseline or endline. However, there were significant differences between cohorts, with cohort 2 reporting much larger mean difference scores than cohort 1 (36 vs 8 point difference, p<0.001, table 3). This difference between cohorts was consistent across all domains of the PEDsQL-FIM (see online supplemental table 5).

**Table 2:** Mean PEDsQL-FIM scores

	Baseline (SD)	Endline (SD)	Mean difference (95% CI)	P value
Total score	29.6 (14.4)	51.3 (16.5)	21.7 (17.4, 25.9)	<0.0001
Physical functioning	30.8 (18.0)	55.6 (22.1)	24.9 (19.9, 29.8)	<0.0001
Emotional functioning	25.4 (17.9)	49.9 (20.1)	24.5 (19.4, 29.7)	<0.0001
Social functioning	36.8 (21.9)	60.3 (22.1)	23.5 (17.5, 29.5)	<0.0001
Cognitive functioning	28.6 (21.1)	46.6 (17.5)	18.0 (12.8, 23.2)	<0.0001
Communication	36.1 (24.9)	69.2 (27.9)	33.1 (26.2, 40.1)	<0.0001
Worry	12.7 (12.5)	32.9 (22.3)	20.2 (15.3, 25.0)	<0.0001
Daily activities	29.3 (21.0)	45.3 (22.4)	16.0 (10.4, 21.6)	<0.0001
Family relationships	41.1 (21.0)	56.2 (20.7)	15.1 (9.9, 20.3)	<0.0001
Parent health related quality of life summary score*	30.1 (16.2)	52.9 (16.9)	22.8 (18.3, 27.3)	<0.0001
Family functioning summary score†	36.7 (18.1)	52.1 (18.5)	15.4 (10.8, 20.1)	<0.0001

Higher scores indicate better functioning.

* Composite score including Physical, Emotional, Social and Cognitive Functioning scales.

† Composite score including daily activities and family relationships.

PedsQL-FIM, Paediatric Quality of Life Inventory Family Impact Module.

**Table 3:** Mean difference in PedsQL-FIM score by demographic and implementation subgroup factors

Variable	Value of category	Mean difference (95% CI)	P value
Cohort	Cohort 1	7.6 (2.4 to 12.6)	<0.001
	Cohort 2	35.8 (31.3 to 40.3)	
Group location	Urban	21.9 (16.3 to 27.5)	0.892
	Rural	21.4 (14.6 to 28.0)	
Household			
Number of adults in the home			0.415
Number of children in the home			0.467
Home ownership	Rent home	17.5 (10.5 to 24.5)	0.114
	Own home	24.4 (19.0 to 19.8)	
Total caregiver			0.105
Parents			
Ethnicity of mother	Pashtun	28.0 (21.9 to 34.1)	0.012
	Hazara	12.4 (1.8 to 23.0)	
	Other Persian	19.1 (12.4 to 25.7)	
Parent education	Some education	22.0 (16.9 to 27.1)	0.639
	No education	19.8 (11.6 to 28.0)	
Number of sessions attended			0.028
Child			
Age of child	2–4 years	28.2 (21.3 to 35.1)	0.172
	5–7 years	18.1 (10.8 to 25.3)	
	8 years and over	20.1 (13.9 to 26.3)	
Gender	Male	23.2 (18.2 to 28.2)	0.273
	Female	18.7 (12.1 to 25.3)	
Physical disability	Uses mobility aide	17.6 (12.7 to 22.4)	0.057
	No mobility aide	25.2 (18.9 to 31.5)	

P value for difference in means derived using t-test for binary variables, ANOVA for categorical variables and simple linear regression for continuous quantitative variables.

ANOVA, analysis of variance; PedsQL-FIM, Paediatric Quality of Life Inventory Family Impact Module.

Exploration of possible associations between demographic variables and PedsQL-FIM mean difference scores (table 3) demonstrated that maternal ethnicity had a significant relationship, with greater change evident in Pashtun mothers, compared with Hazara or other Persian caregivers (28 vs 12 and 19 point difference, p=0.012). There were no other significant associations found between PedsQL-FIM change and demographic variables, including child age, maternal age, maternal education status, economic status (home ownership) and household composition (number of children/adults/caregivers). A significant association was found between the number of sessions attended and the mean difference in PedsQL-FIM scores.

### Proposed context and mechanisms for change in QOL

#### Context: for whom did it work?

A key contextual factor arising from the quantitative data was cohort. Those caregivers in cohort 2 realised a larger improvement in QOL. Examination of the qualitative data suggests that this outcome could be due to facilitators’ increased knowledge, skills and confidence:

…we were not experts in the first group but when we started the second group we were experts. And we taught better than the first session [cohort]. – Anonymous facilitator.

Pre-existing caregiver knowledge may also have had a role. Some caregivers in cohort 2 entered the programme on the recommendation of friends or family members, coming with different knowledge and expectations:

It was also difficult for the participants [in Cohort 1] because it was a new thing for them. But about the second group [Cohort 2], when we started the program for them and they participated so they knew many things already because they had relation with the last group participants and had information from them.– Facilitator 5.

#### Mechanisms: how did it work?

In focus group discussions caregivers clearly articulated changes in themselves and their children that may help explain the large increase in QOL scores reported.

Before attending this program, there was frustration in my heart for my disabled child and this frustration nearly took away all my life, but after attending to this program, I learned how to be a positive or mighty mother. I learned how to take care of my mighty children, how to take care of my own self and now, as a powerful mother, I am ready to foster my mighty child, and fight against the negative thoughts of society about my mighty child. – Caregiver 112, Group 5B

**Figure 2:**
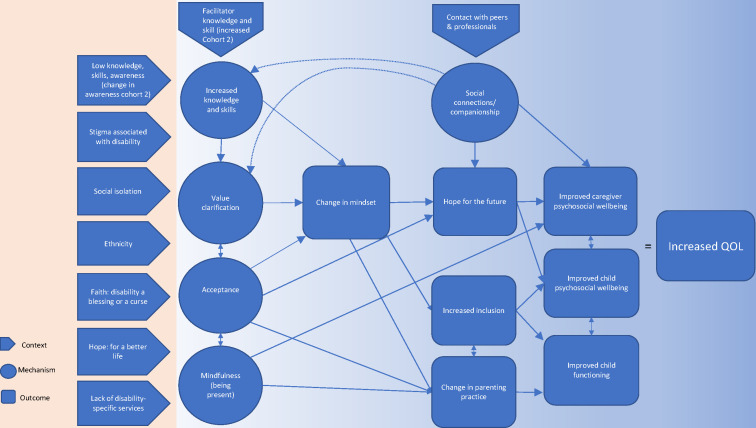
Context–mechanism–outcome–configuration (CMOC) for increased QOL. This CMOC is designed to give an overview of the contexts, mechanisms, outcomes (key themes/changes) identified through focus group discussions and their interrelations, including their hypothesised relationship with increased QOL. It necessarily simplifies complex and multidirectional relationships and is intended to be understood in reference to the rest of the paper. Please see online supplemental material 1 for individual CMOC for each key qualitative theme. QOL, quality of life.

We identified five main areas of change described by caregivers: change in mindset, parenting practice, inclusive behaviours, psychosocial well-being and child functioning. Each of these themes is seen both as outcomes in their own right and as mechanisms impacting the key quantitative outcome of QOL. For each theme, we describe context (C), mechanism(s) (M) and outcome(s) (O). Figure 2 provides an overarching CMOC, demonstrating interconnections between the themes and their relationship with the key outcome of QOL.

While there were many contextual factors and possible mechanisms impacting the five areas of change, we hypothesise that central among these were caregivers’ use of the ACT processes they learnt and practised through the programme—mindfulness, acceptance and value clarification (motivating value-driven behaviour). These psychological resilience tools enabled changes in caregivers’ attitudes and behaviours, allowing caregivers to act in a meaningful values-driven way to improve their QOL.

### Change in mindset: ‘our children are mighty [توانا ]’

A core change that underpinned the new skills, thoughts and behaviours caregivers described was a change in mindset. Caregivers’ attitudes towards their children shifted from frustration to value and love. Mechanisms contributing to this change seem to be gaining knowledge/awareness of caring for their child, alongside the ACT processes of acceptance of their current reality (emotional and physical) and values clarification. Our theory posits that in communities with high stigma and low knowledge about persons living with disability (C), caregivers who learn about disability (M), clarify their values (M) and develop acceptance (M), can change their mindset towards children living with disability (O).

We learnt that disabled children can also study and live like others. By doing this activity we realized that we can change our mentality regarding disabled children. – Caregiver (anonymous), Group 3A

Previously we did not give any value to our [children] but right now you’re giving them value. As you see my daughter does not have a hand. She cannot do her work properly, but as we attended to this program, this program taught us that disability is not weakness it is power and these children are mighty. *–* Caregiver 124, Group 6B

A particularly powerful mechanism appeared to be the use of inclusive language. The name of the programme itself—‘*Mighty Children’*—was chosen by facilitators during a planning workshop and was used repeatedly in groups and during feedback, emerging as a powerful catalyst for change. The name brought knowledge (that their children can do more than they expected) and power (that they are mighty).

Before people called my children disabled, ill, by the passing of time, and by the help of this program, now they call them mighty children. Here we can help our children to become someone. Here, they have found desire of becoming something. Now, we are not telling others that we have a disabled child, now we tell others that we have mighty children. – Caregiver 123, Group 6B

Faith provided an additional avenue towards acceptance for some caregivers. Living with faith appeared to provide a context for the knowledge and skills gained in *Mighty Children* to change the narrative for caregivers, from having a child ‘cursed’ with disability, to acceptance and confidence in their strength as a caregiver. Caregivers’ values became clearer, with priority placed on providing nurturing care.

We are living in the same house and same corridor with my in laws, she [mother in law] always used to say that my son is disabled and he cannot walk and Allah has given you such child then I [would] start fighting with her but now I deal with her with lots of patience and tell her that if Allah has given me such a disabled child, he also gave me the strength to take care of my child. – Caregiver 91, Group 3B

### Change in parenting practice: ‘speak with her kindly… give encouragement’

Caregivers brought with them culturally sanctioned parenting practices that accepted interpersonal violence in response to certain behaviours.^11 75 76^ Our theory posits that in the context of learnt punitive parenting practices (C), with increased parenting knowledge and skills (M), including tools of how to respond differently (behaviourally and emotionally) to challenging situations (M), caregivers can change parenting practices (M/O), acting in a values-driven way to increase caregiver and child QOL (O).

The most significant and important changes that have happened in me from the first session of this program till now is, taking more care of my child and I learned that we should treat these children the same as children that don’t have any disabilities. *–* Caregiver 69, Group 1B.

#### Nurturing care: ‘made them feel special’

Where previously children may have been insulted or ignored, now caregivers reported they were loved and encouraged, in many cases by their whole family:

Before participating in this program, me and my family members were insulting him and called him disabled. We had no idea what we should do with R_, but when I participated in this program and after learning all the lessons that Dr. Sahib taught us, my husband and my own behavior changed towards him, especially my behavior…I now understand that if I speak with him kindly, he will understand and may have better improvement. *–* Caregiver 94, Group 3B

#### Safe parenting: ‘taking care of my kids more seriously’

Caregivers reported that many of their children had previously experienced neglect or abuse because of their disability. Caregivers described the change that occurred:

Previously, I beat N_ a lot, I didn’t take care of her properly, I only thought about myself not thought about N_, but after coming to this program, I found patience, I found power to take care of myself and N_. Right now, I take care of her in a very good way. I love her, I help her to improve and become someone because disability is not a sin, it is power that can take you to your dreams. – Caregiver 127, Group 6B

Much of this change required caregivers to understand and manage their difficult thoughts and emotions such as anger, frustration and grief. The ACT skills of acceptance, value clarification and mindfulness allowed caregivers to develop the ability and confidence to respond differently to their own emotions and their children’s behaviour.

I have changed a lot when I became angry, I don’t beat or shout at my children, I only go out take some fresh air and make my mind calm then I come back, and I start playing with them. These were the changes that happened in me after coming to this program. – Caregiver 121, Group 6B

These changes were echoed by facilitators, who described how use of these strategies had changed their own home life and approach towards parenting:

The change in ourselves, we are also mothers, when the children would play at home, we would scream at them, we have changed a lot ourselves, our behaviour with our children*. –* Facilitator 4

### Increased inclusion: ‘we are mighty, we are proud’

*Mighty Children* gave many caregivers the knowledge and confidence to encourage their child to participate in home, community and school life. Our theory posits that when caregivers of children living with disability in a setting of high stigma and social isolation (C), gain the knowledge and tools (M) to change their mindset about their child’s condition (M/O), in a supportive environment with peer and professional role models (M), they develop psychological resources to deal with stigma and advocate for their child (M) resulting in greater inclusion in social settings (O).

#### Home life

Caregivers described eloquently how, after attending the programme, they were better able to involve their child in family life:

I see a lot of changes in the behaviour of my family, specially my mother-in-law who always used to curse me because of Y_’s disability, but now her knowledge has been increased because of this program and everyone in family shows more kindness and love for my son. – Caregiver 96, Group 3B

Other members of the family started to take more care of the children, relieving some of the burden of care away from the primary caregiver as well as increasing social inclusion for the child:

Whenever my child wants me to give him something, I prepare that thing for him as soon as I can. In my home, my family members take care of him more than me because they say that he has a problem and we must treat him as good as possible. My family members say, no one should say anything [bad] to him because God has created him in this way and we accept him as he is. – Caregiver 69, Group 1B

#### Community

Caregivers reported that their growing knowledge of disability and child rights gave themselves, and their family, the tools to correct misinformation and maltreatment:

I and my family have learned a lot of things but most importantly we have learned how to defend her rights against the negative thoughts of society. – Caregiver 89, Group 3B

This self-assurance among caregivers and families led to tangible differences for children—including being taken into the community with confidence.

Previously, we have not taken him anywhere because we were afraid that people would laugh at him, and tell that he has a mental problem and he is disabled; we did not let him to play in the street in front of others house because neighbours and their children were calling him mad, but now we don’t care what people call him, we do not take notice of them, and we let our mighty children go outside and be among people to get familiar with them, and become wise. – Caregiver 84, Group 4B

Before this program we were ashamed—when we took these children to a party, but now we take them to parties to show people that having a child with disability is not shameful. – Caregiver 127, Group 6B

#### School

Exclusion from school was a major burden for many caregivers and children, some of whom were motivated and able to enrol their child in school for the first time:

There were a lot of changes that took place in my life and my son’s life… The main changes that took place were, change in mindset of my son regarding his disability and passion. I enrolled my son into school after you advised me [during lessons, and my son learned a lot from his teachers and classmates. Now, he has a many friends. I was delighted to participate in this program. – Caregiver 80, Group 4B

### Psychosocial well-being: ‘our dead heart has revived’

Caregivers across all groups described improved emotional well-being after the programme. Our theory posits that for socially isolated caregivers with little knowledge of disability (C) and limited access to support services (C), making connections with peers and professionals (M), changing mindset about disability (M/O) and developing the psychological skills of acceptance and mindfulness to manage emotions (M), increases hope (M/O) and psychosocial well-being (O), contributing to improved QOL (O).

… they [caregivers] respected their children, gave up suicidal thoughts, and treated their children better, [it was] beneficial to them, their treatment of the children improved, they changed. – Facilitator 5

She [facilitator] taught us not to be sad, and she taught us how to get rid of sadness and sorrow, and showed us how to be happy and take ourselves from the cage of sadness and sorrow. – Caregiver 121, Group 6B

The initial feeling described by many caregivers on joining the programme was the sense they were not alone. Caregivers had the opportunity to forge friendships with others who were experiencing similar hardships, thus validating their own experiences and emotions:

Positive thing is that we are coming here and this is a big support for our psychology and our way of thinking because us we share our problem together our burden become less. – Anonymous caregiver, Group 3A

Because these mothers are the people who have the same problem that I have and they can understand my feelings more than others that is why we can be best friends. *–* Caregiver 114, Group 5B

Improved well-being was also experienced by children. For some, this could be attributed to change in caregiver mindset and use of inclusive language:

you have told us not to tell our children that they are disabled and useless in society, so we stopped cursing them and their confidence level got higher and got stronger. – Caregiver 89, Group 3B

#### Hope: ‘we see a future’

Hope seemed to act as both context and mechanism for improved psychosocial well-being. Despite substantial obstacles, many caregivers were motivated by hope to bring their children to the programme with the goal of a better life:

I want to be very honest with you, I was not scared or worried. I came here by my own choice having the hope that my child will become healthy like other children. – Caregiver 96, Group 3B

At the programme’s end, caregivers across all groups described gaining renewed hope for the future:

I didn’t have any hope for my son because he is deaf and dumb. Everyone told me that he couldn’t learn anything, but I help him to learn Holy Quran, and I teach him by my own. From the day that I attended this program, I found hope and I’m not hopeless anymore. I’m very thankful to your program that teach something to my son to become something in his life. – Caregiver 126, Group 6B

### Child functioning: ‘getting better day by day’

Some caregivers reported improvements in their child’s function as they provided positive opportunities to try new things and children developed increased self-confidence. Reported functional changes included improved mobility, communication and self-care. Our theory proposes that for caregivers with low knowledge of disability (C) and limited access to support services (C), increasing caregiving skills, knowledge and confidence (M), as well as building relationships with peers and professionals (M) can improve caregiver psychological well-being (M/O), change parenting practice (M/O) and mindset about disability (M/O). These changes can lead caregivers to value (M) and include (M/O) their child(ren) more, which is important for child self-confidence (M/O) and can lead to improved functioning (O) and QOL (O).

When I put my child on the carpet he did not use to move but after the exercises he started moving and now he can move slowly through the door or the place I am. – Caregiver 91, Group 3B

We chose this program to cure our children, before “Mighty Children” program my daughter didn’t talk, but now she can talk, play with other children, talk with other children. – Caregiver 124, Group 6B

For other families, expectations at the end of the programme were framed more around improvement and emotional well-being, rather than being ‘normal’.

We want our children to improve, to learn how to draw a picture. If he wasn’t able to draw a picture we must not tell him that you don’t know drawing why you draw such thing, instead we must help them, encourage them to do their best next time. At last, we must keep all these mighty children happy. – Caregiver 125, Group 6B

## Discussion

*Mighty Children* is the first support group, to our knowledge, designed and piloted for caregivers of children living with disabilities in a humanitarian setting. At the conclusion of the *Mighty Children* programme, caregivers reported a significant improvement in their QOL and family functioning, in keeping with studies of support groups for caregivers of children living with disability conducted in other countries.^42 45 77^ While there are many contextual factors and mechanisms that likely influenced this outcome (see figure 2), we will focus in this discussion on the key elements that arose from the examination of our data.

### For whom did it work?

Participating in the *Mighty Children* programme was associated with improvement in caregiver QOL irrespective of geographical location, wealth, family composition or ethnicity. While overall attendance was high, exploratory analysis suggested greater benefits for those who attended more sessions and those who were part of the second cohort. The same facilitators were used in both cohorts, and by the second round they reported having more knowledge, experience and confidence. We used rapid cycle feedback from caregivers and facilitators to improve the programme in real time, resulting in a second programme iteration that may have made information more accessible and/or meaningful to caregivers. In addition, many of the caregivers enrolled in the second cohort had heard of *Mighty Children* by word of mouth, including perhaps some core messages of the programme. This could have resulted in a group of caregivers with different expectations and/or who were primed by the first group in key concepts. We propose in our CMOC that facilitator experience was a key contextual factor that had an important bearing on the development of caregiver knowledge and skill.

We also observed that Pashtun caregivers described a larger improvement in QOL compared with other ethnicities. This echoes findings from a community-based rehabilitation programme in Afghanistan that showed greater improvements in mobility, activities of daily living and social and community life for Pashtuns compared with Tajik and minority ethnic groups.^78^ Pashtun participants in *Mighty Children* expressed significantly lower PedsQL-FIM scores at baseline compared with other ethnicities (F(2,115)=9.91, p=0.0001 see online supplemental table 6). This may indicate a higher baseline level of stigma, social exclusion and mental distress in Pashtun people living with disability.^79^ Pashtun is constrained by the *Pasthtunwali,* a traditional ethical code which places particular emphasis on fulfilment of specific social roles such as marriage or employment. If a family member cannot contribute because of his or her disability, this has an important bearing on their status in the family and community, and this social exclusion leads to mental distress.^79^ *Mighty Children* was a safe space for families that promoted inclusive behaviours in participants. We propose that this mechanism of inclusion worked more powerfully in the context of Pashtun culture, highlighting the importance of considering cultural context when thinking about how caregiver support groups work.^41^

### How did it work?

Five core areas of change were described by caregivers in an Afghan context: change in mindset, psychosocial well-being, parenting practices, inclusion and child functioning. These themes were inter-related and strongly connected to the ACT skills acquired during the programme.

The change in mindset that caregivers reported after the programme has been described in other support groups.^41 46^ This is exemplified by the embrace of the term ‘Mighty Children’ by caregivers, children and facilitators, illustrating the power of language in giving strength, confidence and hope—and ultimately changing views towards disability.^80^ Acceptance was a key mechanism that we intentionally targeted using culturally adapted ACT strategies, and from our qualitative data, it appeared to be interpreted in the context of faith. Faith could offer caregivers an avenue for acceptance, a source of meaning, and a way to understand their child’s condition.^41^ This goes beyond previous observations of faith as a coping mechanism,^46 80^ supporting suggestions that mindfulness strategies can become more meaningful when participants link them to their own religious and spiritual beliefs^53 81^ and illustrating the compatibility of ACT approaches with Islamic faith.^53^

Psychological well-being has been recognised as an important outcome in other support groups.^31 32^ In our study, we identified connections between psychological well-being, mindfulness, acceptance, social connectedness and hope. Hope is a predictor of well-being in all its forms.^82^ By connecting and learning with others in a similar situation who are doing well, caregivers can move from feeling isolated to finding strength and hope for the future.^18 43 46 83^ However, peer support alone may not be enough. A recent Cochrane systematic review of peer support groups for caregivers of children with complex needs found that while caregivers perceive peer support programmes as valuable, there was no evidence of benefit.^83^ Conversely, previous work suggests that including ACT in parent support programmes benefits caregivers over and above programmes with information and peer support alone.^33 84^

Caregivers identified multiple changes in parenting practice, with the impact on family violence highlighted strongly. While Mighty Children was not designed to combat family violence, we explicitly included modules on positive parenting and child safeguarding, conscious of cultural context. In Afghanistan, violence towards children is socially accepted by parents, communities and children themselves.^75 76 85^ 74% of Afghan children experience physical punishment,^11^ and children with disability are at particularly high risk.^86 87^ However, our study shows that interpersonal violence is rejected by parents in principle, and people are open to change.^85^ Caregivers used ACT strategies to recognise and respond to their own emotions, developing ‘tolerance’ and ‘patience’ so that they could be responsive caregivers towards their children.^88^ This supports findings that parenting programmes can help deepen attachments between caregivers and their children, promote positive parenting practices^26^ and reduce adverse child experiences^25^ while ACT strategies give caregivers tools to increase awareness of their own emotional states and triggers and reduce violence towards their children.^13 89^ With increasing recognition of family violence as a key public health problem globally,^25^ this is a strongly positive message—especially for humanitarian settings, where family stressors are high and protective systems are fractured or absent.^28^

Inclusion is a critical outcome, with many studies showing that caregivers of children living with disability experience aloneness, stigma and discrimination^20 46^ with resultant poor mental health.^26 79^ Our study found evidence of increased social participation at home, school and in the wider community, perhaps demonstrating the power of building support and understanding from within the family unit.^89^

Improvement in child functioning was a common hope or expectation among caregivers and while our programme included parental encouragement of child physical exploration and inclusion in family activities,^33^ we did not expect children to realise substantial functional gains, particularly in the short term. While some families reported improvements in their child’s mobility, communication or ability for self-care, it is not possible to know from our data if true gains were made or if these reports reflected alterations in parental perception.^41^

### Limitations

There were several methodological limitations due to literacy and cultural considerations: (1) use of the self-report method, particularly spoken out loud, risks social desirability bias; (2) use of facilitators as data collectors may have increased social desirability bias; (3) caregiver participants joining the programme through ‘word of mouth’ may have led to selection bias; (4) our baseline data were not collected at a true baseline, but after groups had already begun, however, this speaks more strongly for the strength of our results as change may have already begun; (5) analysis of qualitative data was performed on transcripts translated into English, which has an impact on the trustworthiness of the data.^90^

Our study has limitations to generalisability due to use of an uncontrolled pre–post test design and use in a limited rural and urban setting in Afghanistan. Without a control group, we are unable to infer causation in our evaluation. The authors all had input into the design, implementation and evaluation of the programme, leading to the possibility of confirmation bias in our evaluation.

### Future directions

Future studies should include impact evaluations using control groups and direct measurements of change in children. Preintervention interviews could examine cognitive mechanisms and baseline level of parenting knowledge. Repeated measurements at different time points should be taken to address the question of possible fading of effects seen in other interventions.^91 92^ Additional studies should examine the feasibility of taking this or similar interventions to scale, including considerations of cost-effectiveness and sustainability within healthcare systems. Measures of inclusion should also be explored, for example, school enrolment information.

Other programmatic elements trialled in other contexts such as using caregiver facilitators^93^ and making the programme accessible/applicable for male caregivers^94^ were thought not to be feasible in this pilot programme but should be explored in this and other humanitarian contexts.

## Conclusions

On completion of the *Mighty Children* support programme, caregivers of children living with disability reported significantly increased QOL. This has important implications for organisations working with vulnerable families who have limited access to services and support in humanitarian contexts, as it demonstrates that use of a participatory educational support group intervention can be successful in improving QOL. Our qualitative results suggest that this kind of parenting programme may not only positively influence psychosocial well-being of caregivers and their children but may also have broader applications in promoting inclusive practices, increasing nurturing care and reducing interpersonal violence. There appeared to be particular benefit in including ACT strategies within an educational programme. Mechanisms for changes described by caregivers included not only improved knowledge and skills, but also psychological resilience strategies (mindfulness and acceptance), enabling caregivers to recognise and respond to their own emotions to improve parenting practices and well-being.

Our study contributes to the limited data on disability in humanitarian settings and provides further evidence of the importance of supporting caregivers to provide nurturing care.

## Supplementary material

10.1136/bmjgh-2023-012989 online supplemental material 1

## Data Availability

Data are available on reasonable request.
