# Supplementary material for: Supporting caregivers of children living with disability in a humanitarian context: realist-informed evaluation of the ‘Mighty Children’ programme in Afghanistan
Source: BMJ Glob Health. 2024 Sep 10;9(9):e012989. doi: 10.1136/bmjgh-2023-012989 (PMC11404242; doi:10.1136/bmjgh-2023-012989)
Supplement: online supplemental material 1 [file bmjgh-9-9-s001.pdf]

MIGHTY CHILDREN SUPPLEMENTARY MATERIALS

Table of Contents

FIGURE 1: CLINIC LOCATIONS 3

TABLE 1: GROUP PARTICIPANTS ADDITIONAL DEMOGRAPHIC DETAIL 3

TABLE 2: NUMBER (AND PERCENT) OF FUNCTIONAL DIFFICULTIES[1] IN PARTICIPATING CHILDREN AGED 2-4 YEARS OLD 5

TABLE 3: NUMBER (AND PERCENT) OF FUNCTIONAL DIFFICULTIES[1] IN PARTICIPATING CHILDREN AGED 5-12 YEARS OLD 5

TABLE 4: NUMBER (AND PERCENT) OF CHILDREN AGED 2-12 WHO USE ASSISTIVE DEVICES 6

TABLE 5: COHORT COMPARISON OF PEDSQL MEAN DIFFERENCE SCORES (STANDARD DEVIATION) 6

TABLE 6: PEDSQL SCORES BY ETHNICITY. MEAN (SD). 7

ANNEX 1: SITUATIONAL ANALYSIS 8

ANNEX 2: PROGRAM ADAPTATION AND DEVELOPMENT 8

- Local Advisory board 8
- Program Development Workshop 9

ANNEX 3: PROGRAM DESCRIPTION 10

ANNEX 4: PLAIN LANGUAGE STATEMENT 11

- Information for Caregivers and Facilitators 14
- What is the *Mighty Children* program? 14
- Why should I join a *Mighty Children* group? 14
- Why is this for parents/caregivers? 15
- Why focus on children with disability? 15
- Who can attend the group? 15
- What happens in the *Mighty Children* groups? 16
- Who will facilitate the groups? 16
- چی کسی این گروپ ها را راهنما یی می کند؟ 16
- What will I need to do? 16
- Will we receive rehabilitation, medications, or medical equipment? 17
- Are there any risks to me? 17
- What will happen when the program ends? 18
  - *More about the Mighty Children program* *19*

ANNEX 5: QUANTITATIVE SURVEY INSTRUMENTS 21

- Family information – English 21
- Family information – Dari 35
- Family information – Pashto 50
- PedsQL Family Impact - Dari 11
- PedsQL Family Impact - Pashto 15
- PedsQL Family Impact - English 19

ANNEX 6: PILOTING AND ADAPTATION OF PARENTING SENSE OF COMPETENCY SCALE (PSOC) 22

- Original PSOC - Dari 22
- Original Parenting Sense of Competence Scale (PSOC) – (Pashto) 24
- Original Parenting Sense of Competence Scale (PSOC) - English 27

Modified PSOC ... 29
Modified PSOC – English ... 30
Modified PSOC - Dari ... 30
Table 10: Parenting sense of competency, mean (SD). Pooled data. ... 31
Table 11: Parenting sense of competency, by cohort. Mean (SD). ... 31
Table 12: Parenting sense of competency, by location. Mean (SD). ... 31

ANNEX 7: CAREGIVER FOCUS GROUP GUIDE ... 32

ANNEX 8: FACILITATOR FOCUS GROUP GUIDE ... 35

ANNEX 9: EARLY CONTEXT, MECHANISM, OUTCOME CONFIGURATIONS ... 38

Context, mechanism, outcome configuration (CMOC) including all identified themes ... 38
Change in mindset context, mechanism, outcome configuration (CMOC) ... 38
Proposed Context and Mechanisms for the key outcome change in mindset ... 39
Change in parenting practice context, mechanism, outcome configuration (CMOC) ... 41
Proposed Context and Mechanisms for the key outcome change in parenting practice ... 41
Inclusion context, mechanism, outcome configuration (CMOC) ... 43
Caregiver and child psychosocial wellbeing context, mechanism, outcome configuration (CMOC) ... 43

ANNEX 10: AUTHOR REFLEXIVITY STATEMENT ... 44

Figure 1: Clinic locations

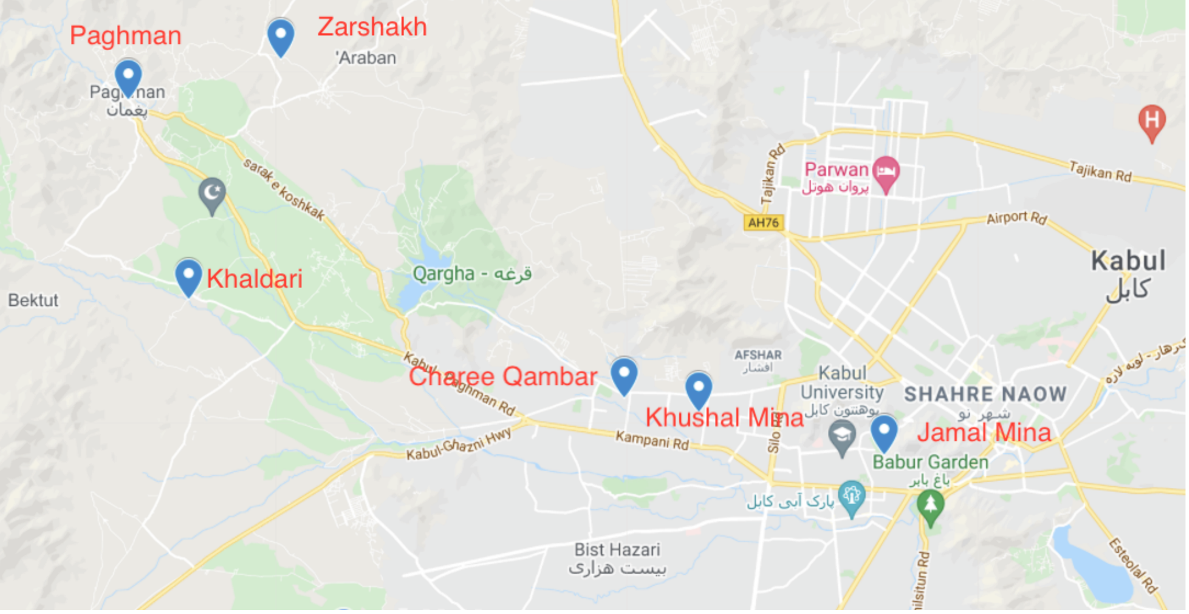

Table 1: Group participants additional demographic detail

	Total	Urban	Rural	Cohort 1	Cohort 2
**Household characteristics**					
Own home (%)	67 (56.8)	22 (35.5)*	45 (80.4)*$^{\alpha}$	31 (51.7)	36 (62.1)
Earthen floor (%)	106 (89.8)	4 (6.5)	7 (12.5)	58 (93.5)	48 (85.7)
Radio (%)	23 (19.5)	15 (24.2)	8 (14.3)	9 (15)	14 (24.1)
TV (%)	101 (85.6)	54 (87.1)	47 (83.9)	52 (86.7)	49 (84.5)
Phone (%)	109 (92.4)	56 (90.3)	53 (94.6)	56 (93.3)	53 (91.4)
Generator (%)	7 (5.9)	2 (3.2)	5 (8.9)	7 (11.7)*	0*
Fridge (%)	36 (30.5)	24 (38.7)*	12 (21.4)*	16 (26.7)	20 (34.5)
**Mode of transport**					
Own car/truck (%)	20 (16.9)	10 (16.1)	10 (17.9)	11 (18.3)	9 (15.5)
Own motorcycle/scooter (%)	7 (5.9)	1 (1.6)	6 (10.7)	7 (11.7)*	0*
Own bicycle (%)	16 (13.6)	11 (17.7)	5 (8.93)	11 (18.3)	5 (8.6)
Public/shared vehicle (%)	97 (82.2)	52 (83.9)	45 (80.4)	47 (78.3)	50 (86.2)
Walking (%)	4 (3.4)	0*	4 (7.1)*	4 (6.7)	0
**Caregivers$^{\beta}$ – breakdown of**					

**“other” family members**					
Grandmother (%)	16 (13.6)	9 (14.5)	7 (12.5)	7 (11.7)	9 (15.5)
Grandfather (%)	9 (7.6)	5 (8.1)	4 (7.1)	7 (11.7)	2 (3.5)
Aunt (%)	5 (4.2)	2 (3.2)	3 (5.4)	3 (5)	2 (3.5)
Uncle (%)	0	0	0	0	0
**Parental characteristics**					
**Father education**	**n=117**	N=61	N=56	N=60	N=57
None (%)	41 (35%)	26 (42.6%)	15 (26.8%)	20 (33.3%)	21 (36.8%)
Madrassa (%)	13 (11.1%)	8 (13.1%)	5 (8.9%)	4 (6.7%)	9 (15.8%)
Primary (%)	20 (17.1%)	11 (18%)	9 (16.1%)	12 (20%)	8 (14%)
Secondary (%)	22 (18.8%)	4 (6.6%)	18 (32.14%)	12 (20%)	10 (17.5%)
Higher than secondary (%)	21 (17.9%)	12 (19.7%)	9 (16.1%)	12 (20%)	9 (15.8%)
**Mother education**	**N=118**	N=62	N=56	N=60	N=58
None (%)	80 67.8%)	44 (71%)	36 (64.3%)	42 (70%)	38 (65.5%)
Madrassa (%)	19 (16.1%)	8 (12.9%)	11 (19.6%)	6 (10%)	13 (22.4%)
Primary (%)	6 (5.1%)	3 (4.8%)	3 (5.4%)	3 (5%)	3 (5.2%)
Higher than secondary (%)	3 (2.5%)	2 (3.2%)	1 (1.8%)	6 (10%)	4 (6.9%)
**Child characteristics**					
**Chronic health conditions**	N=128	N=63	N=65	N=65	N=63
Cerebral palsy	28 (21.9)	10 (16%)	18 (28%)	19 (29.2%)	9 (14.3%)
Spina bifida	11 (8.6)	5 (7.9%)	6 (9.2%)	7 (10.8%)	4 (6.4%)
Congenital limb defect	45 (35.2)	29 (46%)	16 (24.6%)	24 (36.9%)	21 (33.3%)
Intellectual disability	45 (35.2)	21 (33.3%)	24 (36.9%)	22 (33.9%)	23 (36.5%)
Epilepsy/seizures	5 (3.9)	1 (1.6%)	4 (6.2%)	2 (3.1%)	3 (4.8%)
Others[χ]	45 (35.2)	18 (28.6%)	27 (41.5%)	22 (33.9%)	23 (36.5%)
Number with >1 health condition	48 (37.5)	21 (33.3)	27 (41.5)	29 (44.6)	19 (30.2)
Unwell in the past 2 months	47 (36.7)	19 (30.2)	28 (43.1)	34 (52.3)	13 (20.6)

Notes:

*P value <0.05, using t-test for continuous variables and $\chi 2$ or Fisher’s exact test as appropriate for binary variables.

[α] One rural family lived in a tent.

[β] People responsible for caring for index child. Multiple caregivers could be selected.

[χ] Free text box. Most reported conditions were: paralysis (N=14), communication difficulties (N=12) and either vision or hearing problems (5 & 6 respectively).

Table 2: Number (and percent) of functional difficulties[1] in participating children aged 2-4 years old

	**Total**	**Urban**	**Rural**	**Cohort 1**	**Cohort 2**
**Functional difficulty**	N=29	N=16	N=13	N=13	N=16
Seeing	0	0	0	0	0
Hearing	1 (3.4)	1 (6.3)	0	1 (7.7)	0
Walking	19 (65.5)	12 (75)*	7 (53.9)*	10 (76.9)	9 (56.3)
Fine motor	12 (41.4)	6 (37.5)*	6 (46.2)*	5 (38.5)	7 (43.8)
Communication	15 (51.7)	5 (31.3)	10 (76.9)	8 (61.5)	7 (43.8)
Learning	15 (51.7)	5 (31.3)	10 (76.9)	7 (53.9)	8 (50)
Playing	14 (48.3)	7 (43.8)	7 (53.9)	8 (61.5)	6 (37.5)
Controlling behaviour	7 (24.1)	4 (25)	3 (23.1)	4 (30.8)	3 (18.8)
Total functional difficulty	24 (82.8)	13 (81.3)	11 (84.6)	12 (92.3)	12 (75)

[1] measured using the Washington Group/UNICEF Child Functioning Module

* P value <0.05, using χ2 or Fisher's exact test as appropriate for binary variables.

Table 3: Number (and percent) of functional difficulties[1] in participating children aged 5-12 years old

	**Total**	**Urban**	**Rural**	**Cohort 1**	**Cohort 2**
**Functional difficulty**	N=99	N=47	N=52	N=52	N=47
Seeing	7 (7.1)	3 (6.4)	4 (7.7)	4 (7.7)	3 (6.4)
Hearing	7 (7.1)	2 (4.3)	5 (9.6)	3 (5.8)	4 (8.5)
Walking	67 (69.1)	35 (77.8)	32 (61.5)	34 (68)	33 (70.2)
Self-care	50 (50.5)	28 (59.6)	22 (42.3)	25 (48.1)	25 (53.2)
Communication	54 (54.5)	27 (57.5)	27 (51.9)	28 (53.9)	26 (55.3)
Learning	55 (55.6)	24 (51.1)	31 (59.6)	26 (50)	29 (61.7)
Remembering	53 (53.5)	27 (57.5)	26 (50)	23 (44.2)	30 (63.8)
Concentrating	34 (34.3)	18 (38.3)	16 (30.7)	16 (30.8)	18 (38.3)
Accepting change	44 (44.4)	19 (40.4)	25 (48.1)	22 (42.3)	22 (46.8)
Controlling behaviour	49 (49.5)	21 (44.7)	28 (53.9)	25 (48.1)	24 (51.1)
Making friends	43 (43.4)	19 (40.4)	24 (46.2)	24 (46.2)	19 (40.4)
Anxiety	56 (56.6)	22 (46.8)	34 (65.4)	36 (69.2)*	20 (42.6)*
Depression	57 (57.6)	25 (53.2)	32 (61.5)	39 (75)*	18 (38.3)*
Total functional difficulty	98 (99)	46 (97.9)	52 (100)	52 (100)	46 (97.9)

[1] measured using the Washington Group/UNICEF Child Functioning Module

* P value <0.05, using χ2 or Fisher's exact test as appropriate for binary variables.

Table 4: Number (and percent) of children aged 2-12 who use assistive devices

	**Total**	**Urban**	**Rural**	**Cohort 1**	**Cohort 2**
	128	63	65	65	63
**Domains**					
Wear glasses	9 (7)	7 (11.1)	2 (3.1)	5 (7.7)	4 (6.4)
Use hearing aid	2 (1.6)	0	2 (3.1)	0	2 (3.2)
Use equipment or receive assistance for walking	64 (50)	38 (60.3)	26 (40)	42 (64.6)	22 (34.9)
Difficulty seeing when wearing glasses	2 (22.2)	1 (14.3)	1 (50)	2 (40)	0
Difficulty hearing when using hearing aid	2 (100)	0	2 (100)	0	2 (100)
Difficulty walking with equipment/assistance	38 (59.4)	25 (65.8)	13 (50)	25 (59.5)	13 (59.1)

Table 5: Cohort comparison of PedsQL mean difference scores (standard deviation).

	**Cohort 1**	**Cohort 2**	**P**
**Total score**	**7.5 (19.5)**	**35.8 (17.1)**	**<0.0001**
Physical functioning	11.1 (23)	38.6 (23.7)	<0.0001
Emotional functioning	14.8 (29.8)	34.3 (22.6)	0.0001
Social functioning	6.8 (32.3)	40.2 (22.9)	<0.0001
Cognitive functioning	6.5 (28.2)	29.5 (23.4)	<0.0001
Communication	9.3 (31.9)	56.9 (26.6)	<0.0001
Worry	4.1 (20.1)	36.3 (21.5)	<0.0001
Daily activities	2.2 (30)	29.9 (24.2)	<0.0001
Family relationships	3.2 (27.7)	27 (23.2)	<0.0001
**Parent health related quality of life summary score**$^{\alpha}$	**10 (22.5)**	**35.6 (19.5)**	**<0.0001**
**Family functioning summary score**$^{\beta}$	**2.8 (24.9)**	**28.1 (19.1)**	**<0.0001**

Note: Higher scores indicate better functioning.

$^{\alpha}$Composite score including Physical, Emotional, Social, and Cognitive Functioning scales
$^{\beta}$Composite score including Daily Activities and Family Relationships

Table 6: PEDsQL scores by ethnicity. Mean (SD).

Ethnicity	Baseline	Endline
Pashtun	24.2 (12.5)*	52.4 (18.1)
Hazara	31.2 (4.2)*	50.3 (14.8)
Other Persian (Tajik & Uzbek)	38.1 (16)*	50.5 (15.9)

*Statistically significant difference determined by One-Way ANOVA. $F(2,115) = 9.91$, $p = 0.0001$. Posthoc analysis using Tukey's method showed that Pashtun was significantly higher than both Hazara and Other Persian. ($p < .05$)

Annex 1: Situational Analysis

From August 2020 to December 2020 we conducted a review of the burden of disability among children and young people in Afghanistan, as well as the services and support available for these children and their families to assess the potential role of caregiver support groups in this setting (HREC # 2019.023). This situational analysis included (1) a review of the available literature on disability in LMIC and humanitarian settings and evidence for utility of caregiver support groups, and (2) semi-structured qualitative interviews with ten families caring for children living with disability in Kabul, Afghanistan and eight professional representatives of organisations working with people living with disability, including:

- World Health Organisation, Kabul office
- Handicap International, Kabul office
- Afghanistan Ministry of Public Health
- Save the Children, Kabul office
- UNICEF, Kabul office
- Indra Gandi National Children's Hospital
- ICRC, Kabul office
- Afghan Red Crescent Society

Annex 2: Program Adaptation and Development

The development of a context-appropriate evidence-informed support group program was an iterative process involving (1) review of existing programs and models of care, (2) situational analysis, and (3) a series of team meetings with input from paediatricians, disability experts, local implementers, and the Ministry of Health.

Local Advisory board

A local advisory board was established to provide project oversight, technical guidance and facilitate future scale up. Members included representatives from:

- Afghanistan Ministry of Public Health
- Afghanistan disability directorate
- Afghanistan surveillance department
- WHO
- Save the Children
- UNICEF
- Humanity and Inclusion
- Afghan Red Cross
- Indra Ghandi hospital
- Relief Humanitarian Development Organisation

Program Development Workshop

In December 2019 the Mighty Children project team met in Tashkent, Uzbekistan, to review the formative research and discuss program development for the Afghan context. Representatives from the MOPH also attended this meeting to provide input into developing the program and to support coordinated implementation. During this meeting, attendees were provided with a summary of the situational analysis and were provided with alternative models of care from which to develop the program.

Following the program workshop in December 2019, two members of the project team (paediatrician and clinical psychologist) drafted a structured program using *Getting to Know Cerebral Palsy* (now known as Ubuntu)(9) as a framework, given its promising results for caregiver wellbeing in LMICs(10-14).

Annex 3: Program description

The *Mighty Children Caregiver Support Program* (the program) is a psychoeducational support group program for caregivers of children living with chronic health conditions or disability that aims to improve child and family wellbeing, improve understanding of disability, develop care-giving skills, and build social/peer-support networks. An outline of the modules including topics and ACT exercises covered is below:

**Module number and title**	**Topics covered**	**ACT skills covered**
1: Introduction	Overview and group rules<br>Meet other caregivers<br>Understand disability	Open up: understanding emotions<br>Being present: breathing
2: My Mighty Child	Growth and development<br>Identified strengths and challenges	Open up: understanding emotions<br>Being present: body awareness
3: Move and communicate	Strategies to help children move<br>Strategies to help children communicate	Open up: sitting with difficult emotions<br>Open up: Labelling emotions
Session 4: Eating and everyday activities	Healthy eating<br>Safe feeding strategies<br>Strategies to help children develop new skills through everyday activities	Being present: mindful moments
Session 5: Our Mighty family	Strategies to support families' physical and mental wellbeing<br>Understand how children's emotions may affect their behaviour	Being present: grounding<br>Values: value compass<br>Open up: thoughts are just thoughts
Session 6: Looking after ourselves	Understand how caregivers' emotional wellbeing impacts on our child<br>Strategies to help cope with difficult situations<br>How play can be used to help children develop	Open up: linking events and emotions<br>Be present: self-compassion
Session 7: Our Mighty team	Understand children's rights in society<br>Identify ways we can work together to help children with disability	Be present: tea meditation
Session 8: Our Mighty Community	Get to know key members of family and community<br>Identify challenges and opportunities for community support	Be present: tea meditation
Session 9: Next steps	Program review<br>Decide on practical next steps	Review activities

Annex 4: Plain language statement

اومه ضمیمه : ساده بیانیه

ضمیمه هفتم: بیانیه ساده

**HREC Project Number:**<br>نمبر پروژه:<br>دپروژی نمره:	61117<br>۶۱۱۱۷		
**Short Name of Project:**<br>نام خلاصه پروژه<br>دپروژی لنډ نوم	Mighty Children program evaluation<br>پروگرام ارزیابی اطفال توانمند<br>دپیاوړو ماشومانو پروګرام		
**Principal Researcher:**<br>تحقیق کننده اصلی<br>اصلی تحقیق کونکی	Dr Hamish Graham, Chief investigator (Australia)<br>Dr Faiz Mohammed Atif, Project Lead (Afghanistan)<br>(داکتر همیش گراهام، سرمحقق (استرالیا<br>داکتر فیض محمد عاطف دپروژی رهبری کونکی، رهبری کننده پروژه		
**Version Number:**<br>نمبر نسخه<br>دنسخی نمره	1.2<br>۱.۲	**Version Date:**<br>تاریخ نسخه<br>دنسخی تاریخ	8/4/2020<br>۸/۴/۲۰۲۰

Dear Caregiver,

I would like to invite you to take part in the *Mighty Children* education and support program for caregivers of children living with a chronic health condition or disability. We are from an Afghan NGO called RHDO (Relief and Humanitarian Development Organization). *Mighty Children* is a new project that brings families with children with chronic health conditions or disability together for education and support. We are working with researchers in Australia (Murdoch Children's Research Institute) to implement and evaluate the program. We have been given funding from a Canadian non-profit organization (Grand Challenges Canada).

خوښ یو چه تاسو (دهغو ماشومانو پالونکوته چه مزمنی ناروغی اویا معلولیت لری) بلنه درکړوتر څو دهغو ماشومانو چه مزمنی ناروغی او یا معلولیت لری په تعلیمی او حمایوی پروګرام کی ګډون وکړی.

دبشردنجات اوپرمختیایی موسسی چه یوه افغانی موسسه ده ددی پروژی تطبق کونکی ده. دپیاوړوماشومانو پروژه یوه نوی پروژه ده غواړی هغه کورنی اوددوی ماشومان سره ټول کړی کوم چه پخپلو کورونوکی مزمنی ناروغی لرونکی ماشومان یا معلول ماشومان لری دتعلیم او حمایت لپاره په ګروپونوکی سره ټول کړی.

ددی پروژی تمویلونکی دکاناډا غیر انتفاعی موسسه ده.

مسرت دارم که شما(پرستاران اطفال با امراض مزمن ویا معلولیت) را دعوت می کنم تا اشتراک نماید در پروگرام تعلیمی وحمایوی اطفال توانمند که مشکلات مزمن صحی ومعلولیت دارند.

ما از موسسه افغانی که بنام موسسه بشردوستانه انکشافی نجات است نمایندگی می کنم که تطبق کننده این پروژه است. پروژه اطفال توانمند یک پروژه جدید است که جمع می کند فامیل های اطفال که امراض مزمن ویا معلولیت دارد برای تعلیم وحمایت.

ما کار می کنیم با محققین استرالیایی (انستیتیوت تحقیقاتی اطفال ماردوچ استرالیا) تا این پروگرام را تحقیق وارزیابی نمایم. بودجه این پروژه راموسسه غیر انتفاعی کانادا تمویل میکند.

The *Mighty Children* program aims to improve caregiver's confidence in caring for their child, and connect them with other caregivers of children with chronic health conditions or disability. This is the first time the program is being run in Afghanistan so we are doing research to test it.

دپیاوړوماشومانودپروګرام اهداف: دمعلولوماشومانودپالونکو اعتماد په نفس ښه کول، ددوی دماشومانوپه پالنه کې او ددوی داړیکوټینګول دنورو هغوپالونکوسره چه دوی هم مزمن ناروغی لرونکی یا معلول ماشومان لری. دا پروژه دلمړی ځل لپاره په افغانستان کې تطبق کیږی. مونږ دا تحقیق امتحانوو.

اهداف پروگرام اطفال توانمند: بهبود دراعتماد به نفس مراقبت کننده اطفال که امراض مزمن یا معلولیت دارد بخاطر مراقبت از اطفال وایجاد روابط بین پرستاران اطفال که امراض مزمن صحی ویا معلولیت دارد. این اولین پروگرام است که در افغانستان تطبق می گردد وما این تحقیق را امتحان می کنیم.

The *Mighty Children* groups will meet together once every week for a total of 10 weeks, and will involve 8-10 caregivers and 2 facilitators. The meetings will last ~3 hours, including discussion, activities, learning, and snacks. By the end of the program we hope that you will have made new friends, become more confident in caring for your child, and better understand your child's condition. The *Mighty Children* program does not provide medications, medical supplies, or other material support. It does not replace medical or physical rehabilitation services.

دپیاوړوماشومانو ګروپونه په اونی یا هفته کې یو ځل خپل منځ کې ملاقات کوی او دلسو هفتو دپاره به دا پروګرام وی او ۸ څخه تر ۱۰ پوری د ماشومانو پالونکی او دوه لارښونکی به ګډون کوی. دا ملاقات به ددری ساعتونو دپاره دوام کوی. پدی ملاقات کی به بحثونه، فعالیتونه، زده کړی او مختصر خوراک شامل وی. دپروګرام په پای کې مونږهیله لرو دا ګروپونه نوی ملګری ولری، زیاد اعتماد په ځان ولری تر څو دمعلولوماشومانوښه پالنه وکړي، اودخپل ماشوم په وضعیت ښه پوه شی. دپیاوړو ماشومانو پروګرام دوایی، طبی تجهیزات او نور حمایوی مواد نه تهیه کوی.

پروگرام اطفال توانمند هر هفته یک ملاقات باهم میداشته باشند واین پروگرام برای ۱۰ هفته دوام می کند ودرین ۸ تا ۱۰ پرستار ودو نفر راهنما اشتراک می کند. این ملاقات برای سه ساعت دوام می کند. این ملاقات ها شامل بحث ها، فعالیت ها، اموزش و خوراک مختصر می باشد. دراخرپروگرام امیدوارم که همه گروپ ها با هم دوست ودوستان جدید پیدا کند. اعتماد به نفس زیاد شود تا از اطفال معلول خوب پرستاری کند، ووضعیت اطفال خود خوب بفهمد. پروگرام اطفال توانمند دوا، تجهیزات طبی مواد حمایوی را تهیه نمی کند.

During the *Mighty Children* program we will be collecting information to help us evaluate the program and understand child disability. We will ask your permission if we want to use any material from you (e.g. photos, responses, feedback, etc.). We hope that this research will help other groups develop better ways to support children and families living with chronic health conditions or disability.

دپیاوړوماشومانو دپروګرام په جریان کې مونږمعلومات جمع کووتر څو مونږ سره په ارزیابی کی مرسته وکړی اود معلولو ماشومانو باره کې زیاته پوهه تر لاسه کړو. مونږ به له تاسی اجازه واخلو(که چیری مونږ تاسو تصویر اخیست، یا ځواب اویا باز دهی او داسی نور). مونږ هیله لرو چه دا تحقیق مرسته وکړی له نوروګروپونوسره تر څوښولارو ته انکشاف ورکړی او دمعلولو اومزمنو ناروغیو ماشومانو اوددوی کورنیو حمایت وکړی .

درجریان پروگرام اطفال توانمند ما معلومات را جمع می کنیم تا در ارزیابی پروگرام و فهمیدن اطفال معلول کمک کند.ما به اجازه شما خواهد مواد مثلی عکس،جواب، وباز دهی وغیره را اخذ نمایم. ما امید وارهستم که این تحقیق کمک خواهد کرد با دیگر گروپ ها تا انکشاف دهد راه های خوب را به خاطر حمایت اطفال معلول ویا اطفال که امراض مزمن دارد وحمایت فامیل های ان.

If you have any questions about the project, you can contact Dr Hamish Graham on +61 3 9345 6262 (hamish.graham@rch.org.au), or Dr Faiz on 0799687925 (drfaiz.rhdo@gmail.com).

که چیری تاسو هر سوال دپروژی باره کی لری نو تاسو کولای شی چه له داکتر همیش گراهام څخه پدی شمیره

+61393456262 (Hamish.graham@rch.org.au) اړیکه ونیسی . اویا داکتر فیض محمد عاطف سره پدی شمیره

۰۷۹۹۶۸۷۹۲۵ اړیکه ونیسی

Drfaiz.rhdo@gmail.com

اگر شما هرسوال دارند در باره پروژه شما می تواند با این شماره با داکتر همیش گراهام وداکتر فیض محمد عاطف تماس بگیرند

+61 3 9345 6262 (hamish.graham@rch.org.au), or Dr Faiz on 0799687925 (drfaiz.rhdo@gmail.com).

مننه ستاسو دوخت څخه

تشکر از وقت شما

Thank you very much for your time.

Dr Hamish Graham, Senior Research Fellow, MCRI, Melbourne, Australia
Dr Faiz Mohammad Atif, General Director, RHDO, Kabul, Afghanistan

You can contact the Director of Research Ethics & Governance at The Royal Children's Hospital Melbourne on +61 3 9345 5044 if you:

- have any concerns or complaints about the project
- are worried about your rights as a research participant
- would like to speak to someone independent of the project.

تاسو کولی شئ د رائل چلډرن هسپتال میلبورن کې د څېړنو د اخالقي او حکومتدارۍ رییس سره په 61+ 3 9345 5044 اړیکه ونیسئ که تاسو:

- د پروژې په اړه کومه اندیښنه یا شکایت لري
- د تحقیق برخه اخیستونکي په توګه ستاسو د حقونو په اړه اندیښنه لرئ
- غواړي له چا سره خبرې وکړي چې د پروژې خپلواک نه وي

در صورت تمایل می توانید با مدیر اخلاق تحقیق و حاکمیت در بیمارستان کودکان رویال ملبورن با شماره 61+ 3 9345 5044 تماس بگیرید:

- هر گونه نگرانی یا شکایتی در مورد پروژه دارید
- نگران حقوق شما به عنوان یک شرکت کننده تحقیق هستید
- دوست دارم با شخصی مستقل از پروژه صحبت کند.

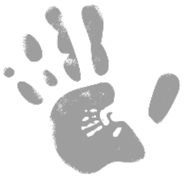

Information for Caregivers and Facilitators

معلومات برای سرپرست و راهنما

د پالونکی او لارشوونکی لپاره معلومات

What is the *Mighty Children* program?

د اتلو مشومانو پروگرام حه ده؟

پروگرام اطفال مستمند چیست؟

Mighty Children is an educational support group program for caregivers of children with physical disability (e.g. cerebral palsy, spina bifida, and other physical conditions). It aims to help caregivers understand their child, and to build support networks that will help children reach their full potential.

اتل ماشومان هغه تعلیمی تولنه ده چی هغو پالونکو لپاره ده چی د جسمی معلول لکه سریرال پولزی, سپاینا فیدا او یا نور و باندی اخته ماشومانو ته مرسته کوی داسی اسانتیاوی او اریکی جوری کری تر دا ماشومان د خپل زوند شه ارمانو ته بریالی کری.

اطفال مستمند یک گروپ تعلیمی برای سرپرستان اطفال معلول جسمی چون سریرال پولزی' سپاینا بای فیدا و دیگر حالات معلولی جسمی بوده تا رشته امدادی را تعمیر ساخته و چنین اطفال را به ارزوهای زندگی شان نایل سازند

Why should I join a *Mighty Children* group?

چرا با کروپ اطفال مستعد یکجا شویم؟

ولی دی اتلو ماشومانو کروپ سره یو جای شو؟

The *Mighty Children* program is your opportunity to:

- Gain skills and confidence in caring for your child;
- Learn strategies to care for you and your family's emotional and mental health;
- Meet and learn from other parents/caregivers who also have children living with disability;
- Share your knowledge and skills to teach and support other parents/caregivers;
- Join other caregivers/parents to advocate for better health services.

د اتلو ماشومانو پروگرام تاسو ته لاندی زمینی برابروی:

- تاسو ته زده کره او اطمنان درکوی تر د ماشوم لپار شه خدمت وکری
- دزان او کورنی پالنی د زده کړی لیاری او په خاصه دول د فکری او روحی روغتیا
- معرفی او زدکړه له نورو والدینو /پالونکونه چی معیوب ماشومان لری
- خپله پوهه او تجربی شریک او نور والدینو/پالونکو ته شوونه او کومک
- له نورو والدینو/پالونکو یو زای کولای شی چی دی نور شه صحی خدمتونه لاس ته راوړی

پروگرام اطفال مستعد برای شما زمینه سازی ذیل براورده می سازد:

- برای شما امکانات اموزشی و اطمنان فراهم می سازد تا از طفل پرستاری نمایید
- اموزش طروق بهداشتی از خود و فامیل خویش بخصوص صحت فکری و روحی
- معرفی و اموزش از والدین/پرستاران دیگر که اطفال معیوب دارااند
- در میان گذاشتن دانش و تجارب و تدریس و تقویه والدین /پرستاران دیگر
- با همکاری دیگر والدین/پرستاران میتوان خدمات صحی بهتر بدست اورید

Why is this for parents/caregivers?

چرا این برای والدین / سرپرستان است؟

ولې دا د والدینو / پاملرنې کونکو لپاره دی؟

The biggest influence on a child's life is their family, particularly their primary caregiver (e.g. mother). Caring for children with chronic health conditions can be stressful and difficult. However, supporting parents/caregivers can improve the health and wellbeing of children and families.

کورنۍ یوه اصلی اغیزه د ماشوم په ژوند باندی لری خصوصا لمړنی پالونکی (د مثال په توگه مور). د هغو ماشومانو پالنه چی په مزمن او غیر صحی حالت اخته دی شاید روحی او نوری ستونزی رامخته کړی خوله والدینو /پالونکو سره مناسبه مرستی, کولای شی چی د ماشومانو او د هغوی دکورنیو په سلامتیاکی شه بدلون راولی.

فامیل تاثیرعمده را بالای زندگی طفل دارد, بخصوص پرستار اولیه وی (بطور مثال مادر). پرستاری ازاطفال که دارای حالات غیر صحی مزمن هستندممکن باعث فشار روحی و مشکلات شود. اما مساعدت مناسب به والدین/پرستاران میتواند حالت صحی و سلامتی اطفال و فامیلی انها را بهبود بخشد.

Why focus on children with disability?

ولی معلولو ماشومانو ته خاصه پاملرنه وشی؟

چرا به اطفال معلول توجه خاص شود؟

Children living with chronic health conditions and disability are at risk of poor health and social outcomes – including illness, death, educational disadvantage, stigma, and lack of job opportunities. However, these children can be 'mighty' and successful if they are given the opportunity.

هغه ماشومان چی له مزمنو صحی تکلیفو او معیوبیت سره ژوند کوی, دوی ته ددی خطرات نور هم زیات دی چی خوار صحی حالت او بد تولنیز ژوند ته ولویزی _ لکه نارغی, مرگ, د تعلیم وروستوالی, شرمندگی, او د دندو نه محرومیت. اما دا ماشومان کولای شی چی "اتلان" وی او بریالی شی که چیرته. دوی ته شرایط برابر کړی شی.

کودکان که با حلالت مزمن صحی و معیوبیت مبتلا زندگی می کنند, شاید مواجه امراض دیگر و درگیر انزجاه اجتماعی شوند چون تکلیف صحی, فوت, عقب ماندن از تعلیم, شرمندگی, و محرومیت از داشتن کار و وظیفه. اما چنین اطفال میتوان"قدرتمند" باشند به شرط اینکه برای شان شرایط مناسب تهیه گردد.

Who can attend the group?

کوم کسلن کولی شی چی په دی گروپ کی ونده واخلی؟

چی کسانی میتوان که در این گروپ اشتراک ورزد؟

The groups are intended primarily for female primary caregivers (e.g. mother, sister, grandmother, aunt). However, we encourage you to bring your child and another family member to help look after the child while you participate. We have one session dedicated to involving a male caregiver (e.g. father, brother, grandfather, uncle) and other family members.

د گروپ جوړولو اصلی هدف دی هغو شحو له پاره ده چی دی ماشوم پالنه یی په غاړه ده (مور, خور, انا, خاله او عمه). اما موز د کور نور و غړو ته هم تشویق ورکوو چی راشی او د ماشوم پالنه وکړی تر حو تاسی په صنف کی برخه واخلی.

هدف اولیه تشکیل گروپها برای پرستاران نسوان (مادر, خواهر, مادرکلان, خاله, عمه) میباشد. اما ما اوردن طفل تان و یا اعضای دیگر فامیل تانرا نیز تشویق می داریم تا از طفل تان در هنگام اشتراک شما در صنف پرستاری نمایند. ما یک برای. عضو مذکر فامیل پرستار (پدر, برادر, پدر کلان و کاکا) نیز در نظر داریم.

What happens in the *Mighty Children* groups?

در گروه های مستعد چه اتفاق می افتد؟

د اتلو ماشومانو په ګروپونو کې څه پیښیږي؟

The *Mighty Children* program runs for 8-10 sessions. We suggest that you meet every week, and allow at least 2 hours per session. The groups consist of activities, discussion, and opportunities to share and learn from each other. Each group can decide how to run the sessions and what rules to adopt. The groups should be interactive and fun for caregivers and children.

پروگرام اطفال مستعد برای مدت 8-10 هفته در بر می گیرد. ما توقع داریم تا شما هفته یک بار برای دو ساعت با هم ملاقات داشته باشید. گروپ ها بالای محتویات چون فعالیت های شان , مباحثه,شریک ساختن نظریات و تجارب شان و اموختن از یکدیگر استفاده نمایند. هر گروپ اختیار دارد که چگونه مجالس خویش را به پیش ببرد و چگونه راهنمود را وضع کند. گروپ ها باهم فعلایت مشترک داشته باشند و برای پرستاران و اطفال دلچسپ باشد.

د غالب ماشوم پروګرام د 8-10 غونډو لپاره پرمخ ځي. موږ وړاندیز کوو چې تاسو هره اونۍ وګورئ ، او په هره ناسته کې لږ تر لږه 2 ساعته اجازه ورکړئ. په ډلو کې فعالیتونه ، بحث او فرصتونه دي چې له یو بل څخه د شریکولو او زده کړې فرصتونه وي. هره ډله کولی شي پریکړه وکړي چې څنګه ناستې پرمخ وړي او کوم قانون باید پلي شي. ډلې باید د پاملرنې کونکو او ماشومانو لپاره متقابل عمل او ساتیري وي.

Who will facilitate the groups?

چی کسی این گروپ ها را راهنما یی می کند؟

سوک دا ګروپونه تیاروی؟

Every group will be guided by a trained facilitator, but they are not meant to be the expert. Facilitators are here to help the groups run smoothly and create a safe place for you to share your experiences, learn and practice new skills. By the end of the program, caregivers should be able to continue meeting and supporting each other without a facilitator.

هر گروه توسط یک مجری آموزش دیده راهنمایی می شود ، اما منظور این نیست که وی متخصص باشد. تسهیلگرها در اینجا هستند تا به گروهها کمک کنند تا بتوانند مجالس به اسانی پیش برودد و یک فضا امن را برای شما ایجاد کنند تا تجربیات خود را به یکدیگر شریک سازید ، بیاموزید و مهارت های جدید بدست اورید. تا پایان برنامه ، مراقبان باید بدون تسهیلگر بتوانند به کومک یکدیگر به جلسات ادامه دهند.

هر گروپ به دی یوه ماهر لارکس له خوا لارشوونه کیزی اما دا په دی معنی نده چی نوموری به متخصص وی. لارشوونکی گروپ ته دی کومک لپاره دی او د کروپ کرنی به په اسانی سره.مخته بوزی او یوه دادینه زمینه برابره کړی تر سو تاسی خپلی تجربی له یو بل سره گدی کړی او نوی زدهکړې او مهارتونه لاس ته راوړی. دزده کړی. د دی پروگرام په پای کی به ، ساتوونکی به وکولای شی چی لارشوونکی نه پرته دیوبل په مرسته ناستې ته دوام ورکړي.

What will I need to do?

چه کاری باید انجام دهم؟

زه به څه وکړم؟

You just need to attend the meetings and participate in the activities. The *Mighty Children* program uses activities, games, pictures, and discussion to encourage active learning and group problem solving. By participating in the activities and discussions, you will benefit and you will help others. Sometimes you will be given simple tasks to do at home before the next session. Participating in the program should never be a burden on you or give you more work.

شما فقط باید در جلسات حاضر شوید و در فعالیت ها شرکت کنید. برنامه اطفال مستعد از فعالیت ها ، بازی ها ، تصاویر و بحث و گفتگو برای تشویق یادگیری فعال و حل مسئله گروهی استفاده می کند. با شرکت در فعالیت ها و مباحث ، شما سود خواهید برد و به دیگران کمک خواهید کرد. بعضی اوقات کارهای ساده ای برای انجام کار در خانه به شما قبل از جلسه بعدی داده می شود. شرکت در این برنامه هرگز نباید برای شما سنگین باشد و یا به شما کار بیشتری بدهد.

تاسو هرومرو په غونډو کې زان حاضر کړی او په فعالیتونو کې پې برخه واخلئ. د اتلو ماشومانو پروگرام د فعالیتو, لوبو ، عکسونه او بحثونوچخه دزدکړو لپاره کار اخلي. په فعالیتونو او بحثونو کې برخه اخیستنې سره ، تاسو به ګټه پورته کړئ او تاسو به له نورو سره مرسته وکړئ. خینې وختونه به تاسو ته د راتلونکې غونډې څخه دمخه په کور کې ترسره کولو لپاره ساده وظیفې درکړل شي. په برنامه کې برخه اخیستل باید هیڅکله تاسو باندې بار زیات نکړی او یا تاسو ته نورزیات کار درکړي.

Will we receive rehabilitation, medications, or medical equipment?

آیا صحت، داروها یا تجهیزات پزشکی دریافت خواهیم کرد؟

ایا موږ به بیا رغونه ، درمل ، یا طبي تجهیزات ترلاسه کړو؟

No. The *Mighty Children* program is intended to complement other therapies and medical treatment – not to replace them. These groups will help you develop skills and confidence in caring for your child, and introduce you to other families who also have children with physical disability. You may also be introduced to local health services. However, our program will not provide health services, medications, or medical equipment for your child.

نه. برنامه کودکان توانا برای همراهی و تکمیل شدن روش های درمانی و معالجه پزشکی موجوده است- نه جایگزینی آنهاو. این گروه ها به شما در ایجاد مهارت و اعتماد به نفس در مراقبت از فرزندتان کمک می کنند و شما را با خانواده های دیگری که دارای فرزندانی با ناتوانی جسمی هستند,معرفی می کنند. همچنین ممکن است شما با خدمات درمانی محلی آشنا شوید. با این وجود ، برنامه ما خدمات درمانی ، داروها یا تجهیزات پزشکی را برای فرزند شما ارائه نمی دهد.

نه. د اتلو ماشومانو پروګرام د نورو درملنو او طبي درملنې بشپړولو لپاره دی - نه د دوی ځای په ځای کولو لپاره. دا ډلې به ستاسو سره ستاسو د ماشوم پاملرنې ساتلو کې د مهارتونو او باور رامینځته کولو کې مرسته وکړي ، او تاسو نورو کورنیو ته به معرفي کړئ څوک چې فزیکي معلولیت لرونکي ماشومان هم لري. تاسو ممکن محلي روغتیا خدماتو ته هم معرفي شئ. په هرصورت ، زموږ برنامه به ستاسو ماشوم لپاره روغتیایی خدمات ، درمل ، یا طبي تجهیزات چمتو نکړي.

Are there any risks to me?

آیا خطری برای من وجود دارد؟

ایا ما ته کوم خطر شتون لري؟

The *Mighty Children* program is designed to support you and build your confidence. However, it may give you some difficult emotions. Some sessions we talk about sensitive issues, such as child abuse and violence. Caregivers may tell the group about negative experiences, violence, or stigma. We will always care about you, and what you are experiencing, even if we cannot change it. If we believe any child is at risk of injury or other maltreatment, our staff will inform the director so that we can help you. If you wish, we can refer you to health or support services.

- Violence against children or their mothers is not acceptable.
- Children with disability, and their caregivers, have a right to be protected.
- Everybody is responsible for keeping children safe and protecting them from violence and abuse.

برنامه اطفال مستعد برای حمایت از شما و ایجاد اعتماد به نفس طراحی شده است. با این وجود ، ممکن است احساسات سختی را به شما منتقل کند. در بعضی از جلسات ما درباره موضوعات حساس مانند کودک آزاری و خشونت صحبت می کنیم. مراقبان ممکن است تجربیات منفی ، خشونت یا ننگ را به گروه بگویند. ما همیشه به شما اهمیت خواهیم داد ، و آنچه شما تجربه می کنید ، حتی

اگر نمی توانیم آن را تغییر دهیم. اگر ما معتقدیم که هر کودکی در معرض آسیب و یا سوء رفتارهای دیگر است ، کارکنان ما به مدیر اطلاع می دهند تا ما به شما کمک کنیم. در صورت تمایل ، ما می توانیم شما را به خدمات درمانی یا خدمات پشتیبانی ارجاع دهیم.

- خشونت علیه کودکان یا مادران آنها قابل قبول نیست.
- کودکان دارای معلولیت و مراقبین آنها حق دارند از آنها محافظت شوند.
- همه وظیفه دارند کودکان را در امان نگه دارند و از خشونت و سوءاستفاده محافظت کنند.

د اتلو ماشومانو پروګرام ستاسو د ملاتړ او ستاسو باور رامینځته کولو لپاره جوړ شوی. په هرصورت ، دا سی امکان لری چی تاسو ته یو څه مشکل احساسات درکړي. ځيني غونډو کی موز د حساسو مسلو په اړه خبرې کوو ، لکه د ماشومانو سخه ناوړه ګټه اخيستنه او تاوتريخوالی. پاملرنې کونکي ممکن دلې ته د منفي تجربو ، تاوتريخوالي یا بدنامي په اړه ووایی. موږ به تل ستاسو په اړه پاملرنه وکړو ، او هغه څه چې تاسو يې تجربه کوئ ، حتی که موږ يې نشو بدلولی. که موږ باور ولرو چې کوم ماشوم د ټپي کيدو یا نور بد چلند سره مخامخ دی ، نو زموږ کارمندان به رییس ته خبر ورکړي ترڅو موږ وکولی شو ستاسو سره مرسته وکړو. که تاسو وغواړئ ، موږ کولی شو تاسو روغتيا يا ملاتړ خدماتو ته راجع کړو.

- د ماشومانو او میندو په وړاندې تاوتريخوالی د منلو وړ ندي.
- د معلولیت لرونکي ماشومان ، او د دوی پاملرنې کونکي حق لري چې خوندي وساتل شي.
- تول د ماشومانو د خوندي ساتلو او د تاوتريخوالي او ناوړه چلند څخه د هغوی د ساتنې لپاره مسؤل دی.

What will happen when the program ends?

دراخیربرنامه چه اتفاق می افتد؟

ددی پروګرام په پای کی به څه پیشیزی؟

When the program ends the facilitators will stop organising the weekly meetings, but that does not mean you should stop meeting. These are your groups and we hope that you will continue meeting and supporting each other, even after the formal program is finished.

با پایان یافتن برنامه ، راهنمای جلسات هفتگی را انتظام نمی دهد، اما این بدین معنی نیست که شما باید جلسات را متوقف کنید. این گروپ ها از شماست و ما امیدواریم که شما بعد از اتمام برنامه رسمی. مجالس را دوام بدارید، و به حمایت از یکدیگر ادامه دهید.

کله چې برنامه پای ته ورسيږي لارشوونکی به د اونۍ غونډو تنظیمول ودروي ، مګردا پدې معنی ندي چې تاسو باید ناسته ودروئ. دا ستاسو ډلې دي ، او موږ تمه لرو چې تاسو به د يو بل سره ناسته او ملاتړ ته دوام ورکړئ ، حتی د رسمي برنامې پای ته ورسيزی.

More about the *Mighty Children* program

The *Mighty Children* program was developed by a group of healthcare workers who wanted to provide better support to children, young people, and families living with chronic health conditions or disability in Afghanistan. With the support of the Islamic Republic of Afghanistan Ministry of Public Health (MOPH), we met with disability workers, and children and caregivers living with chronic health conditions and disability to understand what would work. We then created the *Mighty Children* program, using materials from other support groups (particularly *Getting to Know Cerebral Palsy/Juntos*[1] and *CLAN*[2]) and psychological therapies (particularly mindfulness and Acceptance and Commitment Therapy - ACT).

دپیاوړو ماشومانوباره کې نوراضافي معلومات:

دپیاوړو ماشومانوپروګرام ته دیوروغتیایي کارکونکوګروپ په واسطه انکشاف ورکړل شوی دغه روغتیایي ګروپ غواړي چه ماشومان، ځوانان او هغه کورنۍ چه دوی مزمن امراض لرونکي او یا معلول ماشومان لرې ښه حمایت کړي په افغانستان کې.

دافغانستان داسلامي جمهوریت او دعامې روغتیا دوزارت په مرسته مونږ وکولای شول چه د معلولوکارمندانو،ماشومانوددوی کورنۍ، پالونکوچه معلول ماشومان او یا مزمنې ناروغۍ لرونکي ماشومان لرې ملاقات وکړو او پوه شو چه څه کار ورته وکړو.

نو مونږ دپیاوړو ماشومانو ګروپ جوړکړ،مونږدنوروحمایوی ګروپونومواد استعمال کړل (په خاص ډول فلج عصبي) اوروانی تداوی (خاصتا -تمرکز حواس ، قبولي او تعهد تداوی).

معلومات اضافی در باره پروگرام اطفال توانمند:

پروگرام اطفال توانمند توسط یک گروپ کارکنان صحی انکشاف یافته ومی خواهد که به شکل بهتر اطفال،جوانان و فامیل های که

اطفال معلول ویا اطفال با امراض مزمن دارد حمایت کند در افغانستان. با همکاری جمهوری اسلامی افغانستان ووزارت صحت عامه ما ملاقات نمودیم کارمندان معلول، اطفال وپرستاران اطفال معلول واطفال که امراض مزمن دارد و بفهمیم که چه کار را برای شان اجرا کنیم. بعدا ما پروگرام اطفال توانمند را ایجاد کردیم واستعمال کردیم مواد را از گروپ های دیگر حمایوی (خاصتا از فلج عصبی) و تداوی روانشناسی (خاصتاتداوی تمرکز حواس،قبولی وتعهد).

Relief Humanitarian Development Organization (RHDO) is an Afghan non-governmental organisation working towards health and development. RHDO is the local lead for *Mighty Children* project. Contact: Dr Faiz Atif, drfaiz.rhdo@gmail.com.

دنجات بشردوستانه پرمختیایي موسسه یوه افغاني غیر دولتي موسسه ده چه دروغتیا اوپرمختیا دپاره کارکوی. دنجات بشردوستانه پرمختیایي موسسه دپیاوړو ماشومانو پروژه په محلي ډول تطبقوی. داړیکي مسول شخص: داکتر فیض محمد عاطف.

موسسه بشردوستانه انکشافی نجات یک افغانی وغیر دولتی میباشد که خدمات صحی وانکشافی را اجرا می کند وپروژه اطفال توانمند را در ساحه تطبق می کند.مسول تماس : داکتر فیض محممد عاطف

Murdoch Children's Research Institute (MCRI) is an Australian not-for-profit research institute dedicating to making discoveries that transform child health. MCRI researchers are leading the development and evaluation of the *Mighty Children* project. Contact: Dr Hamish Graham, hamish.graham@rch.org.au.

دمردوچ دماشومانو تحقیقاتي انستیتیوت یو استرالیایي غیرانتفاعي تحقیقاتي انستیتیوت ده او خپل اکتشافشات اهدا کوی دماشومانو دروغتیا دتغیر دپاره. دمردوچ دماشومانو تحقیقاتي انستیتیوت رهبری کوی دا انکشاف او ارزیابي د پیاوړو ماشومانو دپروژې. داړیکي مسول داکترهمیش ګراهام.

انستیتیوت تحقیقاتی مردوچ استرالیا که یک انستیتیوت تحقیقاتی غیرانتفاعی است که اکتشافات خود اهدا می کند بخاطر تغیر صحت اطفال. انستیتیوت تخقیقاتی مردوچ رهبری می کند انکشافات وارزیابی پروژه اطفال توانمند. مسول تماس داکتر همیش گراهام

Grand Challenges Canada is funding the *Mighty Children* project through the "Creating Hope in Conflict: a Humanitarian Grand Challenge" scheme, sponsored by U.S. Agency for International Development (USAID),

the UK Department for International Development (DFID), and the Ministry of Foreign Affairs of the Netherlands. Grant ID: R-HGC-POC-1904-24744.

ددی پروژې تمویلونکی د کانادا لویو چالشونوبودجه ده دپیاوړو ماشومانو دپروژې دپاره(تر څوهیله پیداکړی په تضاد او کشمکش کی) ددلویوبشری چالشونوطرحه او حمایت دامریکا بین المللی پرمختیایی مرستو، دبریتانیا شاهی حکومت دبین المللی پرمختیایی بنسټ او د نیدرلند بهرنیو چارووزارت لخوا شوی.

[1] https://www.ubuntu-hub.org/

[2] https://www.clanchildhealth.org/

Annex 5: Quantitative survey instruments

Family information – English

Use this form to collect baseline data from the caregiver. Some sections of this form will be used again at endline.

Caregiver name:	Date:
Child name:	Project officer(s):

Household questions

1) How many people usually sleep in your household?

   Adults: ______

   Children: ______

2) Do you own or rent your house?

   ☐ Own

   ☐ Rent

   ☐ Other (specify): ___________________________

3) What is your flooring material?

   ☐ Earthern (mud, hay, earth, sand, dung)

   ☐ Improved (cement, vinyl/plastic, tiles),

   ☐ Other (specify)

4) Do you have any of these items in your household:

   Radio: ☐ YES ☐ NO

   Television: ☐ YES ☐ NO

   Phone/mobile: ☐ YES ☐ NO

   Generator: ☐ YES ☐ NO

   Refrigerator: ☐ YES ☐ NO

5) What is your primary means of transport?

 Own Car/truck

 Own Motorcycle/scooter

 Own Bicycle

 Public/shared vehicle

 Other (specify): ______________________

Parent/Caregiver

6) Who is the child's primary caregiver within the household?

Mother	Grandfather	Sister
Father	Aunt	Brother
Grandmother	Uncle	Other

7) Are the parents living in the household?

Father: Yes No, separated No, deceased No, other (specify): ___________

Mother: Yes No, separated No, deceased No, other (specify):

8) What is the parent's ethnicity?

Father: Pashtun Tajik Hazara Uzbek Other (specify): ___________

Mother: Pashtun Tajik Hazara Uzbek Other (specify): ___________

9) What is the age of each parent?

Father: ____________

Mother: ___________

10) What is the highest level of parental education undertaken?

Father: None Madrassa Primary Secondary Higher than secondary

Mother: None Madrassa Primary Secondary Higher than secondary

11) How many children does the mother have?

Alive: _____

Deceased: _____

Age of youngest child: _____

Age of oldest child: ______

The next questions ask about difficulties PARENTS may have doing certain activities because of a health problem (UNICEF/Washington Group Short Set of Questions on Disability).

MOTHER

1) Do you have difficulty seeing, even if wearing glasses?

 No – no difficulty

 Yes – some difficulty

 Yes – a lot of difficulty

 Yes – cannot do at all

2) Do you have difficulty hearing, even if using a hearing aid?

 No – no difficulty

 Yes – some difficulty

 Yes – a lot of difficulty

 Yes – cannot do at all

3) Do you have difficulty walking or climbing steps?

 No – no difficulty

 Yes – some difficulty

 Yes – a lot of difficulty

 Yes – cannot do at all

4) Do you have difficulty remembering or concentrating?

 No – no difficulty

 Yes – some difficulty

 Yes – a lot of difficulty

 Yes – cannot do at all

5) Do you have difficulty with self-care (such as washing all over or dressing)?

 No – no difficulty

FATHER

1) Do you have difficulty seeing, even if wearing glasses?

 No – no difficulty

 Yes – some difficulty

 Yes – a lot of difficulty

 Yes – cannot do at all

2) Do you have difficulty hearing, even if using a hearing aid?

 No – no difficulty

 Yes – some difficulty

 Yes – a lot of difficulty

 Yes – cannot do at all

3) Do you have difficulty walking or climbing steps?

 No – no difficulty

 Yes – some difficulty

 Yes – a lot of difficulty

 Yes – cannot do at all

4) Do you have difficulty remembering or concentrating?

 No – no difficulty

 Yes – some difficulty

 Yes – a lot of difficulty

 Yes – cannot do at all

5) Do you have difficulty with self-care (such as washing all over or dressing)?

 No – no difficulty

□ Yes – some difficulty

□ Yes – a lot of difficulty

□ Yes – cannot do at all

6) Using your usual (customary) language, do you have difficulty communicating, for example understanding or being understood?

□ No – no difficulty

□ Yes – some difficulty

□ Yes – a lot of difficulty

□ Yes – cannot do at all

□ Yes – some difficulty

□ Yes – a lot of difficulty

□ Yes – cannot do at all

6) Using your usual (customary) language, do you have difficulty communicating, for example understanding or being understood?

□ No – no difficulty

□ Yes – some difficulty

□ Yes – a lot of difficulty

□ Yes – cannot do at all

Child with disability:

7) Age of child: _____ years

Date of birth (day/month/year): ___/___/________

8) What chronic health condition(s) does child have:

□ Cerebral palsy

□ Spina bifida

□ Congenital limb defect

□ Intellectual disability

□ Epilepsy/seizures

□ Other(s): ______________________

9) Has child received all immunisation (BCG, measles, DPT, polio):

□ YES

□ NO

□ Unsure

10) Has child been unwell in the past 2 months:

□ YES (specify: number of times; chief complaints): _____________________

□ NO

11) Have you sought medical care for child's acute illness in past 2 months:

 YES (specify: number of times; where and what treatment):______________________

 NO

12) Have you had any contact with health care providers in past 2 months:

 YES (specify: number of times; where and what treatment): ______________________

 NO

Complete the UNICEF/Washington Group Module on Child Functioning for the appropriate age:

- 2-4 years OR
- 5-17 years

**CHILD FUNCTIONING (AGE 2-4)**	**CF**	
**CF1**. I WOULD LIKE TO ASK YOU SOME QUESTIONS ABOUT DIFFICULTIES YOUR CHILD MAY HAVE.<br><br>DOES (*name*) WEAR GLASSES?	Yes 1<br>No 2	 2⇨CF3
**CF2**. WHEN WEARING HIS/HER GLASSES, DOES (*name*) HAVE DIFFICULTY SEEING?<br><br>WOULD YOU SAY (*name*) HAS: NO DIFFICULTY, SOME DIFFICULTY, A LOT OF DIFFICULTY OR CANNOT DO AT ALL?	No difficulty 1<br>Some difficulty 2<br>A lot of difficulty 3<br>Cannot do at all 4	1⇨CF4<br>2⇨CF4<br>3⇨CF4<br>4⇨CF4
**CF3**. DOES (*name*) HAVE DIFFICULTY SEEING?<br><br>WOULD YOU SAY (*name*) HAS: NO DIFFICULTY, SOME DIFFICULTY, A LOT OF DIFFICULTY OR CANNOT DO AT ALL?	No difficulty 1<br>Some difficulty 2<br>A lot of difficulty 3<br>Cannot do at all 4	
**CF4**. DOES (*name*) USE A HEARING AID?	Yes 1<br>No 2	 2⇨CF6

**CF5**. WHEN USING HIS/HER HEARING AID, DOES (*name*) HAVE DIFFICULTY HEARING SOUNDS LIKE PEOPLES' VOICES OR MUSIC?<br><br>WOULD YOU SAY (*name*) HAS: NO DIFFICULTY, SOME DIFFICULTY, A LOT OF DIFFICULTY OR CANNOT DO AT ALL?	No difficulty 1<br>Some difficulty 2<br>A lot of difficulty 3<br>Cannot do at all 4	1⇨CF7<br>2⇨CF7<br>3⇨CF7<br>4⇨CF7
**CF6**. DOES (*name*) HAVE DIFFICULTY HEARING SOUNDS LIKE PEOPLES' VOICES OR MUSIC?<br><br>WOULD YOU SAY (*name*) HAS: NO DIFFICULTY, SOME DIFFICULTY, A LOT OF DIFFICULTY OR CANNOT DO AT ALL?	No difficulty 1<br>Some difficulty 2<br>A lot of difficulty 3<br>Cannot do at all 4	
**CF7**. DOES (*name*) USE ANY EQUIPMENT OR RECEIVE ASSISTANCE FOR WALKING?	Yes 1<br>No 2	 2⇨CF10
**CF8**. WITHOUT HIS/HER EQUIPMENT OR ASSISTANCE, DOES (*name*) HAVE DIFFICULTY WALKING?<br><br>WOULD YOU SAY (*name*) HAS: SOME DIFFICULTY, A LOT OF DIFFICULTY OR CANNOT DO AT ALL?	Some difficulty 2<br>A lot of difficulty 3<br>Cannot do at all 4	
**CF9**. WITH HIS/HER EQUIPMENT OR ASSISTANCE, DOES (*name*) HAVE DIFFICULTY WALKING?<br><br>WOULD YOU SAY (*name*) HAS: NO DIFFICULTY, SOME DIFFICULTY, A LOT OF DIFFICULTY OR CANNOT DO AT ALL?	No difficulty 1<br>Some difficulty 2<br>A lot of difficulty 3<br>Cannot do at all 4	1⇨CF11<br>2⇨CF11<br>3⇨CF11<br>4⇨CF11
**CF10**. COMPARED WITH CHILDREN OF THE SAME AGE, DOES (*name*) HAVE DIFFICULTY WALKING?	No difficulty 1<br>Some difficulty 2	

WOULD YOU SAY (*name*) HAS: NO DIFFICULTY, SOME DIFFICULTY, A LOT OF DIFFICULTY OR CANNOT DO AT ALL?	A lot of difficulty 3<br>Cannot do at all 4	
**CF11**. COMPARED WITH CHILDREN OF THE SAME AGE, DOES (*name*) HAVE DIFFICULTY PICKING UP SMALL OBJECTS WITH HIS/HER HAND?<br><br>WOULD YOU SAY (*name*) HAS: NO DIFFICULTY, SOME DIFFICULTY, A LOT OF DIFFICULTY OR CANNOT DO AT ALL?	No difficulty 1<br>Some difficulty 2<br>A lot of difficulty 3<br>Cannot do at all 4	
**CF12**. DOES (*name*) HAVE DIFFICULTY UNDERSTANDING YOU?<br><br>WOULD YOU SAY (*name*) HAS: NO DIFFICULTY, SOME DIFFICULTY, A LOT OF DIFFICULTY OR CANNOT DO AT ALL?	No difficulty 1<br>Some difficulty 2<br>A lot of difficulty 3<br>Cannot do at all 4	
**CF13**. WHEN (*name*) SPEAKS, DO YOU HAVE DIFFICULTY UNDERSTANDING HIM/HER?<br><br>WOULD YOU SAY YOU HAVE: NO DIFFICULTY, SOME DIFFICULTY, A LOT OF DIFFICULTY OR CANNOT DO AT ALL?	No difficulty 1<br>Some difficulty 2<br>A lot of difficulty 3<br>Cannot do at all 4	
**CF14**. COMPARED WITH CHILDREN OF THE SAME AGE, DOES (*name*) HAVE DIFFICULTY LEARNING THINGS?<br><br>WOULD YOU SAY (*name*) HAS: NO DIFFICULTY, SOME DIFFICULTY, A LOT OF DIFFICULTY OR CANNOT DO AT ALL?	No difficulty 1<br>Some difficulty 2<br>A lot of difficulty 3<br>Cannot do at all 4	
**CF15**. COMPARED WITH CHILDREN OF THE SAME AGE, DOES (*name*) HAVE DIFFICULTY PLAYING?<br><br>WOULD YOU SAY (*name*) HAS: NO DIFFICULTY, SOME DIFFICULTY, A LOT OF DIFFICULTY OR CANNOT DO AT ALL?	No difficulty 1<br>Some difficulty 2<br>A lot of difficulty 3<br>Cannot do at all 4	

**CF16**. COMPARED WITH CHILDREN OF THE SAME AGE, HOW MUCH DOES (*name*) KICK, BITE OR HIT OTHER CHILDREN OR ADULTS?<br><br>WOULD YOU SAY: NOT AT ALL, THE SAME OR LESS, MORE OR A LOT MORE?	Not at all 1<br>The same or less 2<br>More 3<br>A lot more 4	

**CHILD FUNCTIONING (AGE 5-17)**		**CF**
**CF1**. I WOULD LIKE TO ASK YOU SOME QUESTIONS ABOUT DIFFICULTIES YOUR CHILD MAY HAVE.<br><br>DOES (*name*) WEAR GLASSES OR CONTACT LENSES?	Yes 1<br>No 2	 2⇨CF3
**CF2**. WHEN WEARING HIS/HER GLASSES OR CONTACT LENSES, DOES (*name*) HAVE DIFFICULTY SEEING?<br><br>WOULD YOU SAY (*name*) HAS: NO DIFFICULTY, SOME DIFFICULTY, A LOT OF DIFFICULTY OR CANNOT DO AT ALL?	No difficulty 1<br>Some difficulty 2<br>A lot of difficulty 3	1⇨CF4<br>2⇨CF4<br>3⇨CF4

	Cannot do at all 4	4⇨CF4
**CF3**. DOES (*name*) HAVE DIFFICULTY SEEING?<br><br>WOULD YOU SAY (*name*) HAS: NO DIFFICULTY, SOME DIFFICULTY, A LOT OF DIFFICULTY OR CANNOT DO AT ALL?	No difficulty 1<br>Some difficulty 2<br>A lot of difficulty 3<br>Cannot do at all 4	
**CF4**. DOES (*name*) USE A HEARING AID?	Yes 1<br>No 2	 2⇨CF6
**CF5**. WHEN USING HIS/HER HEARING AID, DOES (*name*) HAVE DIFFICULTY HEARING SOUNDS LIKE PEOPLES' VOICES OR MUSIC?<br><br>WOULD YOU SAY (*name*) HAS: NO DIFFICULTY, SOME DIFFICULTY, A LOT OF DIFFICULTY OR CANNOT DO AT ALL?	No difficulty 1<br>Some difficulty 2<br>A lot of difficulty 3<br>Cannot do at all 4	1⇨CF7<br>2⇨CF7<br>3⇨CF7<br>4⇨CF7
**CF6**. DOES (*name*) HAVE DIFFICULTY HEARING SOUNDS LIKE PEOPLES' VOICES OR MUSIC?<br><br>WOULD YOU SAY (*name*) HAS: NO DIFFICULTY, SOME DIFFICULTY, A LOT OF DIFFICULTY OR CANNOT DO AT ALL?	No difficulty 1<br>Some difficulty 2<br>A lot of difficulty 3<br>Cannot do at all 4	
**CF7**. DOES (*name*) USE ANY EQUIPMENT OR RECEIVE ASSISTANCE FOR WALKING?	Yes 1<br>No 2	 2⇨CF12
**CF8**. WITHOUT HIS/HER EQUIPMENT OR ASSISTANCE, DOES (*name*) HAVE DIFFICULTY WALKING 100 YARDS/METERS ON LEVEL GROUND? THAT WOULD BE ABOUT THE LENGTH OF 1 FOOTBALL FIELD. [OR INSERT COUNTRY SPECIFIC EXAMPLE].<br><br>WOULD YOU SAY (*name*) HAS: SOME DIFFICULTY, A LOT OF DIFFICULTY OR CANNOT DO AT ALL?	Some difficulty 2<br>A lot of difficulty 3	 3⇨CF10

	Cannot do at all 4	4⇨CF10
**CF9**. WITHOUT HIS/HER EQUIPMENT OR ASSISTANCE, DOES (*name*) HAVE DIFFICULTY WALKING 500 YARDS/METERS ON LEVEL GROUND? THAT WOULD BE ABOUT THE LENGTH OF 5 FOOTBALL FIELDS. [OR INSERT COUNTRY SPECIFIC EXAMPLE].<br><br>WOULD YOU SAY (*name*) HAS: SOME DIFFICULTY, A LOT OF DIFFICULTY OR CANNOT DO AT ALL?	Some difficulty 2<br>A lot of difficulty 3<br>Cannot do at all 4	
**CF10**. WITH HIS/HER EQUIPMENT OR ASSISTANCE, DOES (*name*) HAVE DIFFICULTY WALKING 100 YARDS/METERS ON LEVEL GROUND? THAT WOULD BE ABOUT THE LENGTH OF 1 FOOTBALL FIELD. [OR INSERT COUNTRY SPECIFIC EXAMPLE].<br><br>WOULD YOU SAY (*name*) HAS: NO DIFFICULTY, SOME DIFFICULTY, A LOT OF DIFFICULTY OR CANNOT DO AT ALL?	No difficulty 1<br>Some difficulty 2<br>A lot of difficulty 3<br>Cannot do at all 4	 <br>3⇨CF14<br>4⇨CF14
**CF11**. WITH HIS/HER EQUIPMENT OR ASSISTANCE, DOES (*name*) HAVE DIFFICULTY WALKING 500 YARDS/METERS ON LEVEL GROUND? THAT WOULD BE ABOUT THE LENGTH OF 5 FOOTBALL FIELDS. [OR INSERT COUNTRY SPECIFIC EXAMPLE].<br><br>WOULD YOU SAY (*name*) HAS: NO DIFFICULTY, SOME DIFFICULTY, A LOT OF DIFFICULTY OR CANNOT DO AT ALL?	No difficulty 1<br>Some difficulty 2<br>A lot of difficulty 3<br>Cannot do at all 4	1⇨CF14<br>2⇨CF14<br>3⇨CF14<br>4⇨CF14
**CF12**. COMPARED WITH CHILDREN OF THE SAME AGE, DOES (*name*) HAVE DIFFICULTY WALKING 100 YARDS/METERS ON LEVEL GROUND? THAT WOULD BE ABOUT THE LENGTH OF 1 FOOTBALL FIELD. [OR INSERT COUNTRY SPECIFIC EXAMPLE].	No difficulty 1	

WOULD YOU SAY (*name*) HAS: NO DIFFICULTY, SOME DIFFICULTY, A LOT OF DIFFICULTY OR CANNOT DO AT ALL?	Some difficulty 2<br>A lot of difficulty 3<br>Cannot do at all 4	 3⇨CF14<br>4⇨CF14
**CF13**. COMPARED WITH CHILDREN OF THE SAME AGE, DOES (*name*) HAVE DIFFICULTY WALKING 500 YARDS/METERS ON LEVEL GROUND? THAT WOULD BE ABOUT THE LENGTH OF 5 FOOTBALL FIELDS. [OR INSERT COUNTRY SPECIFIC EXAMPLE].<br><br>WOULD YOU SAY (*name*) HAS: NO DIFFICULTY, SOME DIFFICULTY, A LOT OF DIFFICULTY OR CANNOT DO AT ALL?	No difficulty 1<br>Some difficulty 2<br>A lot of difficulty 3<br>Cannot do at all 4	
**CF14**. DOES (*name*) HAVE DIFFICULTY WITH SELF-CARE SUCH AS FEEDING OR DRESSING HIM/HERSELF?<br><br>WOULD YOU SAY (*name*) HAS: NO DIFFICULTY, SOME DIFFICULTY, A LOT OF DIFFICULTY OR CANNOT DO AT ALL?	No difficulty 1<br>Some difficulty 2<br>A lot of difficulty 3<br>Cannot do at all 4	
**CF15**. WHEN (*name*) SPEAKS, DOES HE/SHE HAVE DIFFICULTY BEING UNDERSTOOD BY PEOPLE INSIDE OF THIS HOUSEHOLD?<br><br>WOULD YOU SAY (*name*) HAS: NO DIFFICULTY, SOME DIFFICULTY, A LOT OF DIFFICULTY OR CANNOT DO AT ALL?	No difficulty 1<br>Some difficulty 2<br>A lot of difficulty 3<br>Cannot do at all 4	
**CF16**. WHEN (*name*) SPEAKS, DOES HE/SHE HAVE DIFFICULTY BEING UNDERSTOOD BY PEOPLE OUTSIDE OF THIS HOUSEHOLD?<br><br>WOULD YOU SAY (*name*) HAS: NO DIFFICULTY, SOME DIFFICULTY, A LOT OF DIFFICULTY OR CANNOT DO AT ALL?	No difficulty 1<br>Some difficulty 2	

	A lot of difficulty 3<br>Cannot do at all 4	
**CF17**. COMPARED WITH CHILDREN OF THE SAME AGE, DOES (*name*) HAVE DIFFICULTY LEARNING THINGS?<br><br>WOULD YOU SAY (*name*) HAS: NO DIFFICULTY, SOME DIFFICULTY, A LOT OF DIFFICULTY OR CANNOT DO AT ALL?	No difficulty 1<br>Some difficulty 2<br>A lot of difficulty 3<br>Cannot do at all 4	
**CF18**. COMPARED WITH CHILDREN OF THE SAME AGE, DOES (*name*) HAVE DIFFICULTY REMEMBERING THINGS?<br><br>WOULD YOU SAY (*name*) HAS: NO DIFFICULTY, SOME DIFFICULTY, A LOT OF DIFFICULTY OR CANNOT DO AT ALL?	No difficulty 1<br>Some difficulty 2<br>A lot of difficulty 3<br>Cannot do at all 4	
**CF19**. DOES (*name*) HAVE DIFFICULTY CONCENTRATING ON AN ACTIVITY THAT HE/SHE ENJOYS DOING?<br><br>WOULD YOU SAY (*name*) HAS: NO DIFFICULTY, SOME DIFFICULTY, A LOT OF DIFFICULTY OR CANNOT DO AT ALL?	No difficulty 1<br>Some difficulty 2<br>A lot of difficulty 3<br>Cannot do at all 4	
**CF20**. DOES (*name*) HAVE DIFFICULTY ACCEPTING CHANGES IN HIS/HER ROUTINE?<br><br>WOULD YOU SAY (*name*) HAS: NO DIFFICULTY, SOME DIFFICULTY, A LOT OF DIFFICULTY OR CANNOT DO AT ALL?	No difficulty 1<br>Some difficulty 2<br>A lot of difficulty 3<br>Cannot do at all 4	
**CF21**. COMPARED WITH CHILDREN OF THE SAME AGE, DOES (*name*) HAVE DIFFICULTY CONTROLLING HIS/HER BEHAVIOUR?	No difficulty 1	

WOULD YOU SAY (*name*) HAS: NO DIFFICULTY, SOME DIFFICULTY, A LOT OF DIFFICULTY OR CANNOT DO AT ALL?	Some difficulty 2<br>A lot of difficulty 3<br>Cannot do at all 4	
**CF22**. DOES (*name*) HAVE DIFFICULTY MAKING FRIENDS?<br><br>WOULD YOU SAY (*name*) HAS: NO DIFFICULTY, SOME DIFFICULTY, A LOT OF DIFFICULTY OR CANNOT DO AT ALL?	No difficulty 1<br>Some difficulty 2<br>A lot of difficulty 3<br>Cannot do at all 4	
**CF23**. HOW OFTEN DOES (*name*) SEEM VERY ANXIOUS, NERVOUS OR WORRIED?<br><br>WOULD YOU SAY: DAILY, WEEKLY, MONTHLY, A FEW TIMES A YEAR OR NEVER?	Daily 1<br>Weekly 2<br>Monthly 3<br>A few times a year 4<br>Never 5	
**CF24**. HOW OFTEN DOES (*name*) SEEM VERY SAD OR DEPRESSED?<br><br>WOULD YOU SAY: DAILY, WEEKLY, MONTHLY, A FEW TIMES A YEAR OR NEVER?	Daily 1<br>Weekly 2<br>Monthly 3<br>A few times a year 4<br>Never 5	

Family information – Dari

این فورمه را جهت جمع آوری معلومات اساسی از نزد مراقبت کننده طفل تکمیل نمایید. بعضی از بخش های این فورمه دوباره دراخیر استفاده میشوند.

اسم مراقبت کننده: تاریخ:

اسم طفل: آفیسر های پروژه:

سوالات لوازم خانه:

1) چند نفر اعضای فامیل باهم زندگی میکنید؟

جوانان -----------

اطفال ------------

2) آیا خانه که زندگی میکنید کرایی است یا شخصی؟

☐ شخصی

☐ کرایی

☐ دیگر(مشخص سازید)

3) ساختمان خانه شما از چی ساخته شده است

☐ ابتدایی (خاک، ریگ، خار، گل)

☐ پیشرفته (سمنت، پلاستیک، کاشی)

☐ دیگر(مشخص سازید)

4) آیا یکی از اشیایی ذیل را در خانه دارید؟

رادیو: بلی ☐ نخیر☐

تلویزیون: بلی ☐ نخیر☐

مبایل: بلی ☐ نخیر☐

جنراتور: بلی ☐ نخیر☐

یخچال: بلی ☐ نخیر☐

5) وسایل اولیه تراسپورتیشن شما چی است؟

☐ موتر شخصی

☐ موتور سایکل شخصی

- بایسکل شخصی
- ترانسپورت عامه
- دیگر(مشخص سازید): ________________________

والدین/مراقبت کننده ها

6) مراقبت کننده ابتدایی طفل در خانه کی می باشد؟

خواهر	پدرکلان	مادر
برادر	خاله	پدر
دیگر	عمه	مادرکلان

7) آیا والدین یکجا زندگی می کنند؟

پدر: بلی نخیر، جدا شده نخیر، فوت شده نخیر، دیگر (مشخص سازید)

مادر: بلی نخیر، جدا شده نخیر، فوت شده نخیر، دیگر (مشخص سازید)

8) قومیت والدین چی است؟

پدر: پشتون تاجیک هزاره ازبیک دیگر (مشخص سازید)

مادر: پشتون تاجیک هزاره ازبیک دیگر (مشخص سازید)

9) سن والدین جقدر است؟

پدر: ___________

مادر: ___________

10) سطح تحصیلات والدین جقدر است؟

پدر: هیچ مدرسه ابتدایه متوسطه بالاتر از متوسطه

مادر: هیچ مدرسه ابتدایه متوسطه بالاتر از متوسطه

11) مادر چند طفل دارد؟

زنده: ______

فوت شده: ______

سن طفل کوچک: ______

سن طفل بزرگ: ______

پرسش های بعدی در مورد مشکلات والدین که در هنگام روبرو شدن با یک مشکل صحی چی می کنند مب پرسد.

پدر

1) با وجود پوشیدن عینک آیا باز هم در دید مشکل دارید؟

- نخیر، هیچ مشکل
- بلی، بعضی مشکلات
- بلی، بسیار مشل زیاد
- بلی، هیچ دیده نمی توانم

2) با وجود پوشیدن آله شنوایی آیا باز هم در شنیدن مشکل دارید؟

- نخیر، هیچ مشکل
- بلی، بعضی مشکلات
- بلی، بسیار مشل زیاد
- بلی، هیچ دیده نمی توانم

3) آیا در قدم زدن و بالا شدن بالای زینه مشکل دارید؟

- نخیر، هیچ مشکل
- بلی، بعضی مشکلات
- بلی، بسیار مشل زیاد
- بلی، هیچ دیده نمی توانم

4) آیا در دقت کردن و به یاد آوردن مشکل دارید؟

- نخیر، هیچ مشکل
- بلی، بعضی مشکلات
- بلی، بسیار مشل زیاد
- بلی، هیچ دیده نمی توانم

5) آیا در حفظ الصحه شخصی مشکل دارید؟

- نخیر، هیچ مشکل
- بلی، بعضی مشکلات
- بلی، بسیار مشل زیاد

مادر

1) با وجود پوشیدن عینک آیا باز هم در دید مشکل دارید؟

- نخیر، هیچ مشکل
- بلی، بعضی مشکلات
- بلی، بسیار مشل زیاد
- بلی، هیچ دیده نمی توانم

2) با وجود پوشیدن آله شنوایی آیا باز هم در شنیدن مشکل دارید؟

- نخیر، هیچ مشکل
- بلی، بعضی مشکلات
- بلی، بسیار مشل زیاد
- بلی، هیچ دیده نمی توانم

3) آیا در قدم زدن و بالا شدن بالای زینه مشکل دارید؟

- نخیر، هیچ مشکل
- بلی، بعضی مشکلات
- بلی، بسیار مشل زیاد
- بلی، هیچ دیده نمی توانم

4) آیا در دقت کردن و به یاد آوردن مشکل دارید؟

- نخیر، هیچ مشکل
- بلی، بعضی مشکلات
- بلی، بسیار مشل زیاد
- بلی، هیچ دیده نمی توانم

5) آیا در حفظ الصحه شخصی مشکل دارید؟

- نخیر، هیچ مشکل
- بلی، بعضی مشکلات
- بلی، بسیار مشل زیاد

□ بلی، هیچ دیده نمی توانم

□ بلی، هیچ دیده نمی توانم

6) آیا با استفاده از زبان عادی در بحث کردن بطور مثال در فهمیدن و فهماندن مشکل دارید؟

□ نخیر، هیچ مشکل

□ بلی، بعضی مشکلات

□ بلی، بسیار مشل زیاد

□ بلی، هیچ دیده نمی توانم

6) آیا با استفاده از زبان عادی در بحث کردن بطور مثال در فهمیدن و فهماندن مشکل دارید؟

□ نخیر، هیچ مشکل

□ بلی، بعضی مشکلات

□ بلی، بسیار مشل زیاد

□ بلی، هیچ دیده نمی توانم

طفل (طفل مورد هدف)

7) سن طفل ______ سال:

تاریخ تولد (زور/ ماه/ سال) ____/____/__________

8) طفل دارای کدام حالت مزمن صحی می باشد:

□ سیریپرال پلسی

□ اسپینیا بایفیدیا

□ سوی شکل ولادی اطراف

□ معلولیت تکلمی

□ صرع و میرگی

□ دیگر: ________________________

9) آیا طفل تمام واکسین ها را دریافت کرده است (BCG, measles, DPT, polio)

□ بلی

□ نخیر

□ نامطمعین

10) آیا طفل در 2 ماه گذشته مریض بود:

□ بلی (مشخص سازید: چند بار، : مشکل اساسی)

□ نخیر

11) آیا برای مشکل حاد صحی در دو ماه گذشته خدمات صحی برای طفل تان مهیا نموده اید:

□ بلی (مشخص سازید: چند بار، : کجا و چه تداوی)

□ نخیر

12) آیا در 2 ماه گذشته با عرضه کننده های خدمات صحی در تماس بوده اید:

□ بلی (مشخص سازید: چند بار، : کجا و چه تداوی)

□ نخیر

مودل یونسف و واشنگتن گروپ برای سن مناسب فعالیت های طفل تکمیل نماید

- 2 تا 4 سال
- از 5 تا 17 سال

اجرا کردن وظایف طفل (2-4 سال)	CF	
**CF1**. می خواهم در مورد مشکلات که ممکن طفل شما داشته باشد بپرسم.<br><br>آیا ....عینک می پوشد؟	بلی.................................... 1.<br>نخیر.................................. 2...	2⇨CF3

**CF2.** آیا زمانیکه ....عینک های خود را می پوشد مشکلی در دید دارد؟<br><br>آیا می توانی بگویی که............: هیچ مشکل ، کمی مشکل و زیاد مشکل و هیچ نمی تواند؟	هیچ مشکل ندارد......................1<br>کمی مشکل دارد......................2<br>بسیار مشکل دارد.....................3<br>هیچ نمی تواند...................4	1⇨CF4<br>2⇨CF4<br>3⇨CF4<br>4⇨CF4
**CF3.** آیا .... در دیدن مشل دارد؟<br><br>آیا می توانی بگویی که............: هیچ مشکل ، کمی مشکل و زیاد مشکل و هیچ نمی تواند؟	هیچ مشکل ندارد......................1<br>کمی مشکل دارد......................2<br>بسیار مشکل دارد.....................3<br>هیچ نمی تواند...................4	
**CF4.** آیا....... از آله شنوایی استفاده می کند؟	بلی.................................. 1.<br>نخیر.................................2...	2⇨CF6
**CF5.** آیا.......... زمانیکه آله شنوایی را استفاده می کند در شنیدن آواز مردم طور مثال آواز و یا صدا مشکل دارد؟<br><br>آیا می توانی بگویی که............: هیچ مشکل ، کمی مشکل و زیاد مشکل و هیچ نمی تواند؟	هیچ مشکل ندارد......................1<br>کمی مشکل دارد......................2<br>بسیار مشکل دارد.....................3<br>هیچ نمی تواند...................4	1⇨CF7<br>2⇨CF7<br>3⇨CF7<br>4⇨CF7

**CF6.** آیا........... زمانیکه آله شنوایی را استفاده می کند در شنیدن آواز مردم طور مثال آواز و یا صدا مشکل دارد؟<br><br>آیا می توانی بگویی که............: هیچ مشکل ، کمی مشکل و زیاد مشکل و هیچ نمی تواند؟	هیچ مشکل ندارد......................1<br>کمی مشکل دارد......................2<br>بسیار مشکل دارد.....................3<br>هیچ نمی تواند...................4	
**CF7.** آیا.......... وسایل کمکی و یا کمک از دیگران برای قدم زدن ضرورت دارد؟	Yes 1<br>No 2	2⇨CF10
**CF8.** بدون.......... وسایل کمکی و یا کمک از دیگران برای قدم زدن ضرورت دارد؟<br><br>آیا می توانی بگویی که............: کمی مشکل و زیاد مشکل و هیچ نمی تواند؟	کمی مشکل دارد......................2<br>بسیار مشکل دارد.....................3<br>هیچ نمی تواند...................4	
**CF9.** آیا........... زمانیکه آله شنوایی را استفاده می کند در شنیدن آواز مردم طور مثال آواز و یا صدا مشکل دارد؟<br><br>آیا می توانی بگویی که............: هیچ مشکل ، کمی مشکل و زیاد مشکل و هیچ نمی تواند؟	هیچ مشکل ندارد......................1<br>کمی مشکل دارد......................2<br>بسیار مشکل دارد.....................3<br>هیچ نمی تواند...................4	1⇨CF11<br>2⇨CF11<br>3⇨CF11<br>4⇨CF11
**CF10.** در مقایسه با اطفال هم سن و سال آیا .... در قدم زدن مشکل دارد؟<br><br>آیا می توانی بگویی که............: هیچ مشکل ، کمی مشکل و زیاد مشکل و هیچ نمی تواند؟	هیچ مشکل ندارد......................1<br>کمی مشکل دارد......................2<br>بسیار مشکل دارد.....................3<br>هیچ نمی تواند...................4	

**CF11.** در مقایسه با اطفال هم سن و سال آیا .... در بلند کردن آشیایی کوچک با دست خود مشکل دارد؟<br><br>آیا می توانی بگویی که............: هیچ مشکل ، کمی مشکل و زیاد مشکل و هیچ نمی تواند؟	هیچ مشکل ندارد......................1<br>کمی مشکل دارد......................2<br>بسیار مشکل دارد......................3<br>هیچ نمی تواند...................4	
**CF12.** آیا .... در فهماندن شما مشکل دارد ؟<br><br>آیا می توانی بگویی که............: هیچ مشکل ، کمی مشکل و زیاد مشکل و هیچ نمی تواند؟	هیچ مشکل ندارد......................1<br>کمی مشکل دارد......................2<br>بسیار مشکل دارد......................3<br>هیچ نمی تواند...................4	
**CF13.** وقتی .... صحبت می نماید آیا در فهمیدن صحبت های او مشکل دارید؟<br><br>آیا می توانی بگویی که............: هیچ مشکل ، کمی مشکل و زیاد مشکل و هیچ نمی تواند؟	هیچ مشکل ندارد......................1<br>کمی مشکل دارد......................2<br>بسیار مشکل دارد......................3<br>هیچ نمی تواند...................4	
**CF14.** در مقایسه با اطفال هم سن و سال آیا .... در آموختن مشکل دارد؟<br><br>آیا می توانی بگویی که............: هیچ مشکل ، کمی مشکل و زیاد مشکل و هیچ نمی تواند؟	هیچ مشکل ندارد......................1<br>کمی مشکل دارد......................2<br>بسیار مشکل دارد......................3<br>هیچ نمی تواند...................4	

**CF15.** در مقایسه با اطفال هم سن و سال آیا .... در بازی کردن مشکل دارد؟<br><br>آیا می توانی بگویی که............: هیچ مشکل ، کمی مشکل و زیاد مشکل و هیچ نمی تواند؟	هیچ مشکل ندارد......................1<br>کمی مشکل دارد......................2<br>بسیار مشکل دارد.....................3<br>هیچ نمی تواند...................4	
**CF16.** در مقایسه با اطفال هم سن و سال آیا .... در لگد زدن، چک زدن و یا در ضربه زدن مشکل دارد؟<br><br>آیا می توانی بگویی که............: هیچ مشکل ، با همان اندازه یا هیچ ،زیاد و بسیار زیاد؟	مجموعا هیچ..........................1<br>به همان اندازه یا هیچ...............2<br>زیاد.................................. 3<br>بسیار زیاد..........................4	

(اجرا کردن وظایف طفل (5-17 سال		CF
**CF1.** می خواهم در مورد مشکلات که ممکن طفل شما داشته باشد بپرسم.<br><br>آیا ....عینک می پوشد؟	بلی....................................1<br>نخیر................................... 2	2⇨CF3
**CF2.** آیا زمانیکه ....عینک های خود را می پوشد مشکلی در دید دارد؟<br><br>آیا می توانی بگویی که............: هیچ مشکل ، کمی مشکل و زیاد مشکل و هیچ دیده نمی تواند؟	هیچ مشکل ندارد......................1<br>کمی مشکل دارد.....................2<br>بسیار مشکل دارد.....................3	1⇨CF4<br>2⇨CF4<br>3⇨CF4<br>4⇨CF4

	هیچ نمی تواند...................4	
**CF3**. آیا .... در دیدن مشل دارد؟<br><br>آیا می توانی بگویی که............: هیچ مشکل ، کمی مشکل و زیاد مشکل و هیچ دیده نمی تواند؟	هیچ مشکل ندارد......................1<br>کمی مشکل دارد......................2<br>بسیار مشکل دارد.....................3<br>هیچ نمی تواند...................4	
**CF4**. آیا........ از آله شنوایی استفاده می کند؟	بلی...................................1<br>نخیر.................................. 2	2⇨CF6
**CF5**. آیا........... زمانیکه آله شنوایی را استفاده می کند در شنیدن آواز مردم طور مثال آواز و یا صدا مشکل دارد؟<br><br>آیا می توانی بگویی که............: هیچ مشکل ، کمی مشکل و زیاد مشکل و هیچ نمی تواند؟	هیچ مشکل ندارد......................1<br>کمی مشکل دارد......................2<br>بسیار مشکل دارد.....................3<br>هیچ نمی تواند...................4	1⇨CF7<br>2⇨CF7<br>3⇨CF7<br>4⇨CF7
**CF6**. آیا........... زمانیکه آله شنوایی را استفاده می کند در شنیدن آواز مردم طور مثال آواز و یا صدا مشکل دارد؟<br><br>آیا می توانی بگویی که............: هیچ مشکل ، کمی مشکل و زیاد مشکل و هیچ نمی تواند؟	هیچ مشکل ندارد......................1<br>کمی مشکل دارد......................2<br>بسیار مشکل دارد.....................3<br>هیچ نمی تواند...................4	
**CF7**. آیا......... وسایل کمکی و یا کمک از دیگران برای قدم زدن ضرورت دارد؟	بلی...................................1<br>نخیر.................................. 2	2⇨CF12

**CF8.** بغیر از وسایل کمکی و یا شخص کمکی آیا.........در قدم زدن به اندازه ای 100 متر در سطح هموار مشکل دارد؟ که معادل یک زمین فوتبال میشود<br><br>آیا می توانی بگویی که............: کمی مشکل و زیاد مشکل و هیچ نمی تواند؟	کمی مشکل دارد......................2<br>بسیار مشکل دارد.....................3<br>هیچ نمی تواند...................4	3⇨CF10<br>4⇨CF10
**CF9.** بغیر از وسایل کمکی و یا شخص کمکی آیا.........در قدم زدن به اندازه ای 500 متر در سطح هموار مشکل دارد؟ که معادل یک زمین فوتبال میشود<br><br>آیا می توانی بگویی که............: کمی مشکل و زیاد مشکل و هـ هیچ نمی تواند؟	کمی مشکل دارد......................2<br>بسیار مشکل دارد.....................3<br>هیچ نمی تواند...................4	
**CF10.** با وسایل کمکی و یا شخص کمکی آیا.........در قدم زدن به اندازه ای 100 متر در سطح هموار مشکل دارد؟ که معادل یک زمین فوتبال میشود<br><br>آیا می توانی بگویی که............:هیچ مشکل، کمی مشکل و زیاد مشکل و هیچ نمی تواند؟؟	هیچ مشکل ندارد......................1<br>کمی مشکل دارد......................2<br>بسیار مشکل دارد.....................3<br>هیچ نمی تواند...................4	3⇨CF14<br>4⇨CF14
**CF11.** با وسایل کمکی و یا شخص کمکی آیا.........در قدم زدن به اندازه ای 500 متر در سطح هموار مشکل دارد؟ که معادل یک زمین فوتبال میشود		

آیا می توانی بگویی که............:هیچ مشکل، کمی مشکل و زیاد مشکل و هیچ نمی تواند؟؟	هیچ مشکل ندارد......................1<br>کمی مشکل دارد......................2<br>بسیار مشکل دارد.....................3<br>هیچ نمی تواند...................4	1⇨CF14<br>2⇨CF14<br>3⇨CF14<br>4⇨CF14
**CF12.** در مقایسه با اطفال هم سن و سال خود آیا.........در قدم زدن به اندازه ای 100 متر در سطح هموار مشکل دارد؟ که معادل یک زمین فوتبال میشود<br><br>آیا می توانی بگویی که............:هیچ مشکل، کمی مشکل و زیاد مشکل و هیچ نمی تواند؟د؟	هیچ مشکل ندارد......................1<br>کمی مشکل دارد......................2<br>بسیار مشکل دارد.....................3<br>هیچ نمی تواند...................4	3⇨CF14<br>4⇨CF14
**CF13.** در مقایسه با اطفال هم سن و سال خود آیا.........در قدم زدن به اندازه ای 500 متر در سطح هموار مشکل دارد؟ که معادل یک زمین فوتبال میشود<br><br>آیا می توانی بگویی که............:هیچ مشکل، کمی مشکل و زیاد مشکل و هیچ نمی تواند؟	هیچ مشکل ندارد......................1<br>کمی مشکل دارد......................2<br>بسیار مشکل دارد.....................3<br>هیچ نمی تواند...................4	
**CF14.** آیا .... در حفظ الصحه شخصی بطور مثال غذ ا خوردن و لباس پوشیدن مشکل دارد؟<br><br>آیا می توانی بگویی که............:هیچ مشکل، کمی مشکل و زیاد مشکل و هیچ نمی تواند تواند؟	هیچ مشکل ندارد......................1<br>کمی مشکل دارد......................2<br>بسیار مشکل دارد.....................3<br>هیچ نمی تواند...................4	

**CF15.** زمانی که ..... صحبت می نماید آیا اعضای فامیل در خانواده سخنان وی را می فهمند؟<br><br>آیا می توانی بگویی که............:هیچ مشکل، کمی مشکل و زیاد مشکل و هیچ نمی تواند تواند؟	هیچ مشکل ندارد......................1<br>کمی مشکل دارد......................2<br>بسیار مشکل دارد.....................3<br>هیچ نمی تواند...................4	
**CF16.** زمانی که ..... صحبت می نماید آیا در بیرون از خانه سخنان وی را می فهمند؟<br><br>آیا می توانی بگویی که............:هیچ مشکل، کمی مشکل و زیاد مشکل و هیچ نمی تواند تواند؟	هیچ مشکل ندارد......................1<br>کمی مشکل دارد......................2<br>بسیار مشکل دارد.....................3<br>هیچ نمی تواند...................4	
**CF17.** در مقایسه با اطفال هم سن و سال خود آیا......... در اموختن مشکل دارد؟<br><br>آیا می توانی بگویی که............:هیچ مشکل، کمی مشکل و زیاد مشکل و هیچ نمی تواند تواند؟	هیچ مشکل ندارد......................1<br>کمی مشکل دارد......................2<br>بسیار مشکل دارد.....................3<br>هیچ نمی تواند...................4	
**CF18.** در مقایسه با اطفال هم سن و سال خود آیا......... در به یاد داشتن مشکل دارد؟<br><br>آیا می توانی بگویی که............:هیچ مشکل، کمی مشکل و زیاد مشکل و هیچ نمی تواند تواند؟	هیچ مشکل ندارد......................1<br>کمی مشکل دارد......................2	

	بسیار مشکل<br>دارد......................3<br>هیچ نمی تواند...................4	
**CF19.** آیا.......... در تمرکز کردن بالای فعالیت های که برایش دلچسپ است لذت می برد؟<br><br>آیا می توانی بگویی که............:هیچ مشکل، کمی مشکل و زیاد مشکل و هیچ نمی تواند تواند؟	هیچ مشکل<br>ندارد.......................1<br>کمی مشکل<br>دارد.......................2<br>بسیار مشکل<br>دارد......................3<br>هیچ نمی تواند...................4	
**CF20.** آیا .... در قبول کردن تغیرات در کار های روزمره خود مشکل دارد؟<br><br>آیا می توانی بگویی که............:هیچ مشکل، کمی مشکل و زیاد مشکل و هیچ نمی تواند تواند؟	هیچ مشکل<br>ندارد.......................1<br>کمی مشکل<br>دارد.......................2<br>بسیار مشکل<br>دارد......................3<br>هیچ نمی تواند...................4	
**CF21.** در مقایسه با اطفال هم سن و سال خود آیا......... در کنترول کردن شخصیت خود مشکل دارد؟<br><br>آیا می توانی بگویی که............:هیچ مشکل، کمی مشکل و زیاد مشکل و هیچ نمی تواند تواند؟	هیچ مشکل<br>ندارد.......................1<br>کمی مشکل<br>دارد.......................2<br>بسیار مشکل<br>دارد......................3<br>هیچ نمی تواند...................4	
**CF22.** آیا ...... در پیدا کردن دوست های جدید مشکل دارد؟<br><br>آیا می توانی بگویی که............:هیچ مشکل، کمی مشکل و زیاد مشکل و هیچ نمی تواند تواند؟	هیچ مشکل<br>ندارد.......................1<br>کمی مشکل<br>دارد.......................2	

	بسیار مشکل دارد.....................3<br>هیچ نمی تواند...................4	
**CF23.** معمولا به کدام اندازه ....عصبی، وارخطا و شدیدا متشنج معلوم میشود؟<br><br>آیا می توانی بگویی که روزمره، هفته وار، ماهوار، چندبار در سال و هیچکاه؟	روزمره.................................1<br>هفته وار................................2<br>ماهوار.................................3.<br>چند بار در سال.........................4<br>هیچگاه................................5...	
**CF24.** معمولا به کدام اندازه ....خیلی خفه و یا دیپرس معلوم میشود؟<br><br>آیا می توانی بگویی که روزمره، هفته وار، ماهوار، چندبار در سال و هیچکاه؟	روزمره.................................1<br>هفته وار................................2<br>ماهوار.................................3.<br>چند بار در سال.........................4<br>هیچگاه................................5...	

Family information – Pashto

د ښځينه معلوماتو پاڼه

له دی فورمی چخه د پالونکو نه د لمرنی او ابتدایی معلوماتو د تولولو لپاره استفاده وکړي ، همداسی ددی فورمی له حینی نورو برخونه به د اخيري معلوماتو د راتولو لپاره استفاده و شي.

د پالونکی نوم .... تاريخ ....

د ماشوم نوم .... د پروژي د مسؤل مامور نوم........

د کورني په اړه معلومات

څو کسان ستاسو په کورني کې له تاسو سره يو ځای ژوند کوي .

لويان ....

ماشومان ....

1. ستاسو کور خپل دي که کرایي دی .
   - خپل ....
   - کرایي...........
   - که نور.........
2. ستاسو د کورغولی له کومو موادو نه جوړ شويدي .
   - په ساده او محلي ډول [ختو، شگو,پروره، خاوری اويا سری نه]
   - په عصری او پاخه ډول[سيمينټ ،پلاستيک او يا کاشی]
   - که نور ....
3. آيا لاندي کوم وسايل په کور کې لري.

راديو	بلي	نه
تلويزون	بلي	نه
تلفون	بلي	نه
جنراتور	بلي	نه
سولر	بلي	نه

4. په ورځني ژوند کې له کوم ډول ترانسپورټ نه استفاده کوي ؟
5. - شخصي موټر او يا لاري موټر
   - خپل موټرسيکل او يا وړ موټر سيکل
   - شخصي بايسکيل
   - عام او يا شرکتی وسايل

- او یا نور...................

مور پلار/ پالونکی

6. څوک په کورني کې د ماشومانو پالنه کوي ؟

☐ مور ☐ نیکه ☐ خور

☐ پلار ☐ عمه ☐ ورور

☐ انا ☐ کاکا ☐ نور

7. آیا مور او پلار په کور کې یو ځای ژوند کوي؟

☐ پلار ☐ بلي ☐ نه تمویل ☐ نه ☐ نه او یا نور [خاص] ___

☐ مور ☐ بلي ☐ نه تمویل ☐ نه ☐ نه او یا نور [خاص] ___

8. تاسو د کوم قوم نه یاست ؟

پلار ☐ پښتون ☐ تاجک ☐ هزاره ☐ ازبک ☐ او یا نور [خاص]___

مور ☐ پښتون ☐ تاجک ☐ هزاره ☐ ازبک ☐ او یا نور [خاص]___

9. ستاسو د مور او پلار سن څومره دي؟

پلار ___

مور___

10. ستاسو د والیدینو د عالي تحصیلاتو درجه څومره ده؟

پلار ☐ بی سواده ☐ مدرسه ☐ ابتدائیه ☐ متوسطه ☐ عالي تحصیلات

مور ☐ بی سواده ☐ مدرسه ☐ ابتدائیه ☐ متوسطه ☐ عالي تحصیلات

11. مور څو ماشومان لري ؟

ژوندي ___

مړه ___

ماشومان ___

لویان ___

لاندي پوښتني به ستاسو نه ستاسو د کورني د فعالیتونو او مشکلاتو کوم
چې تاسو اطمنان کامل ولري [د یونیسف / واشګنتن ګروپ لنډ معلومات د
معلولیت په اړه]

مور	پلار
1. آیا تاسو د سترګوپه لید کی له دی سره چی عینکی اغوندی، ستونزه لري؟<br>□ نه .نه مشکل نلرم<br>□ بلي لږ مشکل لرم<br>□ بلي ډیر مشکل لرم<br>□ نه په هیڅ ډول استفاده نه شم کولاي.<br>2. آیا په آوریدلو کي ستونزه لري؟<br>□ نه .نه مشکل نلرم<br>□ بلي لږ مشکل لرم<br>□ بلي ډیر مشکل لرم<br>□ نه په هیڅ ډول استفاده نه شم کولاي<br>3. آیا تاسو په تګ او یا زیني ته په ختلو کي ستونزه لري؟<br>□ نه .نه مشکل نلرم<br>□ بلي لږ مشکل لرم<br>□ بلي ډیر مشکل لرم<br>□نه په هیڅ ډول استفاده نه شم کولاي<br>4. آیا تاسو په یاداښت او یا توجه کې ستونزه لري؟<br>□ نه .نه مشکل نلرم<br>□ بلي لږ مشکل لرم<br>□ بلي ډیر مشکل لرم<br>□نه په هیڅ ډول استفاده نه شم کولاي<br>5. آیا تاسو خپل حان ساتلی شی (لکه د زان په وینځلو کې او یا کالی اغوستلو کی ستونزه لري؟	1. آیا تاسو د سترګوپه لید کی له دی سره چی عینکی اغوندی، ستونزه لري؟<br>□ نه .نه مشکل نلرم<br>□ بلي لږ مشکل لرم<br>□ بلي ډیر مشکل لرم<br>□ نه په هیڅ ډول استفاده نه شم کولاي.<br>2. آیا په آوریدلو کي ستونزه لري؟<br>□ نه .نه مشکل نلرم<br>□ بلي لږ مشکل لرم<br>□ بلي ډیر مشکل لرم<br>□ نه په هیڅ ډول استفاده نه شم کولاي<br>3. آیا تاسو په تګ او یا زیني ته په ختلو کي ستونزه لري؟<br>□ نه .نه مشکل نلرم<br>□ بلي لږ مشکل لرم<br>□ بلي ډیر مشکل لرم<br>□نه په هیڅ ډول استفاده نه شم کولاي<br>4. آیا تاسو په یاداښت او یا توجه کې ستونزه لري؟<br>□ نه .نه مشکل نلرم<br>□ بلي لږ مشکل لرم<br>□ بلي ډیر مشکل لرم<br>□نه په هیڅ ډول استفاده نه شم کولاي<br>5. آیا تاسو خپل حان ساتلی شی (لکه د زان په وینځلو کې او یا کالی اغوستلو کی ستونزه لري؟<br>□ نه .نه مشکل نلرم

☐ نه .نه مشکل نلرم

☐ بلي لږ مشکل لرم

☐ بلي ډیر مشکل لرم

☐نه په هیڅ ډول استفاده نه شم کولاي

6. آیا تاسو عموماً په خپله ژبه د خریداري په وخت کې د پوهولو او راپوهولو یه وخت کې د ستونزو سره مخامخ یاست؟

☐ نه .نه مشکل نلرم

☐ بلي لږ مشکل لرم

☐ بلي ډیر مشکل لرم

7. ☐نه په هیڅ ډول استفاده نه شم کولاي

☐ بلي لږ مشکل لرم

☐ بلي ډیر مشکل لرم

☐نه په هیڅ ډول استفاده نه شم کولاي

6. آیا تاسو عموماً په خپله ژبه د خریداري په وخت کې د پوهولو او راپوهولو یه وخت کې د ستونزو سره مخامخ یاست؟

☐ نه .نه مشکل نلرم

☐ بلي لږ مشکل لرم

☐ بلي ډیر مشکل لرم

☐نه په هیڅ ډول استفاده نه شم کولاي

معیوب ماشوم

1. د ماشوم عمر ــــ کلونه
د تولد تاریخ [ورځ/ میاشت / کال : ــ/ــ/ــ

2. آماشوم کوم ډول مزمن روغتیایي حالت لري؟
-

- Cerebral palsy
- Spina bifida
- Congenital limb defect
- Intellectual disability
- Epilepsy/seizures
- Other(s): ________________________

-
-
- او یا نور ــــ

3. ایا ماشوم ته ټول واکیسنونه زرق شویدي [بی سی جي، میزل ، ډی پي ټي او پولیو]
- بلي
- نه
- مطمئن ندي

4. آیا ماشوم په تیرو دو میاشتوکې ډکوم دول ناروغی تیایي تیره کړي؟
- بلي [خاص : حو حلی او اصلی تکلیف ې] :ــــ
- نه

5. په تیرو دوو میاشتو کو تاسو د ماشوم د ناروغی د لپاره کوم ډول روغتیایي خدمتونه چمتو کړیدي؟
- بلي [خاص : د څو حلی :چیرته او کومه دول تداوی] :ــــ
- نه

6. آیا تاسو په تیرو دوو میاشتو کې د روغیتایی خدمتونه د چمتو کوونکې سره کومه اړیکه نیولې؟

□ بلي [خاص : د څه وخت لپاره : چیرته او کومه دول تداوی] :____

□ نه

دالاندی برخه [یونیسف/ واشنګټن ګروپ ماډل) دی ماشوم دی عمر سره سم تکمیل کړي

- د ۲. ۴ کاله پوري
- او یا د ۱۵.۵ کاله پوري

د ماشوم د فعالیت په اړه 2-4 کاله په عمر CF		
CF : 1غواړم چې ستاسو د ماشوم دی معیوبی په اړه څو پوښتني وکړم ایا (دی ماشوم نوم) عینیکی او یا دی سترګو پردی د لیدلو لپاره استعمالوی؟	۱ بلي ......<br>۲ نه	1⇨CF2
2CF: آیا (دی ماشوم نوم) سره له دی ی چی عینکې او یا دسترګو پردی اغوندی دی لیدو ستونزه لری ؟<br>آیا تاسوویل شی چی (دی ماشوم نوم) .نه مشکل نلری ، بلي ډیر مشکل لری، بلي لږ مشکل لری ، نه په هیڅ ډول استفاده نه شی کولاي	نه .نه مشکل نلری .... ۱ بلي لږ مشکل لری............... ۲<br>بلي ډیر مشکل لری .... ۳<br>نه په هیڅ ډول استفاده نه شی کولاي۴	1⇨CF4<br>2⇨CF4<br>3⇨CF4<br>4⇨CF4
3CF: آیا (دی ماشوم نوم) د سترګو په لید کې ستونزه لري ؟ نه .نه مشکل نلری ، بلي ډیر مشکل لری، بلي لږ مشکل لری ، نه په هیڅ ډول استفاده نه شی کولاي	نه .نه مشکل نلری .... ۱ بلي لږ مشکل لری............... ۲<br>بلي ډیر مشکل لری .... ۳<br>نه په هیڅ ډول استفاده نه شی کولاي۴	
4CF: آیا (دی ماشوم نوم) د غوزو په اوریدو کې ستونزه لري ؟	۱ بلي ......<br>۲ نه	2⇨CF6
5CF: کله چی له مصنوعی غوزنه استفاده کوی,آیا (دی ماشوم نوم) د نورو کسانو غږ او یا موسیقی آوریدلای شی<br>آیا تاسو یي ویلي شي چی (دی ماشوم نوم) نه .نه مشکل نلری ، بلي ډیر مشکل لری، بلي لږ مشکل لری ، نه په هیڅ ډول استفاده نه شی کولاي	نه .نه مشکل نلری .... ۱ بلي لږ مشکل لری............... ۲<br>بلي ډیر مشکل لری .... ۳<br>نه په هیڅ ډول استفاده نه شی کولاي۴	1⇨CF7<br>2⇨CF7<br>3⇨CF7<br>4⇨CF7
6CF: آیا ستاسو ماشوم دکوم ډول غږ او یا موزیک د آوریدو په وخت کي د ستونزو سره مخامخ کیږی ؟	نه .نه مشکل نلری .... ۱ بلي لږ مشکل لری............... ۲<br>بلي ډیر مشکل لری .... ۳<br>نه په هیڅ ډول استفاده نه شی کولاي۴	

آیا تاسو ېي ويلي شي چی (دی ماشوم نوم) نه .نه مشکل نلری ، بلي ډیر مشکل لری، بلي لږ مشکل لری ، نه په هیڅ ډول استفاده نه شی کولاي		
7CF: آیا (دی ماشوم نوم) دی تگ په وخت کی له کومووسایلوچخه کاراخلی اویا کومک ته اره لری؟	١ بلي ......<br>٢ نه	2⇨CF10
8CF: آیا (دی ماشوم نوم) ېی له کوم وسیلی او یا کومک نه د تگ په وخت کی ستونزه لری ؟<br>آیا تاسو ېي ويلي شي چی (دی ماشوم نوم) بلي ډیر مشکل لری، بلي لږ مشکل لری ، نه په هیڅ ډول استفاده نه کوي	بلي لږ مشکل لری.............. ١<br>بلي ډیر مشکل لری .... ٢<br>نه په هیڅ ډول استفاده نه شی کولاي٣	
9CF: آیا (دی ماشوم نوم) ېی دی چی له کوم وسیلی او یا کومک نه کار واخلی دی تگ په وخت کی ستونزه لري؟<br>آیا تاسو ېي ويلي شي چی (دی ماشوم نوم) نه .نه مشکل نلری ، بلي ډیر مشکل لری، بلي لږ مشکل لری ، نه په هیڅ ډول استفاده نه شی کولاي	نه .نه مشکل نلری .... ١<br>بلي لږ مشکل لری............. ٢<br>بلي ډیر مشکل لری .... ٣<br>نه په هیڅ ډول استفاده نه شی کولاي۴	1⇨CF11<br>2⇨CF11<br>3⇨CF11<br>4⇨CF11
10CF: د یو بل روغ ماشو م سره ېی مقایسه کړي چې یو شان عمر ولري ایا (دی ماشوم نوم) دی تگ ستونزه لری؟<br>آیا تاسو ېي ويلي شي چی (دی ماشوم نوم) نه .نه مشکل نلری ، بلي ډیر مشکل لری، بلي لږ مشکل لری ، نه په هیڅ ډول استفاده نه شی کولاي	نه .نه مشکل نلری .... ١<br>بلي لږ مشکل لری............. ٢<br>بلي ډیر مشکل لری .... ٣<br>نه په هیڅ ډول استفاده نه شی کولاي۴	3⇨CF14<br>4⇨CF14
11CF: : د یو بل روغ ماشو م سره ېی مقایسه کړي چې یو شان عمر ولري ایا(دی ماشوم نوم) پخپله کولای شی چی په لاسو سره یو کوچنی شی پورته کری؟<br>آیا تاسو ېي ويلي شي (دی ماشوم نوم) نه .نه مشکل نلری ، بلي ډیر مشکل لری، بلي لږ مشکل لری ، نه په هیڅ ډول استفاده نه شی کولاي	نه .نه مشکل نلری .... ١<br>بلي لږ مشکل لری............. ٢<br>بلي ډیر مشکل لری .... ٣<br>نه په هیڅ ډول استفاده نه شی کولاي۴	1⇨CF14<br>2⇨CF14<br>3⇨CF14<br>4⇨CF14
12CF: آیا(دی ماشوم نوم) د تاسو په خبرو پوهیدو کی ستونزه لري؟<br>آیا تاسو ېي ويلي شي (دی ماشوم نوم) نه .نه مشکل نلری ، بلي ډیر مشکل لری، بلي لږ مشکل لری ، نه په هیڅ ډول استفاده نه شی کولاي	نه .نه مشکل نلری .... ١<br>بلي لږ مشکل لری............. ٢<br>بلي ډیر مشکل لری .... ٣<br>نه په هیڅ ډول استفاده نه شی کولاي۴	3⇨CF14<br>4⇨CF14
13CF: آیا کله چې (دی ماشوم نوم) تاسوسره خبری کوی ایا دی هغو دخبرو په پوهیدو کي ستونزه لري؟<br>آیا تاسو ېي ويلي شي نه .نه مشکل نلری ، بلي ډیر مشکل لری، بلي لږ مشکل لری ، نه په هیڅ ډول استفاده نه شی کولاي	نه .نه مشکل نلری .... ١<br>بلي لږ مشکل لری.............. ٢	

	بلي ډیر مشکل لری .... ۳<br>نه په هیڅ ډول استفاده نه شی کولاي۴	
14CF: د نورو روغو مااشومانو پر تله چی په همدی عمر وی, ایا(دی ماشوم نوم) په زده کره کی ستونزی لری؟<br>آیا تاسو یي ویلي شي (دی ماشوم نوم) نه .نه مشکل نلری ، بلي ډیر مشکل لری، بلي لږ مشکل لری ، نه په هیڅ ډول استفاده نه شی کولاي	نه .نه مشکل نلری .... ۱ بلي لږ مشکل لری................ ۲<br>بلي ډیر مشکل لری .... ۳<br>نه په هیڅ ډول استفاده نه شی کولاي۴	
15CF: د نورو روغو مااشومانو پر تله چی په همدی عمر وی, ایا (دی ماشوم نوم) د نورو روغو مااشومانو پر تله چی په همدی عمر وی, په لوبوکولو کی ستونزی لری؟<br>آیا تاسو یي ویلي شي (دی ماشوم نوم) نه .نه مشکل نلری ، بلي ډیر مشکل لری، بلي لږ مشکل لری ، نه په هیڅ ډول استفاده نه شی کولاي	نه .نه مشکل نلری .... ۱ بلي لږ مشکل لری................ ۲<br>بلي ډیر مشکل لری .... ۳<br>نه په هیڅ ډول استفاده نه شی کولاي۴	
16CF: د نورو روغو مااشومانو پر تله چی په همدی عمر وی, ایا (دی ماشوم نوم) پکومه کچه نورو ماشومان او یا غتان په لغتوی, چیچی او وهی ؟<br>آیا تاسو یي ویلي شي (دی ماشوم نوم) نه .هیچکله ، بلي لکه نور ماشومان او لږ کم، زیات او یا بیخی زیات	نه .نه مشکل نلری .... ۱ بلي لږ مشکل لری................ ۲<br>بلي ډیر مشکل لری .... ۳<br>نه په هیڅ ډول استفاده نه شی کولاي۴	

د ماشوم د فعالیت په اړه 5-17 کاله په عمر	CF	
CF : 1غواړم چې (دماشوم نوم) دی معیوبی په اړه څو پوښتني وکړم ایا (دی ماشوم نوم) عینکی او یا دی سترگو پردی د لیدلو لپاره استعمالوی؟	۱ بلي ......<br>۲ نه	1⇨CF2
2CF: آیا (دی ماشوم نوم) سره له دی ی چی عینکی او یا دسترگو پردی اغوندی دی لیدو ستونزه لری ؟<br>آیا تاسوویل شی چی (دی ماشوم نوم) .نه مشکل نلری ، بلي ډیر مشکل لری، بلي لږ مشکل لری ، نه په هیڅ ډول استفاده نه شی کولاي	نه .نه مشکل نلری .... ۱ بلي لږ مشکل لری................ ۲<br>بلي ډیر مشکل لری .... ۳<br>نه په هیڅ ډول استفاده نه شی کولاي۴	1⇨CF4<br>2⇨CF4<br>3⇨CF4<br>4⇨CF4
3CF: آیا (دی ماشوم نوم) د سترگو په لید کې ستونزه لري ؟ نه .نه مشکل نلری ، بلي ډیر مشکل لری، بلي لږ مشکل لری ، نه په هیڅ ډول استفاده نه شی کولاي	نه .نه مشکل نلری .... ۱ بلي لږ مشکل لری................ ۲<br>بلي ډیر مشکل لری .... ۳<br>نه په هیڅ ډول استفاده نه شی کولاي۴	
4CF: آیا (دی ماشوم نوم) د غوزو په اوریدو کې ستونزه لري ؟	۱ بلي ......<br>۲ نه	2⇨CF6
5CF: کله چی له مصنوعی غوزنه استفاده کوی,آیا (دی ماشوم نوم) د نورو کسانو غږ او یا موسیقی آوریدلای شی<br>آیا تاسو يي ويلي شي چی (دی ماشوم نوم) نه .نه مشکل نلری ، بلي ډیر مشکل لری، بلي لږ مشکل لری ، نه په هیڅ ډول استفاده نه شی کولاي	نه .نه مشکل نلری .... ۱ بلي لږ مشکل لری................ ۲<br>بلي ډیر مشکل لری .... ۳<br>نه په هیڅ ډول استفاده نه شی کولاي۴	1⇨CF7<br>2⇨CF7<br>3⇨CF7<br>4⇨CF7
6CF: آیا (دماشوم نوم) دکوم ډول غږ او یا موزیک د آوریدو په وخت کې د ستونزو سره مخامخ کیږی ؟<br>آیا تاسو يي ويلي شي چی (دی ماشوم نوم) نه .نه مشکل نلری ، بلي ډیر مشکل لری، بلي لږ مشکل لری ، نه په هیڅ ډول استفاده نه شی کولاي	نه .نه مشکل نلری .... ۱ بلي لږ مشکل لری................ ۲<br>بلي ډیر مشکل لری .... ۳<br>نه په هیڅ ډول استفاده نه شی کولاي۴	
7CF: آیا (دی ماشوم نوم) دی تگ په وخت کی له کومووسایلوچخه کاراخلی اویا کومک ته اره لری؟	۱ بلي ......<br>۲ نه	2⇨CF10
8CF: آیا (دی ماشوم نوم) بی دی چی له کوم وسیلی او یا کومک نه کار واخلی دی هواری زمکی سلومترو دی فاصلی دی تگ په وخت کی مشکل لري ؟<br>آیا تاسو يي ويلي شي چی (دی ماشوم نوم) بلي ډیر مشکل لری، بلي لږ مشکل لری ، نه په هیڅ ډول استفاده نه شی کولاي	بلي لږ مشکل لری................ ۱<br>بلي ډیر مشکل لری .... ۲<br>نه په هیڅ ډول استفاده نه شی کولاي۳	

9CF: آيا (دى ماشوم نوم) ٻى دى چى له کوم وسيلى او يا کومک نه کار واخلى دى هوارى زمکى 500 مترو دى فاصلى دى تگ په وخت کى ستونزه لري؟<br>آيا تاسو يي ويلي شي چي (دى ماشوم نوم) نه .نه مشکل نلرى ، بلي ډير مشکل لرى، بلي لږ مشکل لرى ، نه په هيڅ ډول استفاده نه شى کولاي	نه .نه مشکل نلرى .... ١ بلي لږ مشکل لرى............ ٢<br>بلي ډير مشکل لرى .... ٣<br>نه په هيڅ ډول استفاده نه شى کولاي۴	1⇨CF11<br>2⇨CF11<br>3⇨CF11<br>4⇨CF11
10CF: برسيره له دى چى له وسيلى كار اخلى او ياله مرستى سره ايا (د ماشوم نوم) دى 100 دهوارى زمکى د فاصلى دى تگ سره ستونزه لرى؟<br>آيا تاسو يي ويلي شي چي (دى ماشوم نوم) نه .نه مشکل نلرى ، بلي ډير مشکل لرى، بلي لږ مشکل لرى ، نه په هيڅ ډول استفاده نه شى کولاي	نه .نه مشکل نلرى .... ١ بلي لږ مشکل لرى............ ٢<br>بلي ډير مشکل لرى .... ٣<br>نه په هيڅ ډول استفاده نه شى کولاي۴	3⇨CF14<br>4⇨CF14
11CF: برسيره له دى چى له وسيلى كار اخلى او ياله مرستى سره ايا (د ماشوم نوم) د500 مترو دهوارى زمکى د فاصلى دى تگ سره ستونزه لرى؟<br>آيا تاسو يي ويلي شي چي (دى ماشوم نوم) نه .نه مشکل نلرى ، بلي ډير مشکل لرى، بلي لږ مشکل لرى ، نه په هيڅ ډول استفاده نه شى کولاي	نه .نه مشکل نلرى .... ١ بلي لږ مشکل لرى............ ٢<br>بلي ډير مشکل لرى .... ٣<br>نه په هيڅ ډول استفاده نه شى کولاي۴	1⇨CF14<br>2⇨CF14<br>3⇨CF14<br>4⇨CF14
12CF: د نورو روغو مااشومانو پر تله چى په همدى عمر وى, ايا (دى ماشوم نوم) د 100مترو هوارى زمکى دى فاصلى په تگ کى ستتوونزه لررى؟<br>آيا تاسو يي ويلي شي (دى ماشوم نوم) نه .نه مشکل نلرى ، بلي ډير مشکل لرى، بلي لږ مشکل لرى ، نه په هيڅ ډول نه شى کولاي	نه .نه مشکل نلرى .... ١ بلي لږ مشکل لرى............ ٢<br>بلي ډير مشکل لرى .... ٣<br>نه په هيڅ ډول استفاده نه شى کولاي۴	3⇨CF14<br>4⇨CF14
13CFد نورو روغو مااشومانو پر تله چى په همدى عمر وى, ايا (دى ماشوم نوم) د 500مترو هوارى زمکى دى فاصلى په تگ کى ستتوونزه لررى؟<br>آيا تاسو يي ويلي شي (دى ماشوم نوم) نه .نه مشکل نلرى ، بلي ډير مشکل لرى، بلي لږ مشکل لرى ، نه په هيڅ ډول نه شى کولاي	نه .نه مشکل نلرى .... ١<br>بلي لږ مشکل لرى............ ٢<br>بلي ډير مشکل لرى .... ٣<br>نه په هيڅ ډول استفاده نه شى کولاي۴	
14CF: ايا)دماشوم نوم) د زان په موظفت کى لکه خواره خورل او يا دکاليو په د اغوستلو په وخت د ستونزو سره مخامخ کيري؟<br>آيا تاسو يي ويلي شي نه .نه مشکل نلرى ، بلي ډير مشکل لرى، بلي لږ مشکل لرى ، نه په هيڅ ډول استفاده نه شى کولاي	نه .نه مشکل نلرى .... ١ بلي لږ مشکل لرى............ ٢<br>بلي ډير مشکل لرى .... ٣<br>نه په هيڅ ډول استفاده نه شى کولاي۴	
15CF: کله چى خبرى کوى (دماشوم نوم) په کورني کي نورغرى ٻى له خبرو سره ستونزه لري ؟	نه .نه مشکل نلرى .... ١ بلي لږ مشکل لرى............ ٢<br>بلي ډير مشکل لرى ٣	

آیا تاسو يې ويلي شي (دماشوم نوم) نه .نه مشکل نلری ، بلي ډیر مشکل لری، بلي لږ مشکل لری ، نه په هيڅ ډول نه شی کولاي	نه په هيڅ ډول استفاده نه شی کولاي۴	
16CF کله چی خبری کوی (دماشوم نوم) د کورني کې د نوروغرو له خبررو سره ستونزه لري ؟<br>آیا تاسو يې ويلي شي (دماشوم نوم) نه .نه مشکل نلری ، بلي ډیر مشکل لری، بلي لږ مشکل لری ، نه په هيڅ ډول استفاده نه شی کولاي	نه .نه مشکل نلری .... ۱ بلي لږ مشکل لری............... ۲<br>بلي ډیر مشکل لری .... ۳<br>نه په هيڅ ډول استفاده نه شی کولاي۴	
17CF: تاسو خپل (دماشوم نوم) د اشياو د زده کولو په جريان کې د بل روغ ههمزولی ماشوم سره مقايسه کړې چې څه ډول ستونزه لري؟<br>آیا تاسو يې ويلي شي (دماشوم نوم) نه .نه مشکل نلری ، بلي ډیر مشکل لری، بلي لږ مشکل لری ، نه په هيڅ ډول استفاده نه شی کولاي	نه .نه مشکل نلری .... ۱ بلي لږ مشکل لری............... ۲<br>بلي ډیر مشکل لری .... ۳<br>نه په هيڅ ډول استفاده نه شی کولاي۴	
18CF: تاسو يې ويلي شي (دماشوم نوم) د بل روغ همزولی ماشوم سره مقايسه کړې چې د اشياوو په يادولو کې څه ډول ستونزه لري؟<br>آیا تاسو يې ويلي شي نه .نه مشکل نلری ، بلي ډیر مشکل لری، بلي لږ مشکل لری ، نه په هيڅ ډول استفاده نه شی کولاي	نه .نه مشکل نلری .... ۱ بلي لږ مشکل لری............... ۲<br>بلي ډیر مشکل لری .... ۳<br>نه په هيڅ ډول استفاده نه شی کولاي۴	
19CF: آیا (دماشوم نوم) د فکری تمرکز ستونزه لري خصوصا دهغه فعايت په جريان کې چی ورنه خوند اخلي؟<br>آیا تاسو يې ويلي شي (دماشوم نوم) نه .نه مشکل نلری ، بلي ډیر مشکل لری، بلي لږ مشکل لری ، نه په هيڅ ډول استفاده نه شی کولاي	نه .نه مشکل نلری .... ۱ بلي لږ مشکل لری............... ۲<br>بلي ډیر مشکل لری .... ۳<br>نه په هيڅ ډول استفاده نه شی کولاي۴	
20CF: آیا (دماشوم نوم) د خپل عادت په تغيرکی اشياوو د قبلولو کې څه ډول ستونزه لري؟<br>آیا تاسو يې ويلي شي نه .نه مشکل نلری ، بلي ډیر مشکل لری، بلي لږ مشکل لری ، نه په هيڅ ډول استفاده نه شی کولاي	نه .نه مشکل نلری .... ۱ بلي لږ مشکل لری............... ۲<br>بلي ډیر مشکل لری .... ۳<br>نه په هيڅ ډول استفاده نه شی کولاي۴	
21CF: تاسو يې ويلي شي (دماشوم نوم) د بل روغ همزولی ماشوم سره مقايسه کړې چې د ځان دبرخورد په کنترول کې څه ډول ستونزه لري؟<br>آیا تاسو يې ويلي شي نه .نه مشکل نلری ، بلي ډیر مشکل لری، بلي لږ مشکل لری ، نه په هيڅ ډول استفاده نه شی کولاي	نه .نه مشکل نلری .... ۱ بلي لږ مشکل لری............... ۲<br>بلي ډیر مشکل لری .... ۳<br>نه په هيڅ ډول استفاده نه شی کولاي۴	
22CF: آیا (دماشوم نوم) د ملګرو په پيدا کولو کې څه ډول ستونزه لري؟<br>آیا تاسو يې ويلي شي چی (دماشوم نوم) ی نه .نه مشکل نلری ، بلي ډیر مشکل لری، بلي لږ مشکل لری ، نه په هيڅ ډول استفاده نه شی کولاي	نه .نه مشکل نلری .... ۱ بلي لږ مشکل لری............... ۲<br>بلي ډیر مشکل لری .... ۳<br>نه په هيڅ ډول استفاده نه شی کولاي۴	
23CF: ايا (دماشوم نوم) کله بد حالت ،تشويش حالت او خراب حالت ظاهروی ؟	ورځ ....۱<br>هفته ۲	

کولای شي ووایاست په / ورځ /هفته / میاشت / یو څه وخت یا کال او یا هیڅکله	میاشت................................٣<br>کال او یا یو څه وخت................۴<br>هیڅکله ۵	
24CF: آیا (دماشوم نوم) اکثره دیر خپه او یا ژور خفګان ته ځي ؟<br>کولای شي ووایاست په / ورځ /هفته / میاشت / یو څه وخت یا کال او یا هیڅکله	ورځ ....١<br>هفته ....٢<br>میاشت................................٣<br>کال او یا یو څه وخت................۴<br>هیڅکله ۵	

PedsQL Family Impact - Dari

رهنمایی ها

- این سروی می خواهد بداند که مراقبت کننده ها چه احساسی در مورد مراقبت از اطفال خود دارند.
- شما این سروی را بالای مراقبت کننده های اجرا می کنید که در این پروگرام اشتراک می نمایند **(i)** پیش از شروع پروگرام و **(ii)** بعد از ختم پروگرام.
- پیش از انجام سروی:
  - برای اشتراک کننده ها به زبان عامیانه بیانیه ېي را آماده و تشریح نماید که پروگرام اطفال قدرتمند چی می باشد، و ما می خواهیم آن را ارزیابی نمایم.
  - فورم رضایت را تکمیل نماید و رضایت شفایی را به دست بیاورید.
- برای اشتراک کننده ها توضیح بدهید که:
  - خانواده های اطفال معلول بعضی اوقات نگرانی های خاصی و یا مشکلاتی در خصوص صحت طفل خود دارند.
  - این سروی در بر گیرنده لیست از اشیا می باشد می می تواند مشکل برای شما و فامیل شما باشد.
  - لطفا برای ما توضیح بدهید که هر یک از مشکلاتی ذیل برای شما در ماه گذشته واقع گردیده است: هیچگاه، تقریبا هیچگاه، بعضی اوقات، اغلب اوقات و تقریبا همیشه.
  - هیچ سوالی صحیح و غلط نمی باشد.
  - اگر ودام سوالی را نفهمیدید میتوانید دوباره بپرسید.
  - فهم شان را برسی نماید، سوال نماید.
- و بعدا سروی را آغاز نماید با خواندن بیانیه و گزینه ها برای هر سوال. و عکسالعمل اشتراک کننده ها را علامه گذاری نماید.

هیچگاه	تقریبا هیچگاه	بعضی اوقات	اغلبا	معمولا همیشه

در یک ماه گذشته، نظر به نتیجه صحت طفل تان، به چه اندازه مشکل داشتید با:

	عملکرد فزیکی	هیچگاه	تقریبا هیچگاه	بعضی اوقات	اغلبا	تقریبا همیشه
1	از طرف روز احساس خسته گی می کردم					
2	صبح زمانی که از خواب بیدار میشدم احساس خسته گی می کردم					
3	بیش از حد احساس خسته گی میکردم در تا اجرای وظایف مورد علاقه ام را انجام دهم					
4	سر درد می شدم					
5	از لحاظ فزیکی احساس ضعیفی می کردم					

6	احساس بسیار ناراحتی درونی میکردم					

در یک ماه گذشته، نظر به نتیجه صحت طفل تان، به چه اندازه مشکل داشتید با:

	عملکرد عاطفی	هیچگاه	تقریبا هیچگاه	بعضی اوقات	اغلبا	تقریبا همیشه
7	احساس اضطراب میکردم					
8	احساس غمگینی میکردم					
9	احساس عصبانیت میکردم					
10	احساس ناگذیری میکردم					
11	احساس درمانده گی و نا امیدی می کردم					

	عملکرد اجتماعی	هیچگاه	تقریبا هیچگاه	بعضی اوقات	اغلبا	تقریبا همیشه
12	از دیگران احساس کناره گیری می کردن					
13	برای گرفتن کمک از دیگران مشکل داشتم					
14	برای فعالیت های اجتماعی وقت پیدا نمی کردم					
15	برای فعالیت های اجتماعی به اندازه کافی انرژی نداشتم					

	عملکرد فکری	هیچگاه	تقریبا هیچگاه	بعضی اوقات	اغلبا	تقریبا همیشه
16	برایم مشکل بود که بالای اشیا تمرکز خویش را حفظ نمایم					
17	برایم مشکل بود چیزی را که دیگران برایم می گویند به خاطر داشته باشم					

18	برایم مشکل بود تا چیزی را که شنیده ام به خاطر داشته باشم					
19	برایم مشکل بود تا سریع فکر نمایم					
20	برایم مشکل بود تا خاطر بیاورم چیزی را که فکر میکردم					

	ارتباطات	هیچگاه	تقریبا هیچگاه	بعضی اوقات	اغلبا	تقریبا همیشه
21	احساس میکردم که کسی وضیعت فامیلم را درک نمی کند					
22	برایم مشکل بود تا در مورد صحت طفلم با دیگران صحبت نمایم					
23	برایم مشکل بود تا به داکتر ها و نرس ها احساسم را ابراز نمایم					

در یک ماه گذشته، نظر به نتیجه صحت طفل تان، به چه اندازه مشکل داشتید با:

	نگرانی	هیچگاه	تقریبا هیچگاه	بعضی اوقات	اغلبا	تقریبا همیشه
24	نگران بودم که آیا تداوی طبی طفلم نتیجه می دهد یا خیر					
25	نگران بودم در مورد عوارض جانبی ادویه جات و تداوی طبی طفلم					
26	نگران بودم که عکس العمل دیگران در قبال وضیعت طفلم چه گونه باشد					
27	نگران بودم که مریضی طفلم بالای دیگر اعضای فامیلم چه اثَر میگذارد					
28	نگران آینده طفلم بودم					

در ذیل لیست از اشیا که می تواند برای فامیل شما یک مشکل باشد. لطفا برای ما بگوید به کدام اندازه از هریک از مشکلات برای فامیل شما در یک ماه گذشته واقع گردیده است.

در یک ماه گذشته، منحیث نتیجه صحت طفل تان، به چه اندازه یکی از مشکلات را فامیل شما داشت با:

	فعالیت های روزمره	هیچگاه	تقریبا هیچگاه	بعضی اوقات	اغلبا	تقریبا همیشه
29	فعالیت های فامیلی کوشش و وقت زیاد را میگرفت					
30	مشکل بود که برای انجام دادن وظایف خانه وقت پیدا کنم					
31	در انجام دادن وظایف خانه احساس خسته گی بی اندازه میکردم					

	ارتباطات فامیلی	**Never**	**Almost never**	**Sometimes**	**Often**	**Almost always**
32	کم بودن ارتباطات بین اعضای خانواده					
33	درگیری میان اعضای خانواده					
34	مشکل در تصمیم گرفتن به شکل یک خانواده					
35	مشکل در حل کردن مشکلات خانواده گی با اعضای فامیل					
36	اضراب و تشویش میان اعضای خانواده					

PedsQL Family Impact - Pashto

لارښوونې:

- د دی سروی په مرسته غواړو چی مراقبت کونکی د خپل ماشوم مراقبت په هکله څه احساس لریږ
- تاسی دا سروی په هغو کسانو تطبیق کوی چی په دی پروګرام کی ګډون کوی (i)د پروګرام پیل څخه وړاندی(ii) او د پروګرام د ختم څخه ورسته
- مخکی د سروی کولو څخه:
  - د ګډونکونو لپاره په ډیره عامیانه ژبه بیانیه آماده او تشریح ګړی چی د بیاوړی ماشومانو پروګرام څه شی دی، او مونږ غواړو دا پروګرام ارزیابی کړوږ
  - د رضایت فورم تکمیل او شفاہی رضایت لاس ته راوړی.
- ګډنکونکو ته تشریح کړی چی:
  - د معلولو ماشومانو کورنی کله کله خاص تشویشونه او یا مشکلات د خپل و ماشوم په هکله لری
  - په دی سروی کی داسی یو لړ شیان شامل دی چی کیدای شی ستاسی او یا ستاسی د کورنی ته مشکل وی.
  - لطفا مونږ ته ډاګه کړی چی په تیری یوی میاث کی کوم یو د لاندی مشکلاتو څخه تاسو ته پیښ شوی دی: هیڅکله، تقریبا هیڅکله، کله ناکله، کله کله او تقریبا همیشه.
  - په لاندی پوښتونو کی هیڅ یو صحیح او غلطه پوښتنه نشته.
  - که په کومه پوښتنه پوه نشوی کولای شی بیاځلی وپوښتیږ
  - د ګډونکونکو فهم ارزیابی کړی او وګوری چی څوک کومه پوښتنه لریږ
- او ورسته سروی شروع کړی.

هیڅکله	تقریبا هیڅکله	کله ناکله	کله کله	تقریبا همیشه

په تیری یوی میاشت کی، ستاسو د ماشوم د صحت نتیجی ته په کتو، تاسی په کومه اندازه مشکلات درلودل:

	فزیکی فعالیت	هیڅکله	تقریبا هیڅکله	کله ناکله	کله کله	تقریبا همیشه
1	دورزی له خوا احساس دی ستریا کوم					
2	کله چی سهار له خوبه پاځم دی ستریا احساس کوم					
3	د خپلی خوښی د وظیفی د اجرا کولو په وخت کی احساس د ستریا کوم					
4	سر درد کیدل					
5	د فیزیکی له لحاظه احساس د ضعیفی کوم					
6	زه په زړه کی ډیر خپګان احساسوم					

په تیری یوی میاشت کی، ستاسو د ماشوم د صحت نتیجی ته په کتو، تاسی په کومه اندازه مشکلات درلودل:

	احساساتي دنده	هیڅکله	تقریبا هیڅکله	کله ناکله	کله کله	تقریبا همیشه
7	د دار احساس می کولو					
8	د قهر احساس می کولو					
9	د قهر احساس می کولو					
10	د درمادندگی احساس می کولو					
11	د بی وسی او نا امیدی احساس می کولو					

	تولنیزی دندی	هیڅکله	تقریبا هیڅکله	کله ناکله	کله کله	تقریبا همیشه
12	له نورو خلکو څخه می د جداپي احساس کولو					
13	له نورو څخه می په مرسته غوښتلو کی ستونزی درلودی					
14	را ته سخته دی چی اجتماعی فعالیتونو ته وخت پیدا کړم					
15	کافی اندازی سره می د اجتماعی فعالیتونو لپاره انرژی نه درلوده					

	د فکر کولو فعالیت	هیڅکله	تقریبا هیڅکله	کله ناکله	کله کله	تقریبا همیشه
16	را ته سخته وه چی خپل فکری تمرکزپه اشیاوو وساتم					
17	را ته سخته وه چی د نورو خلکو راته ویلی شوی خبری په یاد وساتم					
18	را ته سخته وه څه می چی آوریدلی دی په یاد وساتم					
19	را ته سخته وه چی په ګړندی شکل فکر وکړم					
20	را ته سخته وه په کومه موضوع می چی فکر کاوه په یاد راولم					

	اریکې	هیڅکله	تقریبا هیڅکله	کله ناکله	کله کله	تقریبا همیشه
21	داسې احساس مې درلود چې څوک زما د کورنۍ وضیعت نه درک کوي					
22	را ته سخته وه تر څو د خپل ماشوم د صحت په هکله نورو سره خبرې وکړم					
23	را ته سخته وه تر څو خپل احساس داکترانو او نرسانو ته بیان کړم					

په تیرې یوې میاشت کې، ستاسو د ماشوم د صحت نتیجې ته په کتو، تاسې په کومه اندازه مشکلات درلودل:

	تشویش	هیڅکله	تقریبا هیڅکله	کله ناکله	کله کله	تقریبا همیشه
24	په تشویش وم چې آیا زما د ماشوم طبی تداوی به نتیجه ورکړي او که نه					
25	د خپل ماشوم دداروو او طبی تداوی د جانبي عوارضو په هکله مې تشویش درلود					
26	په تشویش کې وم چې د نورو خلکو عکس العمل زما د ماشوم د وضیعت په هکله څنګه وي					
27	په تشویش وم					
28	د خپل ماشوم د راتلونکې په هکله مې تشویش درلود					

لاندې یو لیست دی چې کیدای شي ستاسې د کورنۍ یو مشکل وي. لطفا مونږ ته ووایې چې په کومه اندازه لاندې مشکلاتو څخه تاسې او ستاسې کورنۍ ته په تیره یوه میاشت کې واقع شوي دي.

په تیرې یوې میاشت کې، ستاسو د ماشوم د صحت نتیجې ته په کتو، تاسې په کومه اندازه مشکلات درلودل

	ورزنۍ فعالیتونه	هیڅکله	تقریبا هیڅکله	کله ناکله	کله کله	تقریبا همیشه
29	کورنۍ فعالیتونو ډیر زیار وخت غوښتلو					
30	را ته سخته وه تر څو د خپل کور کارونو لپاره وخت پیدا کړم					
31	د کور د کارونو په اجرا کولو کې مې احساس ستړیا کوله					

	کورنۍ ارتباطات	هیڅکله	تقریبا هیڅکله	کله ناکله	کله کله	تقریبا همیشه
32	د کورنۍ د غړو تر منځ د ارتباطاتو کم والي					

33	د کورنۍ د غړیو تر منځ شخړې					
34	په کورنۍ شکل سره په تصمیم نیولو کې مشکل					
35	د کورنۍ مشکلاتو د حلولو لپاره د یوې کورنۍ په شکل مشکل درلود					
36	د کورنۍ د غړو تر منځ د تشویش او اضطراب موجودیت					

PedsQL Family Impact - English

Instructions

- This survey seeks to understand how caregivers feel about caring for their child.
- You will perform this survey on caregivers who are participating in the program (i) before the program starts, and (ii) after the program is complete.
- Before doing the survey:
  - Provide participants with the Plain Language Statement and explain what the *Mighty Children* program is, and that we want to evaluate it.
  - Complete the Consent Form, and obtain verbal consent.
- Explain to participants:
  - Families of children sometimes have special concerns or difficulties because of the child's health.
  - This survey contains a list of things that might be a problem for you and your family.
  - Please tell us how much of a problem each one has been for you during the past ONE month: never, almost never, sometimes, often, almost always.
  - There are no right or wrong answers.
  - If you do not understand a question, please ask for help.
  - Check understanding. Check for questions.
- Then proceed with the survey, reading the statement and the options for each question, and marking the participant's response.

Never	Almost never	Sometimes	Often	Almost always

In the past ONE MONTH, as a result of your child's health, how much of a problem have YOU had with:

	Physical functioning	Never	Almost never	Sometimes	Often	Almost always
1	I feel tired during the day					
2	I feel tired when I wake up in the morning					
3	I feel too tired to do the things I like to do					
4	I get headaches					
5	I feel physically weak					
6	I feel sick to my stomach					

In the past ONE MONTH, as a result of your child's health, how much of a problem have YOU had with:

	Emotional functioning	Never	Almost never	Sometimes	Often	Almost always
7	I feel anxious					
8	I feel sad					
9	I feel angry					
10	I feel frustrated					
11	I feel helpless or hopeless					

	Social functioning	Never	Almost never	Sometimes	Often	Almost always
12	I feel isolated from others					
13	I have trouble getting support from others					
14	It is hard to find time for social activities					
15	I do not have enough energy for social activities					

	Cognitive functioning	Never	Almost never	Sometimes	Often	Almost always
16	It is hard for me to keep my attention on things					
17	It is hard for me to remember what people tell me					
18	It is hard for me to remember what I just heard					
19	It is hard for me to think quickly					
20	I have trouble remembering what I was thinking					

	Communication	Never	Almost never	Sometimes	Often	Almost always
21	I feel that others do not understand my family's situation					
22	It is hard for me to talk about my child's health with others					
23	It is hard for me to tell doctors and nurses how I feel					

In the past ONE MONTH, as a result of your child's health, how much of a problem have YOU had with:

	Worry	Never	Almost never	Sometimes	Often	Almost always
24	I worry about whether or not my child's medical treatments are working					
25	I worry about the side effects of my child's medications/medical treatments					
26	I worry about how others will react to my child's condition					
27	I worry about how my child's illness is affecting other family members					
28	I worry about my child's future					

Below is a list of things that might be a problem for your family. Please tell us how much of a problem each one has been for your family during the past one month.

In the past ONE MONTH, as a result of your child's health, how much of a problem as YOUR FAMILY had with:

	Daily activities	Never	Almost never	Sometimes	Often	Almost always
29	Family activities taking more time and effort					
30	Difficulty finding time to finish household tasks					
31	Feeling too tired to finish household tasks					

	Family relationships	Never	Almost never	Sometimes	Often	Almost always
32	Lack of communication between family members					
33	Conflicts between family members					
34	Difficulty making decisions together as a family					
35	Difficulty solving family problems together					
36	Stress or tension between family members					

Annex 6: piloting and adaptation of parenting sense of competency scale (PSOC)

The original PSOC was translated into Pashto and Dari however the pilot showed difficulty in understanding. Caregivers consistently ticket the far right hand box on the Likert scale. Examination of the items showed that each question was double barrelled (like “if…then…” statements), making it hard to understand and interpret.

Original PSOC - Dari

تشریحات

- این نظرسنجی می کوشد تا درک کند که مراقبان نسبت به مراقبت از طفل خود چه احساسی دارند.
- شما این نظرسنجی را در مورد مراقبان مشارکت کننده در برنامه (i) قبل از شروع برنامه و (ii) بعد از اتمام برنامه انجام خواهید داد.
- قبل از انجام نظرسنجی:
    - بیانیه زبان ساده را به شرکت کنندگان ارائه دهید و توضیح دهید که برنامه اطفال قدرت مند چیست و ما می خواهیم آن را ارزیابی کنیم.
    - فورم رضایت را تکمیل کرده و رضایت کلامی را بدست آورید.
- برای شرکت کنندگان توضیح دهید:
    - این تحقیق با هدف درک نحوه احساس فرزندپروری انجام شده است.
    - این شامل 17 سوال است ، و می پرسد که آیا شما: به شدت مخالف ، تا حدودی مخالف ، مخالف ، موافق ، تا حدودی موافق ، به شدت موافق هستید.
    - هیچ پاسخ درست یا غلطی وجود ندارد.
    - اگر سوالی را نهفهمیدید ، لطفاً کمک بخواهید.

فهمیدن را بررسی کنید. سؤالات را بررسی کنید.

- سپس با استفاده از نظرسنجی ، خواندن بیانیه و گزینه های مربوط به هر سوال ، و پاسخ شرکت کننده را علامت گذاری کنید.

به شدت مخالف	تا حدودی مخالفم	مخالف	موافق	تا حدودی موافق	کاملا موافقم
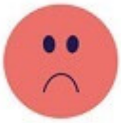	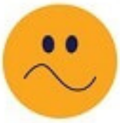	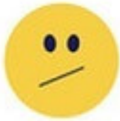	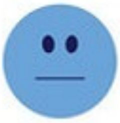	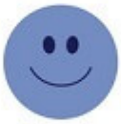	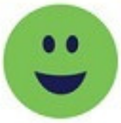

لطفاً میزان اظهار نظر یا مخالفت با اظهارات زیر را ارزیابی کنید:

		به شدت مخالف	تا حدودی مخالفم	مخالف	موافق	تا حدودی موافق	کاملا موافقم

1	مشکلات مراقبت از کودک به آسانی حل می شود که شما بدانید که اعمال شما چه تاثیری روی فرزند شما می گذارد ، فهم و تفسیری که من به دست آورده ام.						
2	اگرچه پدر و مادر بودن می تواند پاداش دهنده باشد ، در حالی که فرزند من در سن فعلی خود است ، ناامید شده ام.						
3	من به همان روشی که صبح از خواب بیدار می شوم به خواب می روم ، احساس می کنم نتیجه نگرفته ام.						
4	من نمی دانم چرا اینطور است ، اما بعضی اوقات وقتی قرار است کنترل داشته باشم ، امااحساس می کنم که بیشتر شخصا" خودم تحت تاثیر امده ام						
5	مادر من از من بهتر اماده بود بخاطر مادری کردن به طفل های خود						
6	من یک مثال خوب برای یک مادر جدید باشم تا وی بتواند عنوان والدین خوبی باشد.						
7	والدین بودن یک عمل کردنی هست و هر مشکل به راحتی حل می شود.						
8	مشکل والدین بودن این است که نمی داند کار خوبی انجام می دهید یا بد؟						
9	بعضی اوقات احساس می کنم هیچ کاری را انجام داده ام.						
10	من توقع شخصی خودم را برای براورد تخصص در مراقبت از فرزندم بدست اورده ام.						
11	کسی که از اذیت و تکلیف طفل من اگاهی بیشتر از هر کسی دیگر دارد خودم می باشد.						

12	استعدادها و علاقه های من در زمینه های دیگر است ،نه اینکه مادر باشم						
13	با نظرداشت اینکه چی مدتی مادر بوده ام ، با این نقش کاملاً آشنا هستم.						
14	اگر مادر بودن فرزند تنها جالب تر میبود ، من انگیزه می گرفتم که به عنوان والدین بتوانم کار بهتری داشته باشم.						
15	من صادقانه اعتقاد دارم که من تمام مهارت های لازم برای داشتن یک مادر خوب برای فرزندم را دارم.						
16	والدین بودن باعث تنش و اضطراب من می شود.						
17	مادر بودن خودش یک پاداش است.						

Original Parenting Sense of Competence Scale (PSOC) – (Pashto)

لارښوونې:

- د دی سروی په مرسته غواړو چی مراقبت کونکی د خپل ماشوم مراقبت په هکله څه احساس لریږ
- تاسی دا سروی په هغو کسانو تطبیق کوی چی په دی پروګرام کی ګډون کوی i)) د پروګرام پیل څخه وړاندی(ii) او د پروګرام د ختم څخه ورستهږ
- مخکی د سروی کولو څخه:
  - د ګډونکونو لپاره په ډیره عامیانه ژبه بیانیه آماده او تشریح ګړی چی د بیاوړی ماشومانو پروګرام څه شی دی، او مونږ غواړو دا پروګرام ارزیابی کړوږ
  - د رضایت فورم تکمیل او شفایی رضایت لاس ته راوړی.
- ګدونکونکو ته تشریح کړی چی:
  - د دی سروی هدف دا دی چی تر څو پوه شو چی تاسو منحیث د والدین څّه احساس کوی
  - شامل د ۱۷ پوښتنو دی، او پوښتنه وکړی چی ایا تاسی: کاملا مخالف، تر یو حده مخالف، مخالف، موافق، تر یو حده موافق او کاملا موافق یی
  - په لاندی پوښتنو کی هیڅ یو صحیح او غلطه پوښتنه نشته
  - که په کومه پوښتنه پوه نشوی کولای شی بیا ځلی یی وپوښتی
  - د ګډونکونو فهم ارزیابی کړی او وګوری چی کومه پوښتنه لری او که نه
- ورسته د یوی نظر پوښتنی، مربوطه ګوزینو او د بیاینیو په ویلو هر سوال او ځواب علامه ګذاری وکړی

کاملا مخالف	تر یوه حده مخالف	مخالف	موافق	تر یوه حده موافق	کاملا موافق

لطفا د خپل د اظهار نظر اندازه معلومه کړی چی په کومه اندازه د لاندی بیانیو سره موافق یا مخالف یاست

		کاملا مخالف	تر یوه حده مخالف	مخالف	موافق	تر یوه حده موافق	کاملا موافق
1	د ماشوم پاملرنه ډیر آسانه دی چی حل شی هر هغه یو ځلی. چی تاسی په د ی وپوهیدلی چی ستاسی هر عمل ښنګه ستاسی ماشوم متاثره کوی، دا هغه فهم اوهغه پوهه ده چی ما لاس ته راوړی.						
2	که څه هم والدین کیدی شي ګټور وي ، زه اوس خپه یم په داسې حال کې چې زما ماشوم په اوسني عمر کې دی.						
3	زه هغه دول چی ویده کیزم او یا هم پاسیزم داسی احساس کوم شی می لاس ته ندی راوړی						
4	زه نه پوهیزم چی ولی داسی ده,کله . ناکله زه چی باید په کنترول کی وسم, زه داسی احساس کوم چی زه اړول شوی یم.						
5	زما مور زما پر تله شه مورکیدو چمتو وه.						
6	زه به دیوی نوی مور لپاره شه نمونه اووسم چی تعقیب شی او زده کړه ورنه وشی اودا ددی اړه ولری چی پوه شی چی سنګه شه مور شی						
7	مور او پلار دی کیدلو کار ده او هر ه ستونزه شکل په آسانی سره حل کیږی						
8	د مور او پلار سخته ستونزه په دی کی ده چی نه پوهیزی چی آیا کوم کار چی له ماشوم سره کوی ګټه لری او که نلری						

9	کله ناکله داسی احساس کوم چی ما هیڅ ندی کړی.						
10	زه زما دخپل ماشوم د پاملرنی په برخه کی د مهارت زما شخصی تمه پوره کړی .						
11	هغه خوک چی وکولای شی پوه شی چی څه شی زما ماشوم زوروی هغه زه یم						
12	زما استعداد او علاقه په نورو برخو کی ده نظر په دی چی والدین ووسم.						
13	نظر په دی چی زه ډیر وخت کیږی چی مور یم له دی نقش سره ښه آشنایی لرم						
14	که د یوه ماشوم مور وسیدل یو جالبه کار وی، زه به ډیره تشویق شوی وی تر څو منحیث د والدین خپل کار په ښه توګه تر سره کړم						
15	زه صادقانه باور لرم چی زه ټول هغه مهم مهارتونه لرم چی یوه ښه مور خپل ماشوم ته ووسم.						
16	د مور پلار کیدل ما تنګوی او اندیشمن کوی.						
17	د یوه ماشوم مور اوسیدل خپله یو پاداش دی.						

Original Parenting Sense of Competence Scale (PSOC) - English

**Instructions**
● This survey seeks to understand how caregivers feel about caring for their child.<br>● You will perform this survey on caregivers who are participating in the program (i) before the program starts, and (ii) after the program is complete.<br>● Before doing the survey:<br>o Provide participants with the Plain Language Statement and explain what the *Mighty Children* program is, and that we want to evaluate it.<br>o Complete the Consent Form, and obtain verbal consent.<br>● Explain to participants:<br>o This survey aims to understand how you feel about parenting.<br>o It contains 17 questions, and asks whether you: strongly disagree, somewhat disagree, disagree, agree, somewhat agree, strongly agree.<br>o There are no right or wrong answers.<br>o If you do not understand a question, please ask for help.<br>o Check understanding. Check for questions.<br>● Then proceed with the survey, reading the statement and the options for each question, and marking the participant's response.

**Strongly disagree**	**Somewhat disagree**	**Disagree**	**Agree**	**Somewhat agree**	**Strongly agree**
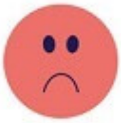	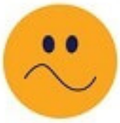	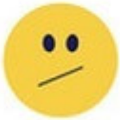	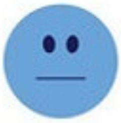	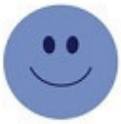	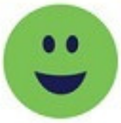

Please rate the extent to which you agree or disagree with the following statements:

		**Strongly disagree**	**Somewhat disagree**	**Disagree**	**Agree**	**Somewhat disagree**	**Strongly agree**
1	The problems of taking care of a child are easy to solve once you know how your actions affect your child, an understanding I have acquired.	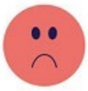	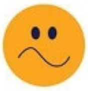	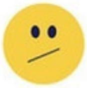	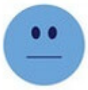	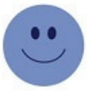	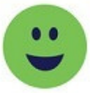
2	Even though being a parent could be rewarding, I am frustrated now while my child is at his/her present age.	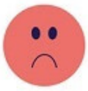	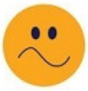	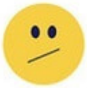	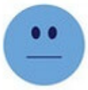	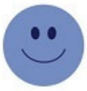	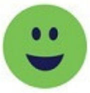

3	I go to bed the same way I wake up in the morning, feeling I have not accomplished a whole lot.						
4	I do not know why it is, but sometimes when I am supposed to be in control, I feel more like the one being manipulated.						
5	My mother was better prepared to be a good mother than I am.						
6	I would make a fine model for a new mother to follow in order to learn what she would need to know in order to be a good parent.						
7	Being a parent is manageable, and any problems are easily solved.						
8	A difficult problem in being a parent is not knowing whether you're doing a good job or a bad one.						
9	Sometimes I feel like I'm not getting anything done.						
10	I meet my own personal expectation for expertise in caring for my child.						
11	If anyone can find the answer to what is troubling my child, I am the one.						
12	My talents and interests are in other areas, not being a parent.						
13	Considering how long I have been a mother, I feel thoroughly familiar with this role.						

14	If being a mother of a child were only more interesting, I would be motivated to do a better job as a parent.						
15	I honestly believe I have all the skills necessary to be a good mother to my child.						
16	Being a parent makes me tense and anxious.						
17	Being a mother is a reward in itself.						

Modified PSOC

In discussion with the clinical psychologist involved in our program we simplified the tool down to 4 straightforward questions that directly asked about confidence, knowledge, and feelings.

We felt that the faces in the original version could have added confusion, particularly as the neutral face did not correspond with neutrality (corresponded with “agree”). Discussions with our implementation team in Afghanistan also revealed that there was poor understanding on how to complete a Likert scale like this. When expressed as percentages facilitators and caregivers seemed to have a better understanding of how to use the tool, yet the results did not triangulate with the qualitative outcomes (sees tables 10-12 below), the PedsQL results or the direct experience of facilitators. Possible problems identified included:

- As with the original, the modified version still did not allow for a “neutral” stance, as most Likert-type scales do
- Question 4 was the only question framed in the negative and may have resulted in confusion (parents reported becoming MORE anxious)

Since this tool was not formally re-piloted nor was it validated, we excluded it from inclusion in the paper.

Modified PSOC – English

Please rate the extent to which you agree or disagree with the following statements:

		Strongly disagree	Somewhat disagree	Disagree	Agree	Somewhat agree	Strongly agree
1	I feel confident in my ability to help my child grow and develop						
2	I know when something is wrong with my child						
3	Parenting is rewarding						
4	Parenting makes me tense and anxious						

Modified PSOC - Dari

(DARI) لطفاً میزان اظهار نظر یا مخالفت با اظهارات زیر را ارزیابی کنید:

		به شدت مخالف	تا حدودی مخالفم	مخالف	موافق	تا حدودی موافق	کاملا موافقم
1	من از توانایی خود برای کمک به رشد و رشد فرزندم احساس اطمینان می کنم						
2	من می دانم که چه مشکلی با فرزند من رخ داده است						
3	فرزندپروری جایزه دارد						
4	فرزندپروری باعث تنش و اضطراب می شود						

Table 10: Parenting sense of competency, mean (SD). Pooled data.

Question	Baseline	Endline	Mean difference (95% CI)	P
1. I feel confident in my ability to help my child grow and develop	4.4 (1.4)	4.3 (1.6)	-0.2 (2.2)	0.4476
2. I know when something is wrong with my child	4.5 (1.3)	4.6 (1.3)	0.1 (1.7)	0.4759
3. Parenting is rewarding*	5.3 (1.0)	5.6 (0.8)	0.4 (1.1)	0.0008
4. Parenting makes me tense and anxious*	4.7 (1.2)	5.3 (1.0)	0.6 (1.7)	0.0006

Note: *P value <0.05, using t-test. Total possible score is 6.

Table 11: Parenting sense of competency, by cohort. Mean (SD).

	Cohort 1		Cohort 2	
Question	Baseline	Endline	Baseline	Endline
I feel confident in my ability to help my child grow and develop	4.4 (1.5)	3.2 (1.4)*	4.5 (1.2)	5.4 (0.9)*
I know when something is wrong with my child	4.5 (1.5)	3.9 (1.3)*	4.5 (1)	5.3 (0.9)*
Parenting is rewarding	5 (1.1)	5.4 (0.9)*	5.6 (0.8)	5.9 (0.5)*
Parenting makes me tense and anxious	4.8 (1.2)	4.9 (1.2)	4.6 (1.2)	5.6 (0.7)*

Notes:
Total possible score is 6.
*P value <0.05, using t-test.

Table 12: Parenting sense of competency, by location. Mean (SD).

	Urban		Rural	
Question	Baseline	Endline	Baseline	Endline
I feel confident in my ability to help my child grow and develop	4.1 (1.6)	4.3 (1.5)	4.8 (1)	4.2 (1.7)
I know when something is wrong with my child	4.7 (1.1)	4.6 (1.4)	4.3 (1.4)	4.7 (1.3)*
Parenting is rewarding	5.5 (0.9)	5.6 (0.8)	5.1 (1.1)	5.7 (0.7)*
Parenting makes me tense and anxious	4.8 (1.2)	5.0 (1.2)	4.6 (1.2)	5.5 (0.8)*

Notes:
Total possible score is 6.
*P value <0.05, using t-test.

Annex 7: Caregiver focus group guide

Preparation

- Record the names of all the caregiver and child participants.
- Collect the feedback, responses, and activities that participants have completed during the *Mighty Children* program (e.g. drawings, photos, stories, etc.). You can use these to stimulate discussion, clarify points of view, and explore contrasting experiences.
- Remember: Our goal is to collect a variety of different stories and experiences. Encourage answers from every group member. If people are all agreeing – probe deeper to see if there are other perspectives hiding underneath. Encourage children's voices as well. Record emotional reactions.

Introduction

- Thank you for meeting with us. We appreciate your time and are really looking forward to learning from you. My name is __________, from the *Mighty Children* project team at RHDO.
- Thank you for participating in the *Mighty Children* project. You are some of the first people to take part in this program, and we are eager to hear what you thought about it so that we know how to make it better for others.
- You have given us lots of feedback during the program, which has been very valuable – thank you. Today we would like to have a 1 hour group discussion to learn more from you. We want to hear your experiences – good and bad. We want to hear all your different perspectives. Sometimes you will have similar perspectives, sometimes you will have very different perspectives. We want to hear it all.
- (Consent)
    - This group discussion will take approximately 60 minutes.
    - I will be asking you about your experience of the *Mighty Children* program, and I will be recording this meeting so that I accurately remember what you say.
    - We are also doing group discussions with other *Mighty Children* participants, and with the facilitators.
    - We will keep your identity, and others' identities, secret. But we may use some things that you say in our report so that other people can understand what the *Mighty Children* program was like.
    - If there are any questions you do not want to answer you are free to refuse. You can also leave the discussion and choose not to participate at any time.
    - Do you have any questions? Are you willing to take part?
- [Start voice recorder] I will now start the audio recording. For the benefit of the audio recording, can you each please state your name and repeat whether you give consent to take part? [Go around in a circle letting each person have a turn]

Prompting questions

**Main question**	**Optional additional questions**
• Thinking back to the first *Mighty Children* group meetings:<br><br>- Why did you choose to participate?<br><br>- What were your feelings and first reactions when you met the group?	"Did anyone have any other motivations or expectations for joining?"<br>Acknowledge emotions and ask for more details: "Interesting. Tell me more"
• Now you have completed the program:<br><br>- What has changed for you?<br><br>- What has changed for your child?<br><br>- What has changed for your family?	Probe for particular most significant changes (e.g. making new friends; seeing child differently; hope for child's future; parent confidence in caring for child; parent-child relationship; relationship with rest of family).<br>[Try and get responses from everyone]<br>Were there negative changes?
• If you were telling someone else about the *Mighty Children* program:<br><br>- How would you explain what it is?<br><br>- Who might benefit from the *Mighty Children* program? What sort of benefits?<br><br>- Are there people who might not benefit from the *Mighty Children* program? Or people who shouldn't do it?	(e.g. telling your friend or your sister)<br><br>[Benefits]<br><br>[Risks]<br><br>Who? Why? Do you know particular people who would like to do it<br>Can you give me an example?<br><br>Can you give me an example?<br><br>What training and support would you need to facilitate your own groups?
• If we were going to run the *Mighty Children* program again:	

- Would you recommend it to others?<br><br>- What was the most valuable part of the program for you?<br><br>- Were there any parts of the program that you would recommend cutting, or changing?<br><br>- Would you like to be trained to become a facilitator and help other families?<br><br>• Thank you. We are almost finished.<br><br>- We need to write a report about the *Mighty Children* program to the Government and hospital directors. What is the most important thing for us to tell them?<br><br>- Do you have any other things you would like to tell us about the *Mighty Children* program?	[Advocacy messages]<br><br>[Advice or final thoughts]

Annex 8: Facilitator Focus Group Guide

Preparation

- Record the names of all the facilitators.
- Facilitators may be nervous about sharing negative perspectives. Emphasise that you are here to listen and learn. They should not be judged or treated differently based on what they say.
- Remember: Our goal is to collect a variety of different stories and experiences. Encourage answers from every participant. If people are all agreeing – probe deeper to see if there are other perspectives hiding underneath. Record emotional responses.

Introduction

- Thank you for meeting with us. We appreciate your time and are really looking forward to learning from you.
- You know who I am. My name is __________, from the *Mighty Children* project team at RHDO. But today I am here to listen and learn from your experience with the *Mighty Children* program.
- Thank you for participating in the *Mighty Children* project. You are the first people to facilitate this program, and we are eager to hear what you thought about it so that we know how to make it better for others.
- You have given us lots of feedback during the program, which has been very valuable – thank you. Today we would like to have a 1 hour group discussion to learn more from you. We want to hear your experiences – good and bad. We want to hear all your different perspectives. Sometimes you will have similar perspectives, sometimes you will have very different perspectives. We want to hear it all. We want you to be completely honest and open with us. Nothing you say here will affect your current or future employment. We really just want to learn from you.
- (Consent)
    - This group discussion will take approximately 60 minutes.
    - I will be asking you about your experience of the *Mighty Children* program, and I will be recording this meeting so that I accurately remember what you say.
    - We will keep your identity, and others' identities, secret. But we may use some things that you say in our report so that other people can understand what the *Mighty Children* program was like.
    - If there are any questions you do not want to answer you are free to refuse. You can also leave the discussion and choose not to participate at any time.
    - Do you have any questions? Are you willing to take part?

[Start voice recorder] I will now start the audio recording. For the benefit of the audio recording, can you each please state your name and repeat whether you give consent to take part? [Go around in a circle letting each person have a turn]

Prompting questions

**Main question**	**Optional additional questions**
• Thinking back to the first *Mighty Children* group meetings:<br><br>- What did you expect? What experience had you had with children with chronic health conditions before?<br><br>- What were your feelings and reactions when you met your groups for the first time?	Acknowledge emotions and ask for more details: "Oh, that is interesting. Tell me more"
• Now you have completed the program:<br><br>- What changes did you see in the caregivers and children in your group?<br><br>- What has changed for you?	Probe for particular changes (e.g. making new friends; seeing child differently; hope for child's future; parent confidence in caring for child; parent-child relationship; relationship with rest of family).<br>Were there negative changes?<br><br>(e.g. telling your friend or your sister)
• If you were telling someone else about the *Mighty Children* program:<br><br>- How would you explain what it is?<br><br>- Who might benefit from the *Mighty Children* program? What sort of benefits?<br><br>- Are there people who might not benefit from the *Mighty Children* program? Or people who shouldn't do it?	[Benefits]<br><br>[Risks]<br><br>Who? Why? Do you know particular people who would like to do it<br>Can you give me an example?<br><br>Can you give me an example?
• If we were going to run the *Mighty Children* program again:	

- Would you recommend it to others?<br><br>- What was the most valuable part of the program for participants?<br><br>- Were there any parts of the program that you would recommend changing?<br><br>• <u>As a facilitator you had a lot of responsibility for looking after the people in your groups:</u><br><br>- How well prepared did you feel to run the groups? How well supported did you feel?<br><br>• <u>If we were wanting to recruit more facilitators, or perhaps identify caregivers from the program to be future facilitators:</u><br><br>- What are the most important attributes of a good facilitator?<br><br>• <u>Thank you. We are almost finished.</u><br><br>- We need to write a report about the *Mighty Children* program to the Government and hospital directors. What is the most important thing for us to tell them?<br><br>- Do you have any other things you would like to tell us about the *Mighty Children* program?	What things helped prepare you and support you? What was not helpful? What other things could have helped you feel better prepared and supported?<br><br>[Key attributes of a good facilitator] Personality, knowledge, skills, experience.<br>"What about experience caring for a child with a chronic health condition or disability?"<br><br>[Advocacy messages]<br><br>[Advice or final thoughts]

Annex 9: Early context, mechanism, outcome configurations

Context, mechanism, outcome configuration (CMOC) including all identified themes

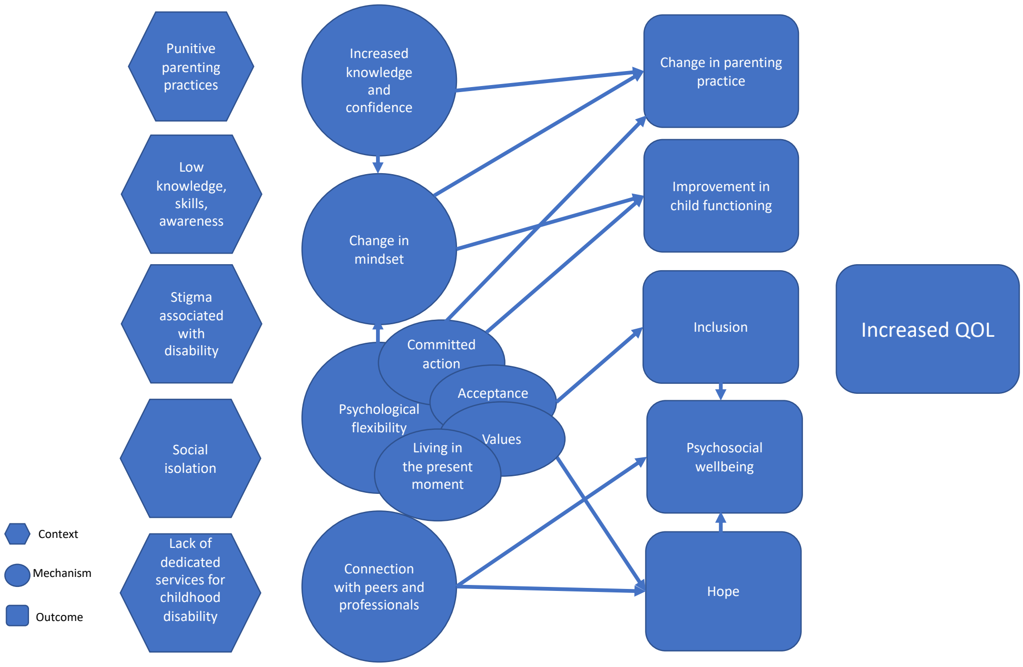

Change in mindset context, mechanism, outcome configuration (CMOC)

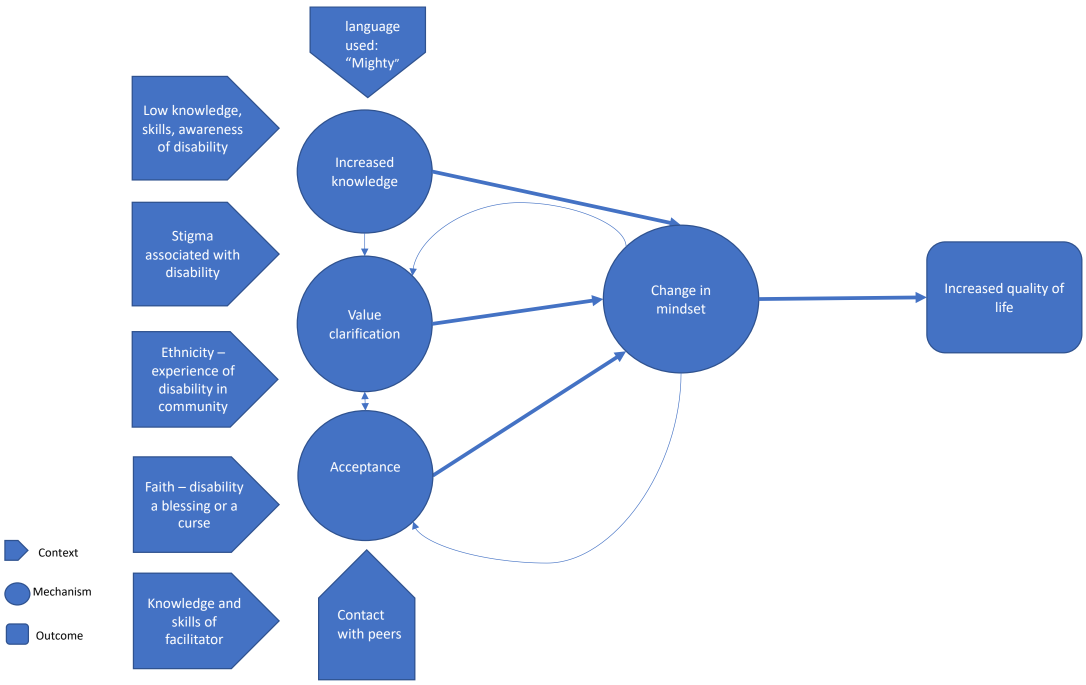

Proposed Context and Mechanisms for the key outcome change in mindset

Context	Mechanism	Key quote
Low knowledge, skills and awareness of disability<br><br>Stigma associated with disability<br><br>Faith – disability a blessing or a curse<br><br>Facilitator knowledge and skills	Gained knowledge, awareness. Caregivers and facilitators both reported an increase in their knowledge of disability.	*"We learnt that disabled children can also study and live like others. By doing this activity we realized that we can change our mentality regarding disabled children."* – Caregiver (anonymous), Group 3A<br><br>*"The teacher who taught you here, she also learned something, she understood the problems of mighty children, and she understood what is the position and role of mighty children in society. When they came here and we met them we knew how precious these children are, and this is a reality that we did not know before."* – Facilitator, Group 4
	Acceptance.	*"Before attending this program, there was frustration in my heart for my disabled child and this frustration nearly took away all my life, but after attending to this program, I learned how to be a positive or mighty mother. I learned how to take care of my mighty children, how to take care of my own self and now, as a powerful mother, I am ready to foster my mighty child, and fight*

		against the negative thoughts of society about my mighty child." – Caregiver 112, Group 5B
	Acceptance – moderated by the context of faith	*"we are living in the same house and same corridor with my in laws, she always used to say that my son is disabled and he cannot walk and Allah has given you such child then I [would] start fighting with her but now I deal with her with lots of patience and tell her that if Allah has given me such a disabled child, he also gave me the strength to take care of my child."* – Caregiver 91, Group 3B
	Value clarification. Many caregivers described how they now value their children more – and show this value to their children.	*"Previously we did not give any value to our [children] but right now you're giving them value. As you see my daughter does not have a hand. She cannot do her work properly, but as we attended to this program, this program taught us that disability is not weakness it is power and these children are mighty."* – Caregiver 124, Group 6B
	Inclusive language used by facilitators, changing language and attitudes of caregivers	*"… for that reason we call them mighty, to give them that confidence, that will help them grow emotionally and physically be able to do their own things."* – Facilitator, Group 1.

Change in parenting practice context, mechanism, outcome configuration (CMOC)

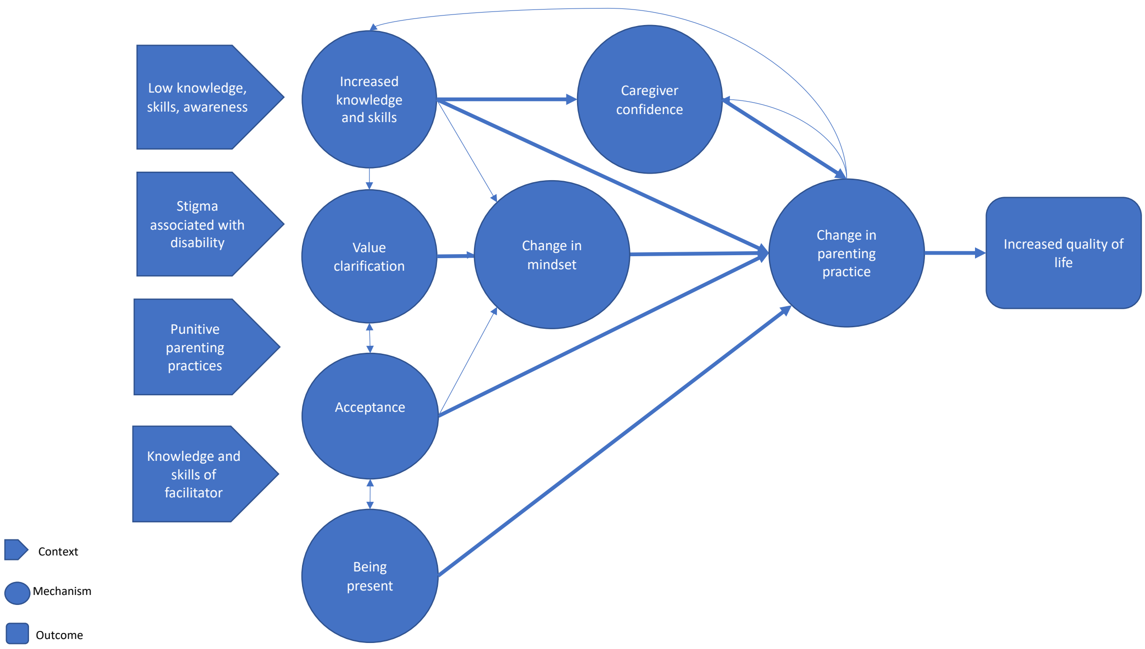

Proposed Context and Mechanisms for the key outcome change in parenting practice

Context	Mechanism	Key quote
Low knowledge, skills and awareness of disability<br><br>Stigma associated with disability<br><br>Culturally accepted punitive parenting practices<br><br>Facilitator knowledge and skills	By increasing caregiver **knowledge** and **skills**, some caregivers gained **confidence** and belief in themselves that they could take good care of their child	*"For being a confident mother, you respected doctors [sic] have helped me a lot. Your presence brought strength, energy, and confidence [which increased] our morale that we can take good care of our child."* – Caregiver 94, Group 3B
	Through **acceptance**, caregivers can live with their difficult emotions to behave in accordance with their values	*"Previously, I beat N_ a lot, I didn't take care of her properly, I only thought about myself not thought about N_, but after coming to this program, I found patience, I found power to take care of myself and N_. Right now, I take care of her in a very good way. I love her, I help her to improve and become someone because disability is not a sin, it is power that*

		can take you to your dreams." – Caregiver 127, Group 6B
	**Value clarification**. Choosing to place their child above all else, to be a "mighty mother".	*"In the beginning, I didn't take care of my children, but now I take care of her a lot... In the past, she was not important for me. She always sat in a corner and I was busy with house work, but now, I give her priority over everything else."* – Caregiver 125, Group 6B
	**Mindfulness.** Through living in the present moment, caregivers reported being able to diffuse difficult thoughts/ emotions and enjoy moments of joy in each day.<br><br>Facilitators observed emotional changes in caregivers and connected them to changes in behaviour towards their children	*"I have changed a lot when I became angry, I don't beat or shout to my children, I only go out take some fresh air and make my mind calm then I come back, and I start playing with them. These were the changes that happened in me after coming to this program."* – Caregiver 121, Group 6B<br><br>*"… at the beginning they were very mean, they were impatient and would get angry, for any mistake they [the child] would make, they would get angry, but after these sessions their behaviours have changed, they are more gentle and have good interaction with them, and they are more patient with their children…"* – Facilitator, Group 4

Inclusion context, mechanism, outcome configuration (CMOC)

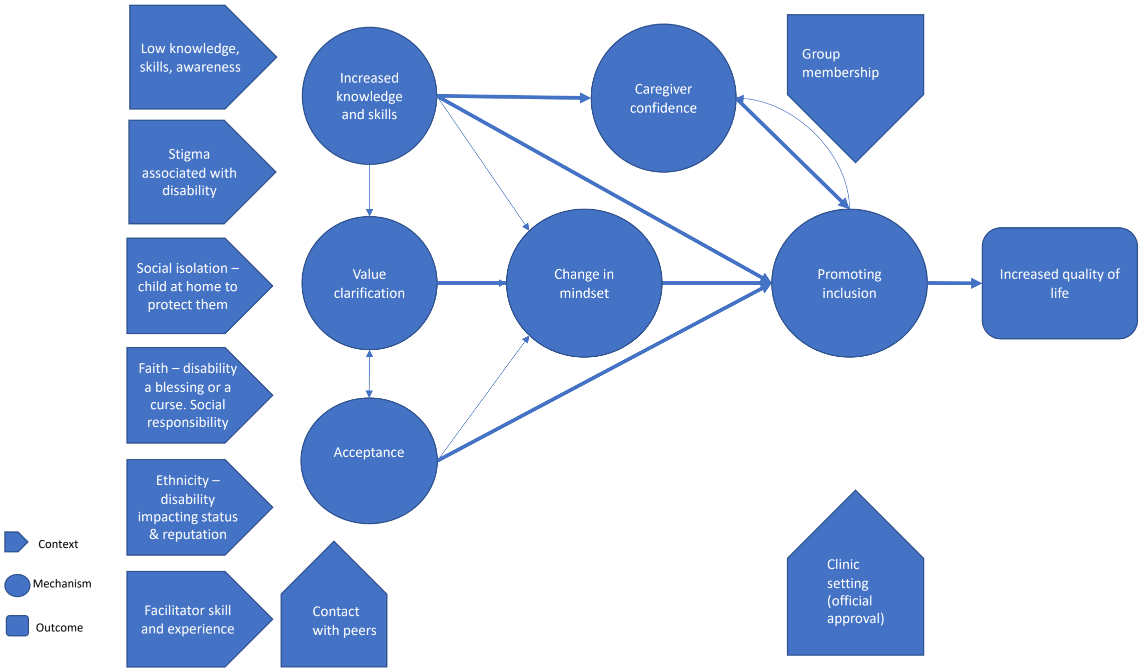

Caregiver and child psychosocial wellbeing context, mechanism, outcome configuration (CMOC)

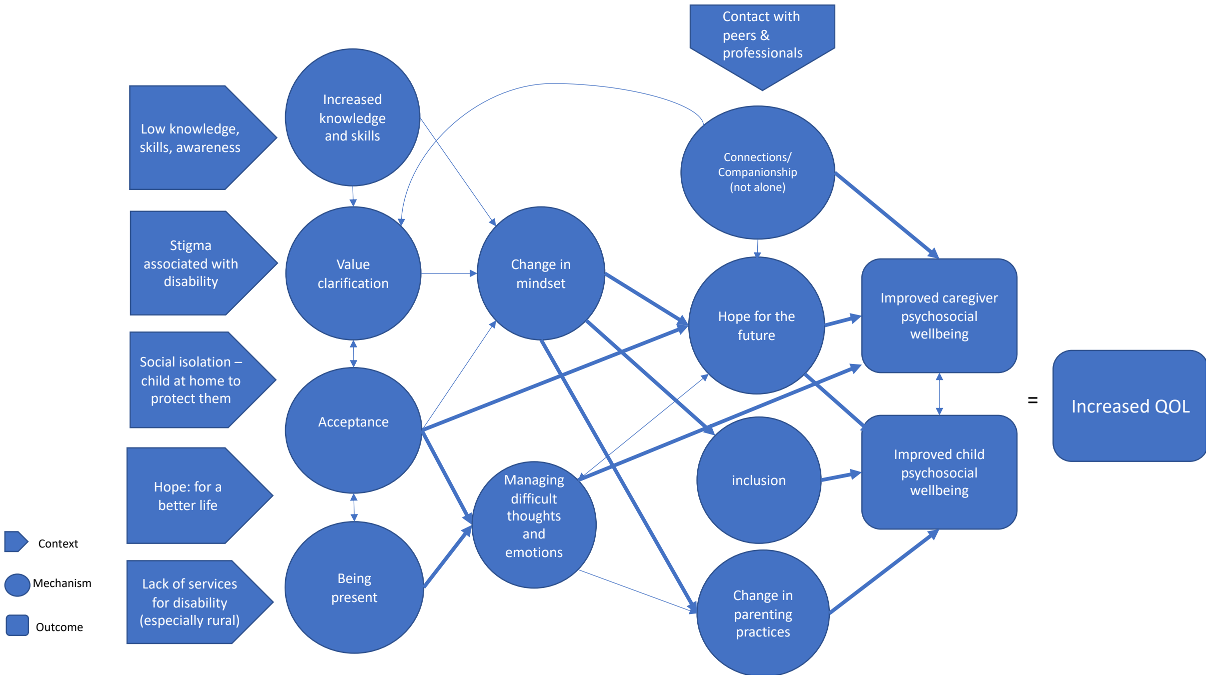

Annex 10: Author Reflexivity Statement

Study conceptualisation

1 How does this study address local research and policy priorities?

At the time of the study conceptualisation, The Afghanistan government had ratified both the United Nations Convention on the Rights of Persons with Disabilities (CRPD)(1), Convention on the Rights of the Child(2) and had a number of policy documents outlining the strategic plan for disability care in Afghanistan(3, 4) including supporting research in the disability sector.

2 How were local researchers involved in study design?

From project conception a local advisory board was established to provide project oversight, technical guidance and facilitate possible future scale up. Members included representatives from the Afghanistan ministry of health, major non-governmental actors involved in disability care, hospitals and local researchers (MFA and NA).

In December 2019 the Mighty Children project team met in Tashkent, Uzbekistan, to review formative research and discuss program development for the Afghan context. Local research and implementation partners (MFA and NA) as well as representatives from the MOPH attended this meeting to provide input into developing the program and to support coordinated implementation. MFA and NA were local doctors, researchers, and implementation partners. The study included a team of researchers from a high-income country who had extensive experience in conducting and supervising research collaborations in low income countries (HRG, NE, KM, AM).

Research management

3 How has funding been used to support the local research team(s)?

The *Mighty Children* program was funded by Grand Challenges Canada, through the "Creating Hope in Conflict: a Humanitarian Grand Challenge" scheme, sponsored by the U.S. Agency for International Development (USAID), the UK department for international development (DFID), and the Ministry of Foreign Affairs of the Netherlands. Grant ID: R-HGC-POC-1904-24744. These funds were used in Afghanistan for salaries for the local research team as well as all other implementation costs (eg printing of materials, provision of food during group sessions, facilitator training, purchase of heaters from group meeting rooms).

Data acquisition and analysis

4 How are research staff who conducted data collection acknowledged?

Those research staff who collected data who are not in the authorship group are acknowledged in the acknowledgements section of the manuscript.

5 How have members of the research partnership been provided with access to study data?

Members of the authorship groups involved in data collection, entry and analysis had redcap database logins.

6 How were data used to develop analytical skills within the partnership?

All authors were involved in discussions around developing qualitative themes and interpretation of both qualitative and quantitative data.

Data interpretation

7 How have research partners collaborated in interpreting study data?

Weekly meetings were held between the Afghanistan and Australian teams during project implementation then fortnightly during data entry and analysis to discuss emerging themes, clarify questions and interpret results.

Drafting and revising for intellectual content

8 How were research partners supported to develop writing skills?

All members of the authorship group were encouraged to share their thoughts both written and verbal which were incorporated into the manuscript by the primary author (NE). NE is an early career academic who had support in developing analytic and writing skills from senior academics (HRG, KM) from the authorship group.

9 How will research products be shared to address local needs?

The manuscript will be published in an open access journal allowing interested researchers in Afghanistan and beyond to access the results. While preliminary results were shared with all partners and stakeholders including the Afghanistan ministry of Health, our post-publication results dissemination plan was disrupted by the Taliban take-over of Afghanistan.

Authorship

10 How is the leadership, contribution and ownership of this work by LMIC researchers recognised within the authorship?

We have strong representation within our Authorship group of researchers based in Afghanistan. Their essential contribution is outlined in the contributor statement.

11 How have early career researchers across the partnership been included

within the authorship team?

Early career researchers have been included in program development (NE,NA), data collection and management (SZ) and in authoring the manuscript (NE).

12 How has gender balance been addressed within the authorship?

Many of the authorship group are female (NE, NA, SZ, AM, KM).

Training

13 How has the project contributed to training of LMIC researchers?

The project included training LMIC researchers in qualitative focus group interviewing skills (NA,SZ) as well as the process of data interpretation and manuscript development.

Infrastructure

14 How has the project contributed to improvements in local infrastructure?

The project had no direct contributions in improvements in local infrastructure.

Governance

15 What safeguarding procedures were used to protect local study participants and researchers?

All local study participants (and researchers who provided qualitative data) provided informed consent to participate in the study including consent for the use of their **de-identified** data. Participants were provided with both written information in Pashto/Dari (as appropriate) as well as verbal information given low literacy in the areas we were working. The verbal consent process was scripted to ensure all important information was consistently covered. It was made clear to participants that they could withdraw their consent at any time. Data was stored according to the data management plan in our study protocol including locking hard-copy data in locked cabinets and soft-copy data on password protected devices.

1. UN General Assembly. Convention on the Rights of Persons with Disabilities. United Nations; 2007.
2. UN General Assembly. Convention on the Rights of the Child. United Nations; 1989.
3. Islamic Republic of Afghanistan Ministry of Public Health. National Strategic Plan for Disability Prevention and Physical Rehabilitation 2021 - 2024. 2021.
4. Islamic Republic of Afghanistan Ministry of Public Health General Directorate of Preventive Medicine Primary Health Care Directorate Disability and Rehabilitation Department. National Strategic Plan for Disability Prevention and Physical Rehabilitation. (2017-2020). 2017.
